# Common Elements of Practice, Process and Implementation in Out-of-School-Time Academic Interventions for At-risk Children: a Systematic Review

**DOI:** 10.1007/s11121-020-01091-w

**Published:** 2020-02-04

**Authors:** Thomas Engell, Benedicte Kirkøen, Karianne Thune Hammerstrøm, Hege Kornør, Kristine Horseng Ludvigsen, Kristine Amlund Hagen

**Affiliations:** 1 Regional Centre for Child and Adolescent Mental Health, Eastern and Southern Norway, Oslo, Norway; 2 Department of Psychology, University of Oslo, Oslo, Norway; 3 Norwegian Institute of Public Health, Oslo, Norway; 4 Norwegian Center for Child Behavioral Development, Regional Centre for Child and Adolescent Mental Health, Eastern and Southern Norway, Oslo, Norway

**Keywords:** Academic interventions, Common elements, Practice elements, Process elements, Implementation elements, Children at risk, Primary school children, OSTA interventions

## Abstract

Academic achievement is a strong preventive factor against marginalization. Children at risk of academic failure and drop out can benefit from out-of-school-time academic (OSTA) interventions. Wide-scaled implementation and sustainment of effective interventions remain a struggle across education, welfare, and health. The need for approaches to increase implementability, effectiveness, and efficiency of interventions is pressing. Advancements in the field of education and mental health suggest identifying and studying discrete elements that are common across interventions for the purpose of hypothesis generation, intervention optimization, design improvement, and implementation. This review identified OSTA interventions for primary school children at risk of academic failure. Common elements methodology was used to code practice elements (*n =* 62), process elements (*n =* 49), and implementation elements (*n =* 36) in 30 effective and six ineffective OSTA interventions in matrices. Based on frequency counts, common practice, process, and implementation elements across the interventions were identified, and given frequency count values (FV) reflecting how often elements were included in effective studies as opposed to in ineffective studies. The five common practice elements with the highest FVs were homework support, training in positive parental school involvement, positive reinforcement, structured tutoring, and psychoeducation. The most common process element was regular support to intervention receiver, and the most common implementation element was quality monitoring. Common combinations of elements were also identified and given FVs. Results from this review can inform efforts to design or optimize OSTA interventions, and inform education, implementation, and practice to improve academic achievement for children at risk.

**Electronic supplementary material:**

The online version of this article (10.1007/s11121-020-01091-w) contains supplementary material, which is available to authorized users.

## Introduction

Poor academic achievement and school dropout are among the unfavorable outcomes experienced by children exposed to poverty, unstable home environments, involvement with child protection services, and poor parenting skills (OECD 2016). Children at risk often develop gaps in knowledge early in their academic careers. These early educational shortcomings often exacerbate over time and contribute to academic failure and dropout in later school years (Sebba et al. 2015). Academic achievement is a strong protective factor against marginalization in adulthood (Johnson et al. 2010). Children at risk who achieve academically are less likely to experience illness, to use drugs, to engage in criminal behavior, and to become recipients of welfare services (Berlin et al. 2011). Thus, preventing academic failure can be valuable to children at risk, which in turn may result in social and economic returns for society at large (OECD 2016). Out-of-school-time academic (OSTA) interventions may be effective in promoting academic achievement for children at risk (Knopf et al. 2015). Interventions delivered outside of school hours avoid the potential stigma associated with receiving special education in class or being removed from the classrooms. OSTA interventions also do not replace the regular classroom curriculum. Furthermore, involving parents in academic interventions at home can improve children’s educational achievement (Wilder 2014).

OSTA interventions, such as Teach Your Children Well (Maloney et al. 1990) or On The Way Home (Trout et al. 2012), often consist of multiple academic and psychosocial elements. Some elements directly target academic skills (e.g., tutoring), some focus on behavior (e.g., use of homework contracts and routines), and others may target motivation and emotions (e.g., positive reinforcement and self-regulation). Typically, these elements are structured and sequenced following an instructive manual, and adopting the intervention includes comprehensive implementation strategies requiring infrastructure and resources to obtain and maintain intervention fidelity. Many OSTA interventions share these features with evidence-based psychosocial interventions for children and families. The well-engineered nature of many evidence-based interventions likely contributes to their effectiveness. However, their resource and implementation demands, multitude of elements, and structural rigor can make them complex to implement and sustain as intended (Hogue et al. 2017). In addition, they usually target single outcome domains. Schools and welfare services often require several different interventions to cover the width of educational and psychosocial outcomes they need to address, but successfully implementing multiple complex interventions is not always feasible. This offers some explanation as to why widespread adoption and population level impact from evidence-based interventions appear to be limited (Glasgow et al. 2012; Lau et al. 2015). To increase the reach of effective interventions at scale, there is a need for ways to decrease intervention complexity and improve *implementability* (feasibility, appropriateness, acceptability and usability, Lyon and Bruns 2019) without compromising effectiveness, and to identify interventions that can be effective across multiple outcome domains.

Disentangling interventions into discrete elements can facilitate re-design of interventions and alternative modes of delivery that are potentially less demanding to implement and sustain (e.g., single element practices or leaner combinations compared with more complex multi-element interventions). OSTA interventions are likely to share elements that may or may not be important for intervention effectiveness, and we do not know whether all practical and structural elements of an intervention are necessary. Further, there might be specific elements across interventions that have a stronger potential for effectiveness than others, and some might be effective across multiple outcome domains. To answer these questions, researchers can benefit from evidence-informed hypotheses about what the effective elements and combinations of elements are, and which are expendable. Elements that are frequently shared among effective interventions are more likely than less frequent elements to contribute to effectiveness. Identifying these *common elements* can inform studies of intervention optimization and design for improving implementability, efficiency, and effectiveness (Chorpita et al. 2011). To date, several studies have identified common elements of various psychosocial interventions (e.g., Hogue et al. 2017) and psychotherapy (e.g., Okamura et al. 2019). Results from these studies have been used for design of modular and element-based interventions tailored to individual needs (e.g., Murray et al. 2018), empirical testing of singular elements (e.g., Leijten et al. 2015), and to inform training and consultation in children’s mental health services (e.g., Dorsey et al. 2016). To our knowledge, no prior study has systematically identified the common elements of effective OSTA interventions for children at risk.

**Fig. 1:**
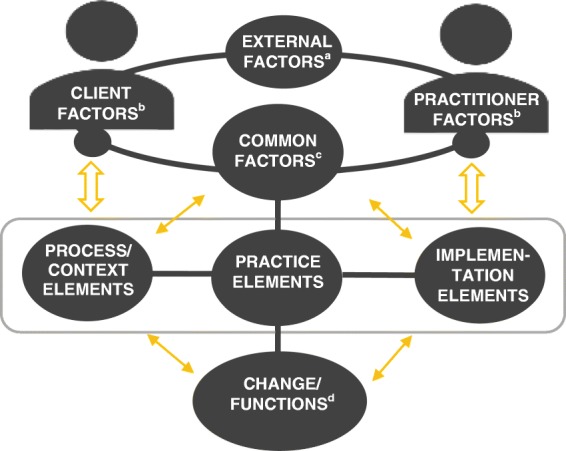
Factors and elements causing change mechanisms in an interaction between a practitioner and a client. Elements in focus in this review are placed inside the rectangular box. ^**a**^Examples of external factors can be social norms, culture, and government policies. ^b^Examples of client and practitioner factors can be personality, biology/genomics, values, motivation, and competence. ^c^Examples of common factors can be therapeutic alliance, allegiance, and epistemic trust. ^d^Functions refer to a proximal change that might serve as a mediator to a medial target outcome (such as motivation, engagement, or altered behavior). Figure created in MS word

In this review, we used a novel common elements methodology to identify discrete intervention contents and characteristics frequently shared by effective OSTA interventions. We distinguish between practice, process, and implementation elements. *Practice elements*, also known as *specific factors* in the psychotherapy literature (Mulder et al. 2017), are specific activities or actions used to evoke or influence an outcome (e.g., positive reinforcement). A practice element, however, might affect change differently depending on how, for whom, and under what circumstances it is delivered and implemented. *Process elements* cover these delivery forms and contexts (such as *home visitation* or *role-playing* in parent training)*. Implementation elements* are discrete strategies to facilitate or enable the delivery of practice and process elements (such as *ongoing training* or *tailoring to context*). Additionally, we identify common combinations of practice, process, and implementation elements in effective interventions. Analyses of frequencies do not merit conclusions about the effectiveness of elements. However, we assess frequencies of the most common elements and combinations in effective interventions across different academic outcomes, while also taking into account the frequencies with which they appear in ineffective or harmful interventions. This approach provides additional nuance to interpretation of common elements. The results can help generate new hypotheses about what combinations and interactions of elements, factors, and characteristics that are likely to cause, mediate, or moderate change in OSTA interventions across different academic outcomes (Fig. 1).

## Methods

### Research Questions

What are common practice, process, and implementation elements of effective OSTA interventions for primary school children at risk?

How frequently are the most common elements used in effective OSTA interventions, and how frequently are the common elements used in interventions without statistically significant effects (or with harmful effects) on academic achievement?

What are the most frequent combinations of common practice, process, and implementation elements used in effective OSTA interventions, also taking into account the frequency of common combinations in ineffective interventions?

### Eligibility (PICO)

Eligible populations included children attending primary school (aged 5–13 years) identified as being at risk of academic failure and/or dropout. This included both children identified through observed academic underachievement (e.g., students with low grade point averages or low scores on academic assessments) and those considered at risk based on their social or family background (e.g., children in foster care and children living in socioeconomically disadvantaged families). Studies on populations with developmental disabilities or other cognitive impairments were excluded.

Eligible interventions included those classified as out-of-school-time academic (OSTA) interventions that aimed to improve academic achievement. We defined an intervention as out-of-school-time when its core elements (i.e., the elements considered indispensable to the intervention) were delivered in an out-of-school environment and outside of school hours. We included methods of tutoring, mentoring, academic training, homework support, and parent training as OSTA interventions. We excluded home schooling used as a substitute for attending public school. We also excluded summer schools because we considered them more similar to a regular school compared with OSTA interventions. Further, regular assigned homework was excluded, as the interventions needed to offer something in addition to the regular curriculum. Finally, we excluded interventions specifically aimed at learning disorders such as dyslexia, aphasia, or processing disorders.

Eligible comparison conditions included no intervention, other academic interventions, and school-based interventions. Eligible primary outcomes were academic achievement measured either by grade point averages or assessments of academic skills in reading, math, or other school subjects. Eligible secondary outcomes were parental engagement/involvement in school and any adverse events or harms (e.g., stigma or missing out on leisure time activities due to receiving academic support, or anxiety due to being indicated as underachieving in school). We included studies with short- (< 2 months), middle- (2–12 months), and long-term (> 12 months) outcome assessments.

### Literature Search and Selection

We systematically searched MEDLINE (Ovid), PsycINFO (Ovid), PubMed, The Cochrane Library (CENTRAL, DARE), ERIC, ISI Web of Science (Science and Social Science Citation Index), Clinicaltrials.gov, OpenGrey, Social Science Research Network (SSRN), Google, and Google Scholar for published and unpublished studies and gray literature. We hand-searched the Campbell Collaboration Library, Youth Services Review, and What Works Clearinghouse. Search strategies for electronic databases included terms (both text words and subject headings) describing compensatory/extracurricular education, combined with terms describing academic achievement, as well as appropriate study designs. Complete search strategies are given in Online Resource 1. The search was conducted on April 01, 2016, without limits on language or publication year. Titles, abstracts, and full texts were reviewed for eligibility by two independent reviewers. Conflicts were resolved by discussion or a third reviewer.

### Risk of Bias Assessment

Using the criteria outlined in the Cochrane Handbook for Systematic Reviews of Interventions (Higgins and Green 2011), two review authors (TE and KTH) independently assessed the risk of bias in each study meeting the eligibility criteria. We rated each study at high, low, or unclear risk of bias across risk of bias domains. Disagreements were resolved by discussion. Only studies rated at low or unclear risk of bias across a majority of domains were included in the common elements analyses.

### Effectiveness Classification

We classified interventions in the included studies as either positively effective, ineffective, or negatively effective per outcome. For randomized controlled trials, we classified interventions as effective if at least one effect measure on a primary or secondary outcome was statistically significant (*p* < .05). For non-randomized controlled trials and interrupted time series, we classified interventions as effective if there was at least one statistically significant difference between the intervention group and the comparison group on a primary or secondary outcome, and there was a statistically significant pre to post change on the same outcome. Interventions that could not be classified as effective were classified as ineffective. An intervention classified as effective for one outcome (e.g., reading) could also be classified as ineffective for another outcome (e.g., math). Common elements analyses were done per outcome, and the coding procedure allowed for three different outcomes to be coded. The three primary or secondary outcomes that most frequently were significantly affected by an intervention were chosen for common elements coding and analyses.

### Gathering Study Information

In addition to publications identified in the database search, we searched the internet for intervention manuals to inform the data extraction for each included study. We used piloted forms to extract the following data: methods (study design, timing of outcome measures, whether intention-to-treat analyses were used), information about participants (age, gender, type of risk, number of participants, attrition, reach), details on interventions and control conditions, outcome measures, funding source, and publication type.

### Coding of Elements

We coded the elements using a manual developed by two of the authors (TE, HK, Online Resource 2), inspired by Chorpita and Daleiden’s (2009), distillation and matching procedure which combines data-mining techniques, frequency counts, and interaction-detection algorithms. In our review, we distinguish between *practice elements* (such as training in paired reading), *process elements* (such as home visitation to provide dyadic training in paired reading), and *implementation elements* (such as ongoing training to practitioners delivering training in paired reading). In addition, we adopted current classifications and definitions of implementation elements from the implementation science literature (Powell et al. 2015).

We coded elements in a Microsoft Excel matrix. Coding options (elements available for coding) were prepared, but not forced. Using consensus mapping with coders, elements that were anticipated to be included in studies were listed in the matrix a priori. During coding, coders were also encouraged to identify new unanticipated elements in addition to the listed in the matrix. New unanticipated elements were discussed and added if coders agreed they were different from prepared elements. Subsequently, coders reviewed the interventions again to look for unanticipated elements added during first round of coding. This procedure was chosen to reduce confirmatory bias and to facilitate discovery of novel elements. Four pairs of coders independently coded each intervention in separate matrices. Conflicts were resolved by discussion or a third coder. Percentage of agreement between each coding pair and all coders together was calculated based on each coder’s amount of coding input and amount of coding conflicts (differences between coder’s inputs). Information about coders is reported in Online Resource 1.

### Identification of Common Elements and Combinations

We counted frequencies to identify the most frequent practice elements of effective interventions. We then matched the practice elements with process and implementation elements and characteristics that were most frequently used with the practice elements when the interventions were effective. We also identified combinations of practice elements most frequently used in effective interventions. Further details are provided in Online Resource 2. All elements were given a frequency count corresponding with the number of times they were included in studies with significant positive effects on the three most frequently affected outcomes. To our knowledge, no cut-off exists to define what is considered *common* in a selection if interventions. Based on convenience, we defined the 25% most frequent elements in the included effective interventions as *common elements.*

### Ineffective Interventions and Frequency Count Values

We also coded practice elements in ineffective interventions. A traditional vote-counting procedure (Bushman and Wang 2009) was used to determine a frequency count value (FV). If a common practice element was included in an intervention classified as ineffective, a frequency count of one was deducted from the total frequency count of that practice element and from the process and implementation elements used in combination with that practice element. This approach provided a total FV reflecting how often the element was included in effective interventions minus the number of times it was included in ineffective interventions. If a common practice element was included in a harmful intervention (negative effect on outcome), a frequency count of two would have been deducted. However, no interventions with negative effects were identified in the review.

The vote-counting procedure was performed to reduce *popularity bias*, which can be defined as the tendency to include elements that are frequently used in interventions based on the element being perceived as important, regardless of the elements’ effectiveness, appropriateness, or frequency in ineffective interventions. FVs are, however, likely skewed because of publication bias (Easterbrook et al. 1991).

## Results

**Fig. 2:**
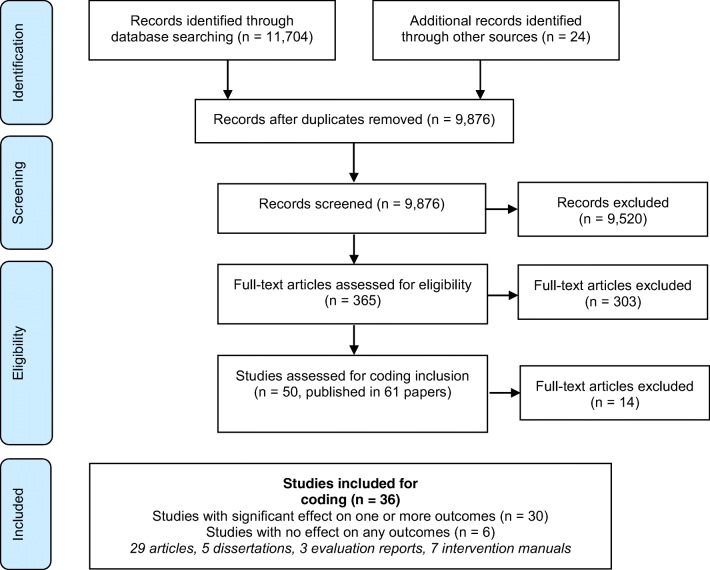
Prisma flow diagram depicting number of records identified, screened, assessed for eligibility, assessed for coding inclusion, excluded, and included. Figure created in MS word

As depicted in Fig. 2, we identified 50 eligible studies in 61 publications after reviewing 9.876 unique records. Titles and reasons for exclusions are given in Online Resource 1. Of these, two did not meet our risk of bias criteria, and 12 did not provide enough data to classify effectiveness. We included 36 independent studies of 30 effective interventions and 6 ineffective interventions for common elements analyses based on information from 29 articles, 5 dissertations, 3 evaluation reports, and 7 intervention manuals. Summaries of study characteristics are given in table 1 (available online).

### Included OSTA Interventions

#### Effective Interventions

Eleven effective interventions were parent mediated and typically included different parent training elements in academic involvement. Nine interventions were child-focused interventions including tutoring and other academic enhancement activities and support, and six interventions were combinations of the above. Three interventions were after school programs, one intervention targeted child self-regulation, and another targeted child self-esteem.

#### Ineffective Interventions

Six interventions were classified as ineffective. No studies reviewed reported negative or harmful effects. Five of these had positive trends or significant effects on at least one outcome measure but did not meet effectiveness classification criteria. Two interventions were after school tutoring and academic support programs, one after school program combined child tutoring and support with parent training, two interventions were parent-mediated child tutoring, and one after school program focused on sports and homework support. Risk of bias assessments of included studies and elaborate descriptions of study and intervention characteristics are available in Online Resource 1.

### Common Practice, Process, and Implementation Elements (Research Question 1)

We identified 62 discrete practice elements in 36 OSTA interventions for children at risk. The 25% most common practice elements were *structured tutoring*, training and guidance in *parental school involvementat home*, training and guidance in *homework support*, various forms of *literacy training*, *positive reinforcement* , *psychoeducation*, *correction* and *immediate feedback*, and *use of explicit goals*. Reading abilities (*n* = 21), mathematical abilities (*n* = 6), and grade point average (GPA, *n* = 6) were the three most frequently statistically significantly affected outcomes. Frequency counts for each common practice element per outcome are depicted in Table 1. Frequency counts for remaining practice elements are given in table 2 (available online).

**Table 1:** Common practice elements, common combinations of practice, process, and implementation elements, and frequencies in effective and ineffective interventions

**Common practice elements**	**Frequency counts**						**Elements used in combinations with common practice elements**		
Definitions	**Reading***(29 studies)*		**Math***(8 studies)*		**GPA***(6 studies)*		**Process elements** (FV^d^)	**Implementation elements** (FV)	**Practice elements** (FV)
**Homework support**^**b**^ *Guidance in; appropriate homework structure and discipline (1), homework instruction and support (2), and (3) homework environment*	+^a^	÷	+	÷	+	÷	• Delivered by professional (4 y. training) (12) • Received by caregiver (11) • Multi-element (10) • Regularly support to receiver (9) • 1on1 delivery (8)	• Quality monitoring (7^d^) • Provide ongoing consultation (7) • Conduct educational meetings (6) • Conduct ongoing training (5) • Involve end-users (4) • Remind practitioners (4)	• Training in parental school involvement at home (11) • Structured tutoring (8) • Use of positive reinforcement (8) • Use of incentives/rewards (7) • Monitor performance (7) • Correction and feedback (FV=7)
	12		1						
	**FV=12** (*n* = 1338^c^)		**FV=1** (*n* = 105)						
**Training in parental school involvement at home** *Training or guidance in any form of engagement by caregivers to support a child academically at home*	10		2		3		• Received by caregiver (14) • Delivered by professional (13) • Regularly support to receiver (12) • Use of organizational material (11) • Use of educational material (10) • Multi-element (10)	• Quality monitoring (13) • Distribute educational materials (12) • Provide ongoing consultation (8) • Remind practitioners (5) • Clinical supervision (4) • Conduct ongoing training (4) • Centralized technical assistance (4) • Involve end-users (4)	• Homework support (11) • Psychoeducation (10) • Use of positive reinforcement (9) • Use of incentives/rewards (8) • Structured tutoring (8)
	**FV=10** (*n* = 1194)		**FV=2** (*n* = 177)		**FV=3** (*n* = 56)				
**Positive reinforcement and/or incentives**^**b**^ *Use of positive responses (1) or incentives (2) to welcomed behaviors or performances*	11	1	4		2	1	• Delivered by caregiver (13) • 1on1 delivery (12) • Use of rewards or incentives (11) • Regular support to deliverer (11) • Delivered at home (11) • Multi-element (9) • Less than 3 hours a week, more 4 months (9) • Use of educational material (9)	• Quality monitoring (11) • Provide ongoing consultation (9) • Distribute educational materials (7) • Remind practitioners (5) • Conduct educational meetings (5) • Involve end-users (4)	• Parental school involvement at home (10) • Homework support (8) • Correction and feedback (7) • Monitor performance (7) • Structured tutoring (7)
	**FV=10** (*n* = 771)		**FV=4** (*n* = 331)		**FV=1** (*n* = 100)				
**Structured tutoring**^**b**^ *Direct Instruction from a teacher or an instructor (1), or interactional learning (2) following a curriculum or more or less stringent instruction*	14	3	5	3			• Repeated training (12) • Feedback on performance (12) • Use of educational material (11) • Direct instruction as delivery method (11) • Progressive difficulty (11) • Less than 3 hours a week, more 4 months (10)	• Quality monitoring (9) • Distribute educational materials (9) • Provide ongoing consultation (8) • Conduct ongoing training (5) • Involve end-users (5) • Conduct educational meetings (4) • Feedback in training (3)	• Training in parental school involvement at home (10) • Child reading aloud to someone (9) • Use of positive reinforcement and incentives (9) • Training in parental homework instruction (7)
	**FV=11** (*n* = 1458)		**FV= 2** (*n* = 1403)						
**Psychoeducation** Any form of empowerment and/or educating of the affected using "condition-specific" information.	7		2		2		• Received by caregiver (8) • Delivered by professional (8) • Delivered in group (5) • Less than 3 hours a week, less than four months (5) • Multi-element (5)	• Quality monitoring (5) • Provide ongoing consultation (4) • Distribute educational materials (4) • Conduct educational meetings (4)	• Parental school involvement at home (10) • Homework support (6) • Literacy training (5) • Positive reinforcement (5)
	**FV=7** (*n* = 771)		**FV=2** (*n* = 172)		**FV=2** (*n* = 56)				
**Correction and feedback** Using specific instruction based on behavior or performance to alter unwanted behavior or performance	7		4				• Delivered by caregiver (4) • Feedback on performance (4)	• Provide ongoing consultation (8) • Quality monitoring (6) • Distribute educational materials (5) • Clinical supervision (4) • Conduct educational meetings (4)	• Structured tutoring (8) • Positive reinforcement (7) • Literacy training (6) • Homework support (6) • Parental school involvement at home (6) • Use of explicit goals (5)
	**FV=7** (*n* = 1354)		**FV=4** (*n* = 1403)						
**Literacy training** Various literacy training techniques aggregated in one category^e^	11	4					• Repetitive training/instruction (10) • Less than 3 hours a week, more 4 months (9) • Use of educational material (8) Progressive (8)	• Provide ongoing consultation (10) • Quality monitoring (7) • Conduct ongoing training (7) • Conduct educational meetings (6) • Clinical supervision (4)	• Structured tutoring (12) • Parental school involvement at home (9) • Homework support (9) • Positive reinforcement (7) • Correction and feedback (5) • Playing reading game (5) • Discussion (5)
	**FV=7** (*n* = 1458)								
**Use of explicit goals** Targeting explicitly stated proximal or distal goals to be achieved by engaging in the intervention	5		3		1		• Received by child k 4-7 (7) • Less than 3 hours a week, more than 4 months (6) • Delivered at home (6) • Use of organizational material (6) • Regular support to deliverer (6)	• Provide ongoing consultation (6) • Quality monitoring (5) • Distribute educational materials (5)	• Correction and feedback (6) • Positive reinforcement (5) • Homework support (5) • Parental school involvement at home (4) • Structured tutoring (4)
	**FV=5** (*n* = 401)		**FV=3** (*n* = 1326)		**FV=1** (*n* = 77)				

^a^Frequency count value (FV) = frequency of the practice elements’ inclusion in effective interventions (+1) accounted for inclusion in ineffective interventions (-1)

^b^The common practice element is an aggregation of two closely related practice elements

^c^Total amount of participants in the studies where the practice element was used in an intervention

^d^The frequency count value of process elements used in combination with the practice element in effective interventions (+1) accounted for in ineffective interventions (-1)

^e^Reading aloud: +10, word recognition: +7, reading comprehension: +6, phonics training: +4, word decoding: +5, paired reading: +4. See Online Resource 1 for definitions

We identified 49 discrete process elements in the interventions. The most common process elements overall were *regularly support to receiver*, *use of educational material*, *delivered by professional(4 years of relevant education or more)*, *repeated training*, *received by caregiver*, *delivered by caregiver*, *low intensity*, and *long duration* (*less than3 h a week*, *more than* *4 months)*, *1-on-1 delivery*, and *multi-element intervention*. We identified 36 of the 73 pre-defined implementation elements used to implement the interventions. The most common implementation elements overall were *quality monitoring*, *providing ongoing consultation*, *distributing educational material*, *conducting educational meetings*, *clinical supervision*, *conduct ongoing training*, *use train the trainer*, and *involve end-users*.

Eleven unanticipated elements were identified and included during coding. One of these elements, *direct instruction* as delivery method, was a commonly used process element with effective structured tutoring (FV = 11). Frequency counts for all process and implementation elements are given in Online Resource 1. The mean number of coding inputs per intervention was 170.70 (SD = 97.50). Total coding agreement between coders was at 90.4%. Further coding statistics are provided in Online Resource 1.

### Common Elements of Effective and Ineffective Interventions (Research Question 2)

Five interventions classified as effective on one outcome were classified as ineffective on another outcome. Frequency counts for each common practice element’s inclusion in effective (+) and ineffective (÷) interventions per outcome category are depicted in Table 1. Frequency count values (inclusion in effective minus ineffective interventions, FVs) are given for each common practice element per outcome category. *Homework support* had the highest FV with 12 for reading, followed by *training in parental school involvement at home* and *positive reinforcement* with FVs of 10. *Positive reinforcement* and *correction and feedback* had the highest FVs for math with 4. Training in parental school involvement at home had the highest for GPA with FV of 3. Training and guidance in parental school involvement at home, positive reinforcement and praise, psychoeducation, and use of explicit goals were used in interventions with positive FVs across all three outcomes.

FVs of process and implementation elements used together with specific common practice elements are shown in parentheses in Table 1, meaning the FVs accounts for the number of times the process element was used in combination with the specific practice element in effective interventions subtracting the number of times it was used in ineffective interventions. Overall, process elements with peak FVs were *received by caregiver* (14), *delivered by professional* (13), *delivered by caregiver* (13), *1on1 delivery* (12), *repeated training* (12), and *feedback on performance* (12). Implementation elements with peak FVs were *quality monitoring* (13), *distributing educational materials* (12), and *ongoing consultation* (10). However, FVs of process and implementation elements are practice element–specific and vary according to what practice element they have been combined with. Structured tutoring had the biggest difference between frequency count and FV, with being ineffective in 3 out of 14 interventions on reading, and 3 out of 5 interventions on math. Literacy training had the second biggest difference with being ineffective in 4 out of 11 interventions on reading.

### Common Combinations of Elements (Research Question 3)

Commonness of combinations of elements in effective interventions as opposed to ineffective interventions can be read from Table 1 by viewing the row of a common practice element and connecting it to commonly used process elements in column four (e.g., delivered by caregiver), commonly used implementation elements in column five (e.g., quality monitoring), and to other practice elements in the last column that the common practice element were frequently combined with. The most common combination of elements in effective interventions minus in ineffective was *professionals training caregivers* in *parental school involvement at home* and *homework support* combined with *use of positive reinforcement*. In this combination, *organizational materials* were commonly used as intervention aids, *caregivers regularly received intervention support*, and the intervention was commonly implemented using *quality monitoring and educational material*. The second most common combination was similar in terms of process and implementation elements, but without *homework support* and with *psychoeducation* combined with *training in parental school involvement* and *positive reinforcement* instead. The third most common combination was *structured tutoring* combined with *training in parental school involvement at home* and *positive reinforcement*. When structured tutoring was included, the following process elements were more common: *feedback on performance*, *repeated training*, *direct instruction* as delivery method, *progressive difficulty* of tutoring, and use of *educational material.*

## Discussion

This review had three main aims: (1) to identify common practice, process, and implementation elements of OSTA interventions, (2) to review how often common elements and combinations of elements were used in effective studies subtracting how often it was used in ineffective or harmful studies, and (3) to identify common combinations of common practice, process, and implementation elements in effective interventions as opposed to in ineffective.

A total of 147 intervention elements were identified in included studies. Of these, 62 were practice elements and eight of these fulfilled criteria as common practice elements. We identified 49 process and 36 implementation elements used in combination with the common practice elements. Eleven unanticipated elements were discovered during coding, one of which turned out to be a common process element (direct instruction as delivery method). This speaks to the importance of allowing discovery of elements during the coding procedure. Using only a priori options increase the likelihood of confirmation bias (identifying expected elements only) and potentially significant elements might go undetected.

The three common practice elements with the highest FVs almost exclusively involved parents (training in parental school involvement at home, homework support, positive reinforcement). This is in line with prior research showing that parental involvement and support is important for children’s academic outcomes, especially in the form of positive expectations and home activities to improve learning (Wilder 2014). For instance, we found that training parents in how to engage themselves in their children’s academic experiences in combination with psychoeducation often was effective. While psychoeducation provides parents with an understanding of their role in their children’s education and why their involvement and expectations are important, training helps parents focus on activities that ameliorate their involvement and expectations appropriately. The results indicate that adding parent training elements in homework support and positive reinforcement can be beneficial as well.

A noteworthy finding is that all 11 interventions training parents in providing homework support to their children were effective. These findings appear to contradict prior studies. Wilder (2014) synthesized nine meta-analyses on parental involvement and concluded that homework support was the least effective element of parental involvement regardless of outcome measure. In the studies Wilder reviewed, homework support was mostly defined as parents helping their children directly with homework or checking homework. We defined homework support as a combination of the following three closely related discrete practice elements: Training and guidance in (1) how to appropriately support and instruct children during homework, (2) appropriate homework structure and routines, and (3) appropriate homework environments. Moreover, we defined checking homework as a separate discrete practice element. When these discrete practice elements appeared in effective interventions, they were always used in combination with other forms of parental involvement, such as academic learning activities at home or facilitating home-school collaboration. Using our definition, only the first discrete element is comparable with homework support reviewed by Wilder. We found no interventions delivering homework support only in the form of helping children with homework, which might explain the contradictive results. Similarly, checking homework had a frequency count of 6 in effective studies. However, checking homework was either combined with homework instruction, structure and routines, homework contracts, structured tutoring, or positive reinforcement when it was used in effective interventions. Wilder did report on meta-analyses that found positive results from interventions targeting homework routines and appropriate homework environment, offering additional explanation. One way of interpreting these results is that homework structure, routines, and environment may be of greater importance than direct homework assistance (or checking homework) by parents. Conversely, the effectiveness of homework support appears contingent upon it being coupled with training in other forms of parental involvement.

Interestingly, structured tutoring was the most common practice element, being used in 15 effective interventions. However, 25% of the studies using structured tutoring did not elicit statistically significant improvements. This demonstrates the added nuance of also reviewing elements in ineffective studies. Popular elements are not necessarily the most effective, and reviews of common elements should be mindful of popularity bias. Some elements can depend on other elements and characteristics for effectiveness. This review indicates that structured tutoring can be effective for reading skills; however, it appears more likely to be effective when it progresses in difficulty, includes reading aloud and receiving feedback, is repeated over time, and is combined with positive parental involvement.

The most frequently measured outcome was by far children’s reading abilities (21 studies), an important consideration when interpreting the results. The systematic search and selection did not favor studies measuring reading and so there appears to be a disproportionate high number of studies on OSTA interventions measuring reading skills compared with math skills, grade point average, or other academic skills. Reading difficulties might be viewed as particularly important compared with other academic difficulties because reading skills are necessary in most academic subjects. Another explanation could be that reading difficulties are more noticeable compared with problems with math or other academic skills. Nevertheless, there seems to be a gap in the literature about effective interventions for academic abilities other than reading skills.

### Implications and Recommendations for Research

The primary implication from the present review concerns common elements for helping children at risk improve reading abilities. The results also offer some support for common elements to improve math abilities and grade point average. In addition, the methodology applied in the review adds to existing common elements methodology and can inform future reviews of common intervention elements. Implications are threefold:

#### (a) Generation of Evidence-Informed Hypotheses

The methodology used in this review provides details about how and under what circumstances common practice elements are most frequently delivered, implemented, and combined in effective interventions accounted for in ineffective interventions. This can enable generation of hypotheses about how, when, in what forms, and for whom these common elements are likely to function. Experimentally testing these hypotheses could increase our understanding about mechanisms of change in OSTA interventions, and in turn inform research and practice. We identified four common practice elements used in interventions that were effective across all three outcomes (reading, math, and GPA). Identifying elements that are effective across multiple outcome domains should be prioritized in further studies in efforts to increase reach and utility of interventions. For instance, as shown in table 3 (available online) positive reinforcement, psychoeducation, and goal setting have been identified as common practice elements in several reviews of effective psychosocial interventions. An element’s contribution to effectiveness might be contingent upon other elements, factors, or structure (e.g., sequencing of elements). Future reviews should add structural elements such as sequencing, temporality, and dosage to coding of common elements, as they can likely improve hypotheses generation as well.

#### (b) Inform Design and Re-design of Interventions

The results of this review can be used to re-design OSTA interventions in efforts to optimize effectiveness and efficiency. For instance, elements with high FVs can be added as these likely contribute to favorable outcomes, and/or elements with low FVs can be removed as they might be superfluous. The results can also inform psychosocial interventions for children at risk looking to add elements of academic support, either as new core intervention elements or adaptations. In addition, common practice, process, and implementation elements can be tailored and assembled into new or alternative forms for practice, suitable for design approaches such as co-creation and user-centered design (Engell et al. 2018; Lyon and Koerner 2016).

#### (c) Inform Education and Practice

Many practice settings in need of quality improvement are unable to meet implementation demands for evidence-based practices. Some argue that in such circumstances, an appropriate course of action is to educate and train practitioners in common elements of effective interventions seeing as they likely contribute to positive outcomes, are less resource- and readiness-demanding, and may be perceived as more implementable (Hogue et al. 2017; Dorsey et al. 2016). Results from this review can inform choices about OSTA practices to implement and how to deliver and implement them to help children at risk academically. However, to counterbalance the lack of evidence of causal inferences from specific elements, the implementation and use of common elements should be accompanied by quality measurement and assurance.

#### Recommendations for Reporting

Several studies in this review were limited in their reporting of details. Common elements analyses would benefit from more details about practices, delivery methods, and contexts in intervention studies, either in articles, manuals, or appendices. Future intervention studies should also adopt current reporting standards for implementation strategies (e.g., Leeman et al. 2017). Data on dosage and fidelity (e.g., adherence, competence, and adaptations) of specific intervention elements could further improve analyses. Increased use of computer science (e.g., machine learning) to review and accumulate scientific literature (e.g., Michie et al. 2017) will enable the field to manage, interpret, and learn from extensive amounts of available data.

### Limitations

The literature search was completed in April 2016, which is already somewhat dated. However, to our awareness, there are no more updated reviews on OSTA interventions recently published or ongoing. To form an impression of how potentially missed studies after April 2016 would influence common elements results, an updated search and pragmatic review was conducted for studies published from April 2016 to November 2019 prior to publication. The first author screened 2091 abstracts and 33 full texts and found four new eligible studies. The studies were reviewed for practice, process, and implementation elements. One of the studies would not have had any influence on common elements results due to lack of details reported about the intervention. Three would have had some minor influence on certain frequency counts, without changing any implications from the results (see online resource 1 for elaboration). Changing the results based on the pragmatic update would not be appropriate because the review process did not fully replicate the original rigorous review and coding process. More details about the updated search and included and excluded studies are available in online resource 1.

We were unable to translate five non-English written studies and excluded them even though they could have been relevant. The average publication year was 1997, which raises questions about relevance given that educational support measures are subject to renewal and development. However, the review of intervention characteristics (Online Resource 1) demonstrates that many specific practices used in OSTA interventions withstand the test of time and remain relevant today (e.g., direct instruction tutoring). Several included studies were either non-randomized or did not specify randomization procedures and causal effects cannot be inferred. Some studies reported high attrition or inadequately addressed attrition which introduces risk of bias. Type of risk was not weighted in risk of bias assessment. Arguably, certain types of risks should be given more weight than others (e.g., blinding of participants in social interventions might be less important compared with random allocation). The same applies to weighting based on use of active or passive comparison conditions, which was not done in this review. Chances of significant differences between two active conditions are lower than comparing an intervention to nothing. Weighting based on risk of bias and comparison criteria could have influenced study inclusion and should be considered a priori by future reviews.

All six studies labeled as ineffective in the review had positive results but did not reach statistical significance. Thus, deducting a frequency count value based on an element’s inclusion in these studies is a conservative interpretation. In the absence of intervention manuals, the interventions were coded based on published articles, appendixes, evaluation reports, and doctoral theses. Limited descriptions of interventions influence the amount and precision of intervention details coded. We used broad criteria for inclusion of populations and coded for diversity in terms of gender, two age groups, and reason for being considered at risk. Further studies should consider more detailed coding of population characteristics such as more age categories and ethnicity to enable differentiation. Frequency counts and frequency count values represent a synthesis of published literature and are thus subject to publication bias. Future reviews of common elements should employ tools to assess risk of publication bias to inform interpretation of results (e.g., Page et al. 2018).

### Differences from Protocol (registry: 2016, CRD42016032887)

Several alterations of the original protocol have been made (Engell et al. 2016): Similarly to recent common elements reviews (e.g., van der Put et al. 2018), we combined a partial systematic review with common elements analyses in one article instead of two separate. Since a standard systematic review was not completed, risk ratios or standardized mean differences have not been calculated, and we have not conducted a random-effects meta-analysis, sensitivity analyses, explored heterogeneity in effects estimates, subgroup analyses, meta-regressions, or assessed publication bias.

## Electronic Supplementary Material

ESM 1 (DOCX 247 kb)

ESM 2 (DOCX 58.1 kb)

ESM 3 (DOC 64 kb)

ESM 4 (PDF 165 kb)

ESM 5 (PDF 102 kb)

ESM 6 (PDF 190 kb)

## Data Availability

Coding matrices can be provided upon request.
