# Supplementary material for: Common Elements of Practice, Process and Implementation in Out-of-School-Time Academic Interventions for At-risk Children: a Systematic Review
Source: Prev Sci. 2020 Feb 4;21(4):545–56. doi: 10.1007/s11121-020-01091-w (PMC7162823; doi:10.1007/s11121-020-01091-w)
Supplement: Supplementary file 1 — (DOCX 247 kb) [file 11121_2020_1091_MOESM1_ESM.docx]

Common Elements of Practice, Process and Implementation in Out-of-School-Time Academic Interventions for At-risk Children: a Systematic Review

Engell, T., Kirkøen, B., Hammerstrøm, K.T., Kornør, H., Ludvigsen, K.H., Hagen, K.A

*Prevention Science*

Corresponding author:

Thomas Engell, [te@r-bup.no](mailto:te@r-bup.no)

Regional Centre for Child and Adolescent Mental Health, Eastern and Southern Norway

# Supplementary file 1

Content

[Supplementary file 1 1](#_Toc31044785)

[1. Frequencies of process elements, implementation elements and other characteristics in effective OSTA interventions 2](#_Toc31044786)

[2. Coders, coding statistics and agreement 3](#_Toc31044787)

[3. Definitions of practice-, process-, and implementation elements and other intervention characteristics 3](#_Toc31044788)

[3. Search strategies 13](#_Toc31044789)

[4. Characteristics of included studies 16](#_Toc31044790)

[5. Risk of bias of included studies 51](#_Toc31044792)

[6. Reference list included studies 53](#_Toc31044793)

[7. Excluded studies 57](#_Toc31044794)

[8. Updated search from 2016 to November 2019 70](#_Toc31044795)

## Frequencies of process elements, implementation elements and other characteristics in effective OSTA interventions

Table 4 Frequencies of process elements, implementation elements, and other characteristics in effective OSTA interventions

| **Process elements (N=49)** | **Frequency** | **Implementation elements (N=36)** | **Frequency** |
| --- | --- | --- | --- |
| Regularly support | 25 | Quality monitoring | 18 |
| Use of educational material | 22 | Provide ongoing consultation | 17 |
| Delivered by professional | 22 | Distribute educational materials | 16 |
| Repeated training | 20 | Conduct educational meetings | 9 |
| Content received by parent/caregiver | 19 | Clinical supervision | 8 |
| Less than 3 hours a week, more 4 months | 18 | Conduct ongoing training | 7 |
| 1on1 caregiver interaction | 18 | Use train the trainer | 7 |
| Delivered by parent/caregiver | 18 | Involve end-users | 7 |
| Multi-element | 17 | Remind practitioners | 6 |
| 1on1 professional interaction | 16 | Centralized technical assistance | 6 |
| Delivered at home | 16 | Audit and feedback | 5 |
| Feedback on performance/supervision | 16 | Conduct outreach visits | 5 |
| External monitoring | 16 | Facilitate relay of clinical data | 5 |
| Individualized | 16 | Fund and contract for the innovation | 4 |
| Progressive difficulty | 15 | Training with feedback | 4 |
| Organizational material | 15 | Promote adaptability | 4 |
| Less than 3 hours a week, less than four months | 15 | Identify and prepare champions | 3 |
| Delivered using group instruction/lecture | 13 | Obtain formal commitments | 3 |
| Content received by K-3 with identified risk | 13 | Training using role-play | 3 |
| User involvement child | 13 | intervene with end-users to enhance uptake and adherence | 3 |
| User involvement parent | 12 | Revise professional roles | 2 |
| Content received by 4-7 with identified risk | 12 | Create learning collaborative | 2 |
| Content received by K-3 with indicated risk | 10 | Local technical assistance | 2 |
| Content received by 4-7 with indicated risk | 10 | Recruit, designate and train for leadership | 2 |
| Delivered by Paraprofessional | 10 | Conduct local consensus discussions | 2 |
|  |  | Use capitated payments | 2 |
| Delivered using group interaction | 10 | Use other payment schemes | 2 |
| Use of literary material | 10 | Alter incentive/allowance structures | 2 |
| Self-monitoring | 9 | Local needs assessment | 2 |
| Use of board games/material games | 9 | use advisory boards and workgroups  build coalition | 1 |
| Phonemics based | 8 | clinical implementation team meetings | 1 |
| Delivered using handouts/material | 8 | tailor strategies | 1 |
| Delivered at school | 8 | Purposefully reexamine implementation | 1 |
| Delivered at after-school program/center | 7 | Facilitation | 1 |
| Delivered by volunteer/amateur | 7 | Obtain and use end-user feedback | 1 |
| Flexible/adaptive | 7 | Formal implementation blueprint/plan | 1 |
| Delivered by computer | 6 | **Other characteristics** | **Frequency** |
| Delivered using computer program | 5 | Outcomes measured shortly after intervention | 24 |
| Use of informational material | 5 | Outcomes measured 2-12 months after intervention | 8 |
| 1on1 interaction with volunteer/amateur | 4 | Outcomes measured 12+ months after intervention | 4 |
| Use of audio | 4 | Theoretical orientation: behavioral | 7 |
| Culturally sensitive | 4 | Theoretical orientation: social learning | 3 |
| Use of video/tv | 3 | Theoretical orientation: ecological systems | 2 |
| 3 hours a week or more, less than four months | 3 | Theoretical orientation: transactional | 1 |
| Delivered using role play | 3 | Theoretical orientation: proximal orientation | 1 |
| 1on1 interaction with paraprofessional | 3 | Theoretical orientation: positive psychology | 1 |
| 1on1 instruction | 2 | Theoretical orientation: attachment theory | 1 |
| Observation and instruction | 2 | Theoretical orientation: efficacy theory | 1 |
| Delivered at service agency | 2 | Theoretical orientation: fantasy realization | 1 |
| Use of web-based/application game | 2 | Theoretical orientation: psychodynamic | 1 |
| Support on demand | 2 | Theoretical orientation: sociocognitive | 1 |
| Delivered by peer | 1 | Theoretical orientation: Adlerian | 1 |

## Coders, coding statistics and agreement

Coders were either extensively experienced (HK, PhD), experienced (KHL; TE), or familiar (EN; BK, PhD) with systematic reviews and intervention literature.

| **Statistic** | **Counts** | **Agreement** | **Coders** |
| --- | --- | --- | --- |
| Mean number of coding inputs per intervention | 170.70 (*SD=97*.50) |  | BK, HK, KHL, EN, TE |
| Total coding agreement all interventions | 4.972 total inputs | **90.4%** | BK, HK, KHL, EN, TE |
| Coding agreement pair 1 | 638 inputs, 91 conflicts, | **85.74%** | HK, TE |
| Coding agreement pair 2 | 2218 inputs, 242 conflicts, 6 added elements with conflicts, | **89.09%** | KHL, TE |
| Coding agreement pair 3 | 821 inputs, 59 conflicts, 1 added element with conflict | **92.81%** | EN, TE |
| Coding agreement pair 4 | 1295 inputs, 90 conflicts, 4 added elements without conflicts | **93.05%** | BK, TE |

BK= Benedikte Kirkøen, PhD. HK= Hege Kornør, PhD. KHL= Kristine H. Ludvigsen, MsC. EN= Erik Nakken, PhD student. TE= Thomas Engell, PhD student.

## Definitions of practice-, process-, and implementation elements and other intervention characteristics

| **Categories, elements and characteristics** | **Definitions and descriptions** |
| --- | --- |
| 1. **Practice elements** | Specific activities or actions used in an intervention to evoke or influence an outcome. Also known as content elements and specific factors |
| **Structured Tutoring** | Tutoring that is structured based on a curriculum or more or less a stringent procedure of providing tutoring. Components below in cursive overlap with process elements |
| *Direct/explicit instruction:* | Providing instruction in a direct manner to an audience. Often in the form of lecturing or demonstration. Also referred to as "passive learning" or "explicit instruction". |
| *Active learning:* | learning in which individuals are actively or experientially involved in the learning process, such as solving puzzles based on instruction from a teacher. |
| *Interactional learning* | Interactional learning: learning in which the learning process is highly based on interactions amongst individuals, such as cooperations and discussions. |
| *Trial and correction:* | Trial and correction: Refers do different methods combining instruction with active learning, performance feedback and correction in iterative trials. |
|  |  |
| **Unstructured Tutoring** | Tutoring that does not follow a specific curriculum or procedure. Components below in cursive overlap with process elements |
| *Direct/explicit instruction:* | Providing instruction in a direct manner to an audience. Often in the form of lecturing or demonstration. Also referred to as "passive learning" or "explicit instruction". |
| *Active learning:* | learning in which individuals are actively or experientially involved in the learning process, such as solving puzzles based on instruction from a teacher. |
| *Interactional learning:* | Learning in which the learning process is highly based on interactions amongst individuals, such as cooperations and discussions. |
| *Trial and correction:* | Refers do different methods combining instruction with active learning, performance feedback and correction in iterative trials. |
|  |  |
| **Activities, tools and techniques** |  |
| Observational learning: | Learning by observing behavior or performance |
| Modeling: | Modeling refers to a type of observational learning where individuals learn by observing the behavior or performance of others and then imitate |
| Role-playing: | Refers to learning by assuming the role of someone or something else and consciously or unconsciously learning by adapting to the role. |
| Computerized/web-based reading program | Any form computer program or program via internet on a computer intended to improve reading abilities. |
| Playing reading game: Playing | Any form of game intended to improve reading abilities. |
| Playing math game: | Playing any form of game intended to improve mathematical abilities. |
| Playing other educational game: | Playing a game intended to improve any other academic ability other than reading and math. |
| Solving math paper: | Solving mathematical tasks on paper |
| Solving math digitally: | Solving mathematical tasks digitally |
| Use of homework contracts: | Using written or oral contracts and agreements to commit children to doing homework. |
| Use of behavioral contracts: | Using written or oral contracts and agreements to commit children to exercise appropriate behavior and/or avoid inappropriate behavior. |
| Check/review homework: | Checking homework completion and/or performance on a consistent basis |
| Monitor performance: | Some form of repetitive audit of performance over time. Could be results from repetitive tests or use of performance indicators |
| Information: | Any form of information given not applicable to other elements |
| Praise: | Explicitly stating that giving praise is a part of the intervention |
| Feedback: | Explicitly stating that giving feedback is a part of the intervention |
| Correction: | Explicitly stating that giving corrections are a part of the intervention |
| Use of positive reinforcement: | The use of immediate positive responses to welcomed actions, behaviors or performances |
| Use of negative reinforcement: | The use of immediate negative responses to unwelcomed actions, behaviors or performances |
| Use of incentives: | Explicitly stating that use of rewards or other incentives are a part of the intervention. Such as point systems or rewarding privileges for good performance. Giving praise or positive remarks for achievement are not incentives, but positive reinforcement |
| Other types of behavioral modification efforts: | The use of various efforts intended to modify behavior, not applicable to other elements. |
| Psychoeducation: | Any form of empowerment and/or educating of the affected using "condition-specific" information. E.g. telling parents about research on the importance of parental involvement or telling kids who struggle with reading that having trouble learning to read is normal and can be overcome with practice |
| Problem solving: | An activity that includes being aware of a problem or challenge, and suggesting or carrying out solutions to overcome the problem. Solving math problems should be coded as a "solving math"-element |
| Attitudinal/perceptional modification efforts: | The use of any specific efforts intended to modify attitudes or perceptions. Can be double coded with psychoeducation. |
| Motivational modification efforts: | Motivational modification efforts: The use of any specific efforts intended to increase motivation. Can be double coded with psychoeducation |
| Social Skills training: | Training or guidance with the intention to improve the ability to interact and communicate, verbally or non-verbally |
| Emotion regulation training: | Training or guidance with the intention to improve the ability to respond emotionally appropriate to experiences |
| Working memory training: | Training or guidance with the intention to improve the ability to cognitively hold, process and manipulate information |
| Updating training: | Training or guidance with the intention to improve the ability to respond to changes in the environment in a flexible and adaptive manner |
| Paired reading: | A one to one reading technique where one individual reads aloud and the other provides correction and feedback. Is usually, but not exclusively, complimented with both individuals reading together as well |
| Reading alone: | An individual read alone undisturbed, aloud or silent |
| Reading aloud to someone: | An individual read aloud to someone |
| Word recognition: | Training in the ability to recognize written words correctly and effortlessly |
| Word decoding: | Training in the ability to recognize letter-sound relationships, and application of letter-sound knowledge to create and pronounce words correctly |
| Observation of learning/interaction: | The use of observation of others learning and interacting as a learning technique |
| Transactions/dialogs: | Transactions/dialogs: the use of two-way communication between individuals as an explicit learning technique. |
|  |  |
| **Guidance/training in:** | The following elements are combinations of the process element "providing guidance or training" and specific practice elements. Coding one or more of these elements does not exclude coding of applicable elements above. E.g. A homework support intervention providing training on facilitating homework discipline through the use of checking homework on a consistent basis and providing feedback should be coded both as "homework support: structure/discipline", "check/review homework", and "feedback" |
| Homework support: structure/discipline: | Guidance in appropriate structure and discipline regarding school work in the home. |
| Homework support: instruction: | Guidance in how to appropriately instruct and support homework |
| Homework support: work environment: | Guidance in facilitating an appropriate environment for school work in the home |
| Homework support: not specified: | Guidance in other types of homework support not specified above |
| Parental involvement: at home: | Guidance in any form of parental involvement supporting the child's education at home |
| Parental involvement: at school: | Guidance in any form of parental involvement supporting the child's education at school. Such as parent-teacher meetings, volunteer work at school-dance etc.. |
| Home-school collaboration/communication: | Guidance in any form of positive collaboration or communication between caregivers at home and teachers or other school personnel at school. |
|  |  |
| 1. **Process elements and intervention characteristics** | Process elements are how and under what circumstances practice elements are delivered. Together with characteristics of interventions, process elements describe or categorize how, when, where, why, for whom, and by whom practice elements were delivered. Characteristics also include information on outcome measurement |
| **Outcomes** |  |
| Reading abilities/grades: | Any measure of reading abilities or performance |
| Math abilities/grades: | Any measure of mathmatical abilities or performance |
| Other academic abilities/grades: | Any measure of academic abilities or performanceother than in reading or mathematics |
| GPA: | Grade point average |
| Parental engagement: | Any measure of parental engagement or involvement |
| **Time of outcome measure** |  |
| Short term: | Within two months after intervention |
| Mid term: | Two to 12 months after intervention |
| Long term: | more then 12 months after intervention |
|  | **Outcome measured on:** |
| K-3 indicated: | Children k-3. grade with indicated risk of academic difficulties: children from families of low socioeconomic status, children in child protection, foster children |
| 4-7 indicated: | Children in 4.-7. grade with indicated risk of academic difficulties: children from families of low socioeconomic status, children in child protection, foster children |
| K-3 identified: | Children k-3. grade identified as low academic achievers compared to peers based on grades or low scores on tests of academic abilities, children identified with learning disabilities or cognitive deficiencies |
| 4-7 identified: | Children in 4.-7. grade identified as low academic achievers compared to peers based on grades or low scores on tests of academic abilities, children identified with learning disabilities or cognitive deficiencies |
| Parent/caregiver: | Parent, foster parent, or other primary caregiver |
| **Content received by** |  |
| K-3 indicated: | Children k-3. grade with indicated risk of academic difficulties: children from families of low socioeconomic status, children in child protection, foster children |
| 4-7 indicated: | children in 4.-7. grade with indicated risk of academic difficulties: children from families of low socioeconomic status, children in child protection, foster children |
| K-3 identified: | Children k-3. grade identified as low academic achievers compared to peers based on grades or low scores on tests of academic abilities, children identified with learning disabilities or cognitive deficiencies |
| 4-7 identified: | Children in 4.-7. grade identified as low academic achievers compared to peers based on grades or low scores on tests of academic abilities, children identified with learning disabilities or cognitive deficiencies |
| Parent/caregiver: | Parent, foster parent, or other primary caregiver |
| **Delivered by** |  |
| By parent/primary caretaker: | Parent, foster parent, or other primary caregiver |
| By volunteer/amateur: | No relevant education |
| By paraprofessional: | 1-3 years relevant education |
| By professional: | 4 or more years relevant education |
| By peer: | Peer tutor, classmate, friend or other peers |
| By computer |  |
| By application on phone/tablet |  |
| By television |  |
| By audio |  |
|  |  |
| **Delivery method:** | **Delivery method:** |
| 1on1 parent interaction: | Subject interacting with parent or other caregiver |
| 1on1 peer interaction: | Subject interacting with peer |
| 1on1 Volunteer/amateur interaction: | Subject interacting with volunteer/amateur |
| 1on1 paraprofessional interaction: | Subject interacting with paraprofessional |
| 1on1 professional interaction: | Subject interacting with professional |
| Group interaction: | Subject interacting in a group |
| 1on1 instruction: | Subject being instructed one to one |
| Group instruction: | Subject being instructed in a group |
| Child/parent together: | Child and primary caregiver interacting together |
|  |  |
| **Intensity and duration:** | **Intensity and duration:** |
| Limited and short: | Less than six hours a week, less than four months |
| Limited over time: | Less than six hours a week, four months or longer |
| Intensive and short: | Six hours a week or more, less than four months |
| Intensive over time: | Six hours a week or more, four months or longer |
|  |  |
| **Context of delivery:** | **Context of delivery:** |
| Home |  |
| Outside |  |
| At school: | Intervention elements happening at school, regardless of time of day |
| At service agency: | Intervention elements happening at child welfare service, mental health service, special educational service etc. |
| At residential facilities: | Intervention elements happening at residential care facilities |
| At after-school program/center: | Intervention elements happening at a center or facility specifically aimed at delivering after school activities |
|  |  |
| **Materials and games:** |  |
| Informational material: | Any material containing information relevant for one or more intervention elements |
| Educational material: | Material with primary aim to educate in one or more academic subjects (Educational books, workbooks in math, educational dvds etc.) |
| Literary material: | Story telling books, poems and other literature without a primary aim to educate |
| Organizational material: | Homework plans, family-planners, weekly-plans, checklists, homework contracts, behavioral contracts etc. |
| Board/material games: | Any non-digital game requiring any form of material |
| Web-based/PC/App game: | Any games requiring internet on any digital platform (PC, tablet, mobile etc.) |
| Video game: | Games played on Xbox, PlayStation, Nintendo or other consoles |
| Computer programs: | Any program not considered a game requiring a computer |
|  |  |
| **Intervention support:** |  |
| Element being repeated: | Training/instruction/activity being repeated at least four times |
| Support on demand: | The subject of the intervention has the opportunity to request support if necessary |
| Feedback on performance: | Subject recieves feedback on performance |
| Self monitoring: | The subject monitors (logging, registering, checking) own performance on intervention elements, or usage/dosage of intervention elements |
| External monitoring: | Intervention deliverer, researchers, teachers, peers or others monitor (logging, registering, checking) the subjects performance on intervention elements, or usage/dosage of intervention elements |
| Regularly support: | The subject receives some sort of intervention relevant support multiple times during the intervention period from an intervention deliverer without having to request it (e.g. training sessions over time, booster sessions, follow up calls etc. |
| **User involvement and influence** |  |
| Child influence: | Explicitly stating that personal opinions or preferences from the child participating in the intervention influenced the delivery of the intervention |
| Parent/caregiver influence: | Explicitly stating that personal opinions or preferences from the parent/caregiver participating in the intervention influenced the delivery of the intervention |
| Practitioner influence: | Explicitly stating that personal opinions or preferences from intervention deliverer influenced the delivery of the intervention, including authorised adaptations of manuals or standards and unwanted drift from manual or standards |
|  |  |
| **Theoretical orientation** | Theory the intervention is based on. Several might overlap. Not code if not explicitly stated |
| Behavioral | Any behavioral theory |
| Transactional | Transactional theory |
| Social learning | Social learning theory |
| Ecological systems | Ecological systems theory |
| Psychodynamic | Psychodynamic theory |
| Proximal development | Theory of proximal development |
| Role-model theory | Role-model theory |
| Efficacy theory | Efficacy theory |
| Sociocognitive | Sociocognitve mechanism for succesfull learning with technology |
| **Other characteristics** | Other characteristics not applicable to existing categories |
| Culturally sensitive: | Clear indications is provided that the intervention is culturally sensitive. E.g. Training deliverers in relevant cultural sensitivity, using trained translators, doing adaptations of the intervention to fit minority groups etc. |
| Multi-element: | The intervention consists of three or more different practice elements |
|  |  |
| **Implementation elements** | Elements of strategies used to implement practice- and process elements. These elements are based on implementation strategies compiled by the Expert Recommendations for Implementing Change (ERIC) project (Powell et al., 2015). Due to large variation in training techniques coded as the implementation strategy *make training dynamic,* coders (TE and BK) decided to disentangle the element into more discrete elements by dividing it into *role play in training*, *interactive training*, *training with feedback, training with multiple training techniques,* and *other dynamic training techniques..* |
| **Use evaluative and iterative strategies** |  |
| Readiness assessment | Assess various aspects of an organization to determine its degree of readiness to implement, barriers that may impede implementation, and strengths that can be used in the implementation effort. |
| Audit and feedback | Collect and summarize clinical performance data over a specified time period and give it to clinicians and administrators to monitor, evaluate, and modify provider behavior. |
| Purposefully reexamine implementation | Monitor progress and adjust clinical practices and implementation strategies to continuously improve the quality of care. |
| Quality monitoring | Develop and organize systems and procedures that monitor clinical processes and/or outcomes for the purpose of quality assurance and improvement. |
| Formal implementation blueprint/plan | Develop a formal implementation blueprint that includes all goals and strategies. The blueprint should include the following: 1) aim/purpose of the implementation; 2) scope of the change (e.g., what organizational units are affected); 3) timeframe and milestones; and 4) appropriate performance/progress measures. Use and update this plan to guide the implementation effort over time. |
| Local needs assessment | Collect and analyze data related to the need for the innovation. |
| Stage scale up | Phase implementation efforts by starting with small pilots or demonstration projects and gradually move to a system wide rollout. |
| Obtain and use end-user feedback | Develop strategies to increase patient/consumer and family feedback on the implementation effort. |
| Use competence benchmark (new) | Select or recruit providers based on some form of competence assessment. |
| Cyclical test of change | Implement changes in a cyclical fashion using small tests of change before taking changes system-wide. Tests of change benefit from systematic measurement, and results of the tests of change are studied for insights on how to do better. This process continues serially over time, and refinement is added with each cycle. |
| **Provide interactive assistance** |  |
| Facilitation | A process of interactive problem solving and support that occurs in a context of a recognized need for improvement and a supportive interpersonal relationship. |
| Local technical assistance | Develop and use a system to deliver technical assistance focused on implementation issues using local personnel. |
| clinical supervision | Provide clinicians with ongoing supervision focusing on the innovation. Provide training for clinical supervisors who will supervise clinicians who provide the innovation. |
| centralized technical assistance | Develop and use a centralized system to deliver technical assistance focused on implementation issues. |
| **Adapt and tailor to context** |  |
| tailor strategies | Tailor the implementation strategies to address barriers and leverage facilitators. that were identified through earlier data collection |
| Promote adaptability | Identify the ways a clinical innovation can be tailored to meet local needs and clarify which elements of the innovation must be maintained to preserve fidelity. |
| Use data experts | Involve, hire, and/or consult experts to inform management on the use of data generated by implementation efforts. |
| Use data warehouseing techniques | Integrate clinical records across facilities and organizations to facilitate implementation across systems. |
| **Develop stakeholder interrelationships** |  |
| identify and prepare champions | Identify and prepare individuals who dedicate themselves to supporting, marketing, and driving through an implementation, overcoming indifference or resistance that the intervention may provoke in an organization. |
| clinical implementation team meetings | Develop and support teams of clinicians who are implementing the innovation and give them protected time to reflect on the implementation effort, share lessons learned, and support one another’s learning. |
| recruit, deisgnate and train for leadership | Recruit, designate, and train leaders for the change effort. |
| inform local opinion leaders | Inform providers identified by colleagues as opinion leaders or “educationally influential” about the clinical innovation in the hopes that they will influence colleagues to adopt it. |
| build coalition | Recruit and cultivate relationships with partners in the implementation effort. |
| obtain formal commitments | Obtain written commitments from key partners that state what they will do to implement the innovation. |
| identify early adopters | Identify early adopters at the local site to learn from their experiences with the practice innovation. |
| conduct local consensus discussions | Include local providers and other stakeholders in discussions that address whether the chosen problem is important and whether the clinical innovation to address it is appropriate. |
| capture and share knowledge | Capture local knowledge from implementation sites on how implementers and clinicians made something work in their setting and then share it with other sites. |
| use advisory boards and workgroups | Create and engage a formal group of multiple kinds of stakeholders to provide input and advice on implementation efforts and to elicit recommendations for improvements. |
| use implementation advisor | Seek guidance from experts in implementation. |
| model and simulate change | Model or simulate the change that will be implemented prior to implementation. |
| vist other sites | Visit sites where a similar implementation effort has been considered successful. |
| involve executive boards | Involve existing governing structures (e.g., boards of directors, medical staff boards of governance) in the implementation effort, including the review of data on implementation processes. |
| develop and implementation glossary | Develop and distribute a list of terms describing the innovation, implementation, and stakeholders in the organizational change. |
| develop academic partnerships | Partner with a university or academic unit for the purposes of shared training and. |
| promote network weaving | bringing research skills to an implementation project. |
| **Train and educate stakeholders** |  |
| conduct ongoing training | Plan for and conduct training in the clinical innovation in an ongoing way |
| provide ongoing consultation | Provide ongoing consultation with one or more experts in the strategies used to support implementing the innovation. |
| develop educational materials | Develop and format manuals, toolkits, and other supporting materials in ways that make it easier for stakeholders to learn about the innovation and for clinicians to learn how to deliver the clinical innovation. |
| make training dynamic (further disentaglement below) | Vary the information delivery methods to cater to different learning styles and work contexts, and shape the training in the innovation to be interactive. |
| - *Training using role play* | Actively engaging trainees in changing their behavior to act out a role for the purpose of learning |
| - *Interactive training* | Learning occurs through mutual actions between trainers and trainees. In interactive training, teaching and learning are interrelated, and trainees learn through a process of reflection through personal experience. |
| - *Training with feedback* | Any form of training with real time feedback on performance or behavior from a trainer or peer |
| - *Training with multiple training techniques* | Any form of training where two or more defined training techniques are combined |
| - *Other dynamic training techniques* | Any form of dynamic training not applicable to the training strategies above |
| distrubute educational materials | Distribute educational materials (including guidelines, manuals, and toolkits) in person, by mail, and/or electronically. |
| use train-the-trainer | Train designated clinicians or organizations to train others in the clinical innovation. |
| conduct educational meetings | Hold meetings targeted toward different stakeholder groups (e.g., providers, administrators, other organizational stakeholders, and community, patient/consumer, and family stakeholders) to teach them about the clinical innovation. |
| conduct outreach visits | Have a trained person meet with providers in their practice settings to educate providers about the clinical innovation with the intent of changing the provider’s practice. |
| create learning collaborative | Facilitate the formation of groups of providers or provider organizations and foster a collaborative learning environment to improve implementation of the clinical innovation. |
| shadow other experts | Provide ways for key individuals to directly observe experienced people engage with or use the targeted practice change/innovation. |
| work with educational institutions | Encourage educational institutions to train clinicians in the innovation. |
| Training with feedback | Training with real time feedback from trainer. |
| Training using role play | Use of role play during training of stakeholders/providers are explicitly stated. |
| Not specificed training | Any other training effort not that does not fit any of the other elements. |
| **Support clinicians/practitioners** |  |
| Facilitate relay of clinical data | Provide as close to real-time data as possible about key measures of process/outcomes using integrated modes/channels of communication in a way that promotes use of the targeted innovation. |
| remind practitioners | Develop reminder systems designed to help clinicians/practitioners to recall information and/or prompt them to use the clinical innovation. |
| develop resource sharing agreements | Develop partnerships with organizations that have resources needed to implement the innovation. |
| revise professional roles | Shift and revise roles among professionals who provide care, and redesign job characteristics |
| create new clinical teams | Change who serves on the clinical team, adding different disciplines and different skills to make it more likely that the clinical innovation is delivered (or is more successfully delivered). |
| **Engage end-users/consumers** |  |
| Involve end-users | Engage or include patients/consumers and families in the implementation effort. |
| intervene with end-users to enhance uptake and adherence | Develop strategies with patients to encourage and problem solve around adherence. |
| prepare end-users to be active participatns | Prepare patients/consumers to be active in their care, to ask questions, and specifically to inquire about care guidelines, the evidence behind clinical decisions, or about available evidence-supported treatments. |
| increase demand | Attempt to influence the market for the clinical innovation to increase competition intensity and to increase the maturity of the market for the clinical innovation. |
| use mass media | Use media to reach large numbers of people to spread the word about the clinical innovation. |
| **Utilize financial strategies** |  |
| fund and contract for the inovation | Governments and other payers of services issue requests for proposals to deliver the innovation, use contracting processes to motivate providers to deliver the clinical innovation, and develop new funding formulas that make it more likely that providers will deliver the innovation. |
| access new funding | Access new or existing money to facilitate the implementation. |
| place innovation on fee | Work to place the clinical innovation on lists of actions for which providers can be reimbursed (e.g., a drug is placed on a formulary, a procedure is now reimbursable). |
| alter incentive/allowance structures | Work to incentivize the adoption and implementation of the clinical innovation. |
| make billing easier | Make it easier to bill for the clinical innovation. |
| alter end-user fees | Create fee structures where patients/consumers pay less for preferred treatments (the clinical innovation) and more for less-preferred treatments. |
| use other payment schemes | Introduce payment approaches (in a catch-all category). |
|  |  |
| use capitated payments | Pay providers or care systems a set amount per patient/consumer for delivering clinical care. |
| **Change infrastructure** |  |
| mandate change/innovation | Have leadership declare the priority of the innovation and their determination to have it implemented. |
| change record system | Change records systems to allow better assessment of implementation or clinical outcomes. |
| change physical structure and equiptment | Evaluate current configurations and adapt, as needed, the physical structure and/or equipment (e.g., changing the layout of a room, adding equipment) to bestaccommodate the targeted innovation |
| create or change credentialing/licensure | Create an organization that certifies clinicians in the innovation or encourage an existing organization to do so. Change governmental professional certification or licensure requirements to include delivering the innovation. Work to alter continuing education requirements to shape professional practice toward the innovation. |
| Change service sites | Change the location of clinical service sites to increase access. |
| Change accreditation or membership requirements | Strive to alter accreditation standards so that they require or encourage use of the clinical innovation. Work to alter membership organization requirements so that those who want to affiliate with the organization are encouraged or required to use the clinical innovation. |
| start a dissemination organization | Identify or start a separate organization that is responsible for disseminating the clinical innovation. It could be a for-profit or non-profit organization. |
| change liability laws | Participate in liability reform efforts that make clinicians more willing to deliver the clinical innovation. |

## Search strategies

All searches performed Januar 4, 2016 by research librarians Sølvi Biedilæ and Brynhildur Axelsdottir

**ERIC 1965 to September 2015**4316 hits

| **#** | **Searches** | **Results** |
| --- | --- | --- |
| 1 | academic support services/ or remedial programs/ or weekend programs/ or nonschool educational programs/ or home programs/ or family programs/ or compensatory education/ or supplementary education/ or exp tutoring/ or exp remedial instruction/ or tutors/ or educational games/ | 33631 |
| 2 | ((academic* or educational* or remedial* or home* or out-of-school* or non-school* or family or parent* or caregiver* or liaison*) adj2 (program* or support* or intervention* or training* or game* or app or apps)).ti,ab. | 49470 |
| 3 | ((compensatory or supplementary or extra-curricular) adj2 education*).ti,ab. | 1935 |
| 4 | (tutoring or tutors).ti,ab. | 10121 |
| 5 | 1 or 2 or 3 or 4 | 82402 |
| 6 | exp academic achievement/ or exp mathematics achievement/ or exp reading achievement/ or exp science achievement/ or exp writing achievement/ or exp academic ability/ or exp achievement gains/ or exp educational improvement/ or exp reading improvement/ or exp student improvement/ or exp writing improvement/ or exp academic failure/ or achievement gap/ or student improvement/ or dropouts/ or potential dropouts/ | 144744 |
| 7 | ((academic* or educational* or reading or math* or writing or student*) adj2 (improvement* or achievement* or ability or gain* or success* or performance*)).ti,ab. | 93757 |
| 8 | (prevent* adj2 (failure* or dropout* or gap*)).ti,ab. | 1531 |
| 9 | (improve* adj5 (grade* or score*)).ti,ab. | 3577 |
| 10 | 6 or 7 or 8 or 9 | 187917 |
| 11 | 5 and 10 | 18525 |
| 12 | program effectiveness/ | 48771 |
| 13 | (trial or random* or ((control* or comparison* or intervention*) adj5 (group* or school* or student*)) or cohort* or longitudinal or prospective or retrospective).ti,ab. | 107021 |
| 14 | 12 or 13 | 146548 |
| 15 | 11 and 14 | 5326 |
| 16 | (exp colleges/ or exp college students/) not (elementary education/ or elementary school students/ or elementary schools/ or grade 1/ or grade 2/ or grade 3/ or grade 4/ or grade 5/ or grade 6/ or grade 7/ or grade 8/ or exp children/ or exp early adolescents/) | 192228 |
| 17 | 15 not 16 | 4316 |

**Ovid MEDLINE(R) In-Process & Other Non-Indexed Citations and Ovid MEDLINE(R)** 1946 to Present
1120 hits

| **#** | **Searches** | **Results** |
| --- | --- | --- |
| 1 | Remedial Teaching/ or Computer-Assisted Instruction/ | 11486 |
| 2 | ((academic* or educational* or remedial* or home* or out-of-school* or non-school* or family or parent* or caregiver* or liaison*) adj2 (program* or support* or intervention* or training* or game* or app or apps)).ti,ab. | 58754 |
| 3 | ((compensatory or supplementary or extra-curricular) adj2 education*).ti,ab. | 80 |
| 4 | (tutoring or tutors).ti,ab. | 2217 |
| 5 | 1 or 2 or 3 or 4 | 71720 |
| 6 | Achievement/ or Student Dropouts/ or Underachievement/ | 15125 |
| 7 | ((academic* or educational* or reading or math* or writing or student*) adj2 (improvement* or achievement* or ability or gain* or success* or performance*)).ti,ab. | 19169 |
| 8 | (prevent* adj2 (failure* or dropout* or gap*)).ti,ab. | 2925 |
| 9 | (improve* adj5 (grade* or score*)).ti,ab. | 35901 |
| 10 | 6 or 7 or 8 or 9 | 69771 |
| 11 | 5 and 10 | 2867 |
| 12 | (clinical trial or comparative study or controlled clinical trial or evaluation studies or multicenter study or observational study or pragmatic clinical trial or randomized controlled trial).pt. | 2561048 |
| 13 | exp Therapeutics/ | 3637660 |
| 14 | exp clinical trial/ | 867520 |
| 15 | cohort studies/ or longitudinal studies/ or follow-up studies/ or prospective studies/ or retrospective studies/ | 1531645 |
| 16 | (trial or random* or ((control* or comparison* or intervention*) adj5 (group* or school* or student*)) or cohort* or longitudinal or prospective or retrospective).ti,ab. | 2278828 |
| 17 | 12 or 13 or 14 or 15 or 16 | 7142035 |
| 18 | 11 and 17 | 1712 |
| 19 | education, predental/ or education, premedical/ or exp education, professional/ or exp students, health occupations/ | 278272 |
| 20 | 18 not 19 | 1120 |

**PsycINFO** 1806 to December Week 5 2015
2988 hits

| **#** | **Searches** | **Results** |
| --- | --- | --- |
| 1 | remedial education/ or remedial reading/ or compensatory education/ or tutoring/ or computer assisted instruction/ | 17761 |
| 2 | ((academic* or educational* or remedial* or home* or out-of-school* or non-school* or family or parent* or caregiver* or liaison*) adj2 (program* or support* or intervention* or training* or game* or app or apps)).ti,ab. | 57012 |
| 3 | ((compensatory or supplementary or extra-curricular) adj2 education*).ti,ab. | 354 |
| 4 | (tutoring or tutors).ti,ab. | 5247 |
| 5 | 1 or 2 or 3 or 4 | 77456 |
| 6 | academic achievement/ or mathematics achievement/ or reading achievement/ or science achievement/ or academic failure/ or dropouts/ or potential dropouts/ or school dropouts/ | 61581 |
| 7 | ((academic* or educational* or reading or math* or writing or student*) adj2 (improvement* or achievement* or ability or gain* or success* or performance*)).ti,ab. | 70735 |
| 8 | (prevent* adj2 (failure* or dropout* or gap*)).ti,ab. | 678 |
| 9 | (improve* adj5 (grade* or score*)).ti,ab. | 8577 |
| 10 | 6 or 7 or 8 or 9 | 107658 |
| 11 | 5 and 10 | 8701 |
| 12 | limit 11 to "therapy (best balance of sensitivity and specificity)" | 1344 |
| 13 | treatment outcome clinical trial.md. | 32065 |
| 14 | (trial or random* or ((control* or comparison* or intervention*) adj5 (group* or school* or student*)) or cohort* or longitudinal or prospective or retrospective).ti,ab. | 456081 |
| 15 | exp treatment/ | 640150 |
| 16 | 13 or 14 or 15 | 1001019 |
| 17 | 11 and 16 | 2757 |
| 18 | 12 or 17 | 2988 |

**Cochrane Library**518 hits

ID Search Hits

#1 MeSH descriptor: [Remedial Teaching] explode all trees 119

#2 MeSH descriptor: [Computer-Assisted Instruction] explode all trees 940

#3 ((academic* or educational* or remedial* or home* or out-of-school* or non-school* or family or parent* or caregiver* or liaison*) near/2 (program* or support* or intervention* or training* or game* or app or apps)) 11731

#4 ((compensatory or supplementary or extra-curricular) near/2 education*) 16

#5 tutoring or tutors 181

#6 #1 or #2 or #3 or #4 or #5 12794

#7 MeSH descriptor: [Achievement] explode all trees 382

#8 MeSH descriptor: [Underachievement] explode all trees 13

#9 MeSH descriptor: [Student Dropouts] explode all trees 26

#10 ((academic* or educational* or reading or math* or writing or student*) near/2 (improvement* or achievement* or ability or gain* or success* or performance*)) 1898

#11 (prevent* near/2 (failure* or dropout* or gap*)) 899

#12 (improve* near/5 (grade* or score*)) 13110

#13 #7 or #8 or #9 or #10 or #11 or #12 16054

#14 #6 and #13 in Other Reviews and Trials 648

#15 MeSH descriptor: [Education, Professional] explode all trees 3290

#16 MeSH descriptor: [Education, Predental] explode all trees 0

#17 MeSH descriptor: [Education, Premedical] explode all trees 3

#18 MeSH descriptor: [Students, Health Occupations] explode all trees 926

#19 #15 or #16 or #17 or #18 3582

#20 #14 not #19 518

**PubMed (for references not included in Medline)**

820 hits

(((academic*[tiab] OR educational*[tiab] OR remedial*[tiab] OR home[tiab] OR "out-of-school*"[tiab] OR "non-school*"[tiab] OR family[tiab] OR parent*[tiab] OR caregiver*[tiab] OR liaison*[tiab]) AND (program*[tiab] OR support*[tiab] OR intervention*[tiab] OR training*[tiab] OR game*[tiab] OR app[tiab] OR apps[tiab])) OR ((compensatory[tiab] OR supplementary[tiab] OR "extra-curricular"[tiab]) AND education*[tiab]) OR tutoring[tiab] OR tutors[tiab]) AND (((academic*[tiab] OR educational*[tiab] OR reading[tiab] OR math[tiab] OR maths[tiab] OR mathematics[tiab] OR writing[tiab] OR student*[tiab]) AND (improvement*[tiab] OR achievement*[tiab] OR ability[tiab] OR gain*[tiab] OR success*[tiab] OR performance*[tiab])) OR (prevent*[tiab] AND (failure*[tiab] OR dropout*[tiab] OR gap[tiab] OR gaps[tiab])) OR (improve*[tiab] AND (grade*[tiab] OR score*[tiab]))) AND (trial[tiab] OR random*[tiab] OR ((control*[tiab] OR comparison*[tiab] OR intervention*[tiab]) AND (group*[tiab] OR school*[tiab] OR student*[tiab])) OR cohort*[tiab] OR longitudinal[tiab] OR prospective[tiab] OR retrospective[tiab]) AND publisher[sb]

**Web of Science Core Collection**

1,942 hits

**...MoreTOPIC:** (("academic*" or "educational*" or "remedial*" or "home*" or "out-of-school*" or "non-school*" or "family" or "parent*" or "caregiver*" or "liaison*") NEAR/2 ("program*" or "support*" or "intervention*" or "training*" or "game*" or "app" or "apps") or (("compensatory" or "supplementary" or "extra-curricular") NEAR/2 "education*") or "tutoring" or "tutors") *AND* **TOPIC:** ((("academic*" or "educational*" or "reading" or "math*" or "writing" or "student*") NEAR/2 ("improvement*" or "achievement*" or "ability" or "gain*" or "success*" or "performance*")) or ("prevent*" NEAR/2 ("failure*" or "dropout*" or "gap*")) or ("improve*" NEAR/5 ("grade*" or "score*"))) *AND* **TOPIC:** (("trial" or "random*" or (("control*" or "comparison*" or "intervention*") NEAR/5 ("group*" or "school*" or "student*")) or "cohort*" or "longitudinal" or "prospective" or "retrospective"))

**Timespan:** All years. **Indexes:** SCI-EXPANDED, SSCI, A&HCI, CPCI-S, CPCI-SSH, ESCI.

## Characteristics of included studies

#### Description of interventions

**Effective interventions**

*Parent mediated interventions.* Elleven interventions were delivered through parents or caregivers (Bronstein et al. 1998; Chassen 1978; Esters & Levant 1983a; Feldman et al. 1983; Flynn et al. 2012; Mehran & White 1988; Rasinski & Stevensen 2005; Reutzel et al. 2006; Shuck et al. 1983; Wise 1972). Eight of these interventions combined parent training with parent tutoring in reading and one in reading and math*.* The parent training typically included training in parental school involvement at home, such as use of positive reinforcement, training in homework support, and guidance in structure and routines (e.g. Teach Your Children Well program, Flynn et al. 2012). Parent training also included training in tutoring techniques such as direct instruction and paired reading. and other model-lead-test techniques (a tutor models a skill - the tutor and the tutee practices the skill together - the tutee practice skill alone and receive feedback and correction). One intervention, The Self-Esteem Method, did not directly target academic elements, rather the program taught parents specific techniques to improve family communication, enhance child self-esteem, define limits, foster identity development and educationally favorable values (Esters & Levant 1983b).

*Child tutoring interventions.* Nine interventions (Andersen et al. 1979; Blachman et al. 2004; Black & Somers 2009; Bridges 2011; Carbone 2009; Cloward 1967; Cole & Hilliard 2006; Harper 2012; Morris et al. 1991) included child tutoring elements in reading (*N*=6) or both reading and math (*N*=3). Eight interventions used one to one tutoring delivered by a paraprofessional (*N*=2), a teacher (*N*=2), adult volunteers (*N*=1), student volunteer (*N*=2) or a web-based computer program (*N*=1). One intervention (Black 2009) combined group based- and individual tutoring by teachers with web-based tutoring by computer, cooperative learning, partnered reading and board games. Five studies utilized direct instruction as tutoring technique, and two of these studies also utilized model-lead-test techniques. Tutoring in reading included paired reading, being read to, and playing reading games, as well as training in letters, phonics, phonemes, dictation, word decoding, recognition and categorization. Elements in math tutoring included practicing computation (addition, subtraction, multiplication, division, fractions, equations), measurement understanding, geometry, probability and playing math games.

*Combined interventions.* Six interventions combined parent training elements with child tutoring elements and was effective on reading (Trovato et al. 1980; Tolan et al. 1994; Tolan et al. 2009; Morrow & Young 1997; Weine et al. 1993) and math (Fantuzzo et al. 1995). Parent training in positive parental involvement (positive reinforcement, rewards, corrective feedback, positive communication about school, homework structure and routines) was combined with tutoring at school from peers (*N*=2) or paraprofessionals (*N*=1) or tutoring with parents at home (*N=3).*

*After school programs.* Three effective after school programs were identified. (Eash et al. 1981; Harpine & Reid 2009; Zosky & Crawford 2003). The Child parent Expansion program (Eash et al. 1981) was conducted at child-parent centers and was found effective in math skills. The program emphasized parental involvement in school and included a structured language skills program as well as nutrition and health care advice. A second after school tutoring program was effective in math, language arts and grade point average (Zosky & Crawford 2003). Tutoring elements was delivered by a social service employee and included supervising homework completion and reinforcing academic performance with incentives. Camp Sharigan (Harpine & Reid 2009), was a one week culturally sensitive group intervention based on the principles of efficacy retraining to teach reading skills. The program was run by trained volunteers and focused on word recognition, spelling, writing, storytelling and phonics training. The intervention had effect on reading skills despite its short duration.

*Other effective intervention.* Mental Contrasting with Implementation Intentions (Duckworth et al. 2013) taught children a metacognitive self-regulation strategy to apply to achieve self-defined academic goals. A trained interventionist worked with children in small groups to teach them to define a goal, imagining obstacles to achieving that goal, and create a plan to overcome the obstacles.

**Ineffective interventions**

Two studies (Munoz et al. 2008; Munoz et al. 2011) evaluated cohorts of children receiving various forms of after school tutoring in reading and math. Several different process elements were applied to conduct tutoring (in group, 1on1, at home, at after school program), but poorly described. Results showed positive trends but did not reach statistical significance. One study (Schinke et al. 2000) evaluated an educational enhancement program at a local boys and girls club. The program included creative writing, leisure reading, homework support, peer tutoring and board games, and had positive results on reading, math and grade point average post intervention and at follow up. Between-group differences, however, did not reach statistical significance. One ineffective intervention in an underpowered study (*N=*38; Powell-Smith et al. 2000) included five weeks of structured parent tutoring mainly in form of reading aloud and receiving correction and feedback from trained parents. One intervention (Ellis 1996) included children reading alone, reading together with parents, answering comprehension questions, and receiving praise and encouragement from parents. Many uncontrolled adaptations were made to the intervention. Children in the intervention group significantly improved on test of reading passages, but not on reading ability test, comprehension test, or graded word list. One intervention was an after-school program for boys focused on sports and homework support (Cid 2014), but no further description of the intervention was given.

#### Complete study characteristics tables

|  | Paraprofessional Reading Tutors: Assessment of the Edmark Reading Program and Flexible Teaching |
| --- | --- |
| **Authors** | Andersen, B. L., Licht B. G., Ullmann, R. K., Buck, S. T. & Redd, W. H. |
| **Year of publication** | 1979 |
| **Publication type** | Journal article |
| **Methods** | Study design: Randomized controlled trial  Duration pre-post: 9-11 weeks  Duration follow up: -  Intention-to-treat: unclear |
| **Participants** | Country: USA, Champaign-Urbana (Illinois).  Setting: Children met with tutors after school. Unclear if they met at school or other.  Type of risk: Indicated risk. Low-mid socioeconomic range.  N in intervention conditions: Unclear  N in control conditions: Unclear  Total N: 36 children  How many eligible/approached for participation: (% of eligible participated= reach*):  unclear  Mean age: 6.9 years old (range 6.3 – 8.1 years old)  Gender: 19 boys, 17 girls |
| **Interventions and comparisons** | Condition 1 (Edmark Reading program): After-school tutoring by undergraduate paraprofessionals for first-grade children with reading difficulties. Tutoring includes 150 word-recognition lessons, each of which introduces one new word. The tutor uses direct instruction (model-lead-test) to teach new words. Tutoring also includes matching pictures with phrases and reading stories using words taught.  Condition 2 (Flexible Reading): After-school tutoring by undergraduate paraprofessionals for first-grade children with reading difficulties. Pupils are taught the same 150 words as Group 1, but were allowed a complete flexibility in the manner of word presentation and review. Techniques used were flash cards, word wheels and word bingos.  Duration: 40 min, 3 times each week for 9 weeks  Comparison 1: Educational activities not directly related to reading  Duration: Unclear |
| **Outcomes** | Reading: Criterion-referenced test. Gates-MacGinitie and criterion-reference test |
| **Funding source** | Not stated |
| **Coding** | + effects on reading |
| **Notes** | Poor reporting on methods |

|  | Effects of intensive reading remediation for second and third graders and a 1-year follow up |
| --- | --- |
| **Authors** | Blachman, B. A., Fletcher, J. M., Clonan, S. M., Schatschneider, C., Francis, D. J., Shaywitz, B. A., & Shaywitz S. E. |
| **Year of publication** | 2004 |
| **Publication type** | Journal article |
| **Methods** | Study design:  Randomized controlled trial – assigned within schools, grade and gender to condition  Intention to treat:  No  Duration pre-post:  8 months  Duration follow up:  12 months after intervention end. |
| **Participants** | Country:  New York, USA  Setting:  Unclear  Identified risk:  Low scores on reading test (WRMT-R)  N in intervention conditions: 37  N in control conditions: 32  Total N: 69  How many eligible/approached for participation: (% of eligible participated= reach)  100. Reach= 69%  Mean age: second and third graders (USA)  Gender: 42 boys |
| **Interventions and comparisons** | Condition 1:  Intensive reading intervention; in 5 parts:  explicit and systematic instruction to help children develop an understanding of the phonologic and orthographic connections in words, phoneme analysis and blending particular syllable patterns, fluency building activity, oral reading practice, dictated words  Duration:  50 min one-to-one tutoring 5 days a week for 8 months. Average 105 hours of tutoring  Comparison 1:  Control group. Treatment as usual. If remedial reading help was provided some were offered that from their own school.  Duration:  - |
| **Outcomes** | Reading:  Woodcock Reading mastery test-Revised (WRMT-R)  Word identification subtest and Word Attack subtest (Form G)  Gray Orla reading test – third edirtion (GORT-3)  Word Reading (modified by Foorman &Schatschenider). Read 41 words graded in difficulty.  Word reading efficiency . read as quickly as possible a list of 104 words  Wide Range Achievement Test 3 (WRAT3). A spelling test  Blending Phonemes – Words. Listen to words segmented by phonemes and asked to blend the sounds together to make a word.  Segmenting Phonemes  Phoneme Elision  Noword repetition. Repeat 25 nonsense words  Rapid naming of letters  Spelling Dictation  Math:  Woodcock-Johnson Psycho-educational Battery-Revised (WJ-R) Test of Achievement, Calculation subtest, and Applied Problems subtest.  Other:  Fidelity |
| **Funding source** | Center Grant P50HD25802 awarded to Yale University School of Medicine by the National Institute of Child Health and Human Development. |
| **Coding** | + effects on reading |
| **Notes** | Low statistical power and many statistical tests. |

|  | The Evaluation of Enhanced Academic Instruction in After-School Programs. Final Report |
| --- | --- |
| **Authors** | **Black, A.R & Somers, M.A** |
| **Year of publication** | 2009 |
| **Publication type** | Evaluation report, not peer reviewed |
| **Methods** | Study design: Individually randomized controlled trial  Intention-to-treat: Yes  Duration pre-post: approx. 6-9 months |
| **Participants** | Country: US  Setting: After school at a primary school  Identified risk: students below local standard for grade level in reading or math, but not by more than two years from low-income families  N in intervention conditions:  Cohort 1. Math intervention: 634, reading intervention: 504  N from cohort 1 in cohort 2. Math intervention: 144, reading intervention: 98  N not from cohort 1 in cohort 2. Math intervention: 317, reading intervention: 245  N in control conditions:  Cohort 1: Math control: 510, reading control: 401  N from cohort 1 in cohort 2. Math control: 105, reading control: 74  N not from cohort 1 in cohort 2. Math control: 226, reading control: 200  Total N: Math: 1144 reading: 905  How many eligible/approached for participation: (% of eligible participated= reach): Unclear  Mean age: 8.6 (grade 2-5)  Gender: Math cohort 1: intervention; 47% male, control; 43% male. Math cohort 2: intervention; 42% male, control; 46% male. Reading: intervention; 53% male, Control; 47% male |
| **Interventions and comparisons** | Condition 1:  **Math intervention:** adaptation of *Mathletics*, an after-school program built around five math-themes; numbers and operations, measurement, geometry, algebra and functions, and data analysis and probability. Combination of individualized individual- and group instruction, computation and problem solving using a warm up exercise (directed instruction from teacher), instruction and practice material on paper. Also includes board games and progress monitoring.  **Reading intervention:**  *Adventure Island,* a directed instruction approach in groups using the following programs:  For 1. graders: *Aplhie’s Lagoon,* a phonics program designed to build phonemic awareness, letter-sound correspondence, world-level blending, and segmenting. Partnered and individual reading of progressively more complex stories with instruction from teacher.  For 2.graders and Above: Partnered reading and other cooperative learning techniques using fiction and nonfiction texts providing instruction in vocabulary, advanced phonics, fluency, comprehension strategies, and story elements.  Duration:  Math Cohort 1; 189 min per week, 75 hours total. Math cohort 2: 171 min per week, 72.5 hours total.  Reading cohort 1: 177 min per week, 76 hours total. Reading cohort 2: 175 min per week, 76 hours total.  Comparison math and reading:  Business as usual in regular after-school program: various tutoring, instruction and homework support  Duration:  Reading cohorts: 5 hours on average in a year. Math cohort 1; 11 hours on average, math cohort 2; 16 hours on average |
| **Outcomes** | Reading:  Dynamic Indicators of Basic Early Literacy Skills (DIBELS)  Math:  Stanford Achievement Test Series, 10th ed. (SAT 10).  Other:  Teacher rating of academic behavior, fidelity, dose and other implementation measurements |
| **Funding source** | National Center for Education Evaluation and Regional Assistance at the U.S. Department of Education’s Institute of Education Sciences (IES), |
| **Coding** | + significant effects on math  ÷ not significant effects on reading (However, positive results) |
| **Notes** | Study sites were particularly well-functioning sites, which might limit generalizability. |

|  | The Correlation Between After School Tutoring and Reading Scores of At-Risk third Graders |
| --- | --- |
| **Authors** | **Bridges, C** |
| **Year of publication** | 2011 |
| **Publication type** | Doctoral dissertation |
| **Methods** | Study design: quasi experimental, nonequivalent control  Intention to treat: not relevant  Duration pre-post: approx. 6-9 months |
| **Participants** | Country: US  Setting: After school at a primary school  Identified risk: Low performers on academic assessments  N in intervention conditions: at risk: 66, not at-risk: 80  N in control conditions: 86  Total N: 232  How many eligible/approached for participation (% of eligible participated= reach): Unclear  Mean age: third graders, not specified  Gender: Unclear |
| **Interventions and comparisons** | Condition 1:  After school-tutoring from teachers, used a variety of teaching material in groups of six students grouped together for different reasons (behavioral problems mentioned as example). No further specification  Duration: a school year, no further specification  Comparison 1:  No tutoring, no further specification  Duration: a school year, no further specification |
| **Outcomes** | Reading:  Texas Assessment of Knowledge and Skill (TAKS). Measures skills in reading, writing, math, science and social studies at various grade levels (TEA, 2009).  Math:  TAKS  Other:  TAKS |
| **Funding source** | Not specified |
| **Coding** | + effects on reading |
| **Notes** | Limited information about sample and conditions |

|  | Preventing Middle School Adjustment Problems for Children from Lower-Income Families: A program for Aware Parenting |
| --- | --- |
| **Authors** | **Bronstein, P., Duncan, P., Clauson, J., Abrams, G.L., Yannett, N., Ginsberg, G., & Milne, M.** |
| **Year of publication** | 1998 |
| **Publication type** | Journal article, Journal of Applied Developmental Psychology |
| **Methods** | Study design: Individually randomized controlled trial. Families were stratified based on high- or low academic performance in their children and randomized to treatment or control  Intention to treat: No  Duration pre-post: 4-5 months  Duration follow up: one year after intervention |
| **Participants** | Country: US  Setting: Not specified  Identified risk: Low performers on academic assessments  Indicated risk: Low income families with children transitioning to middle school  N in intervention conditions: 10  N in control conditions: 13  Total N: 23  How many eligible/approached for participation (% of eligible participated= reach): 35 families invited “almost all” from a subsample of a larger sample of low income families. Reach in subsample=48%  Mean age: 9-12, with mean of 10.7 years  Gender: Majority of girls, not further specified |
| **Interventions and comparisons** | Condition 1:  Parent training/support groups based on the Aware Parenting Model, focusing on parental support, guidance and attentiveness.  Duration: Two-hour weekly session for 11 weeks.  Condition 2: Control condition, not further specified  Duration: unclear |
| **Outcomes** | Grade point average  Teacher rating of behavioral and psychological problems. |
| **Funding source** | National Institute of Health, grant number R01 MH40741 |
| **Coding** | + effects on GPA |
| **Notes** | Limited academic outcomes |

|  | The Effects of an After-School Tutoring Program on the Pennsylvania System of School Assessment |
| --- | --- |
| **Authors** | **Carbone, P.M** |
| **Year of publication** | 2009 |
| **Publication type** | Doctoral dissertation |
| **Methods** | Study design: Quasi experimental (Ex-post facto design).  Intention to treat: N/A  Duration pre-post: 7-8 months |
| **Participants** | Country: USA  Setting: Public middle school after school hours  Identified risk: Student were eligible if they scored basic on PSSA  N in intervention conditions: Math tutoring: 70, reading tutoring: 82  N in control conditions: Math control: 71, reading control: 93  Total N: 316  How many eligible/approached for participation (% of eligible participated= reach): 316. Reach=100%  Mean age: not specified. Fifth, sixth, seventh, and eight graders  Gender: Unclear |
| **Interventions and comparisons** | After school tutoring in reading: Students working independently on their weakest reading skill using “Reading Labs” and “Specific Skill Series Labs” material, or, “Skills Tutor”, a web-based tutor prescribing instruction and tasks based on needs with monitoring and feedback.  Duration: one hour, twice a week for 66 months  After school tutoring in math:  “America’s Choice Mathematical Navigator”, a twelve-module math program with instruction and computation in basic math concepts based on needs, or, “Skills Tutor”, a web-based tutor prescribing instruction and tasks based on needs with monitoring and feedback.  Duration: one hour, twice a week for 7 months  Control condition: Student who were not interested or had no response to request to participate in the intervention. |
| **Outcomes** | Reading and math:   - Pennsylvania System of School Assessment. Combinations of multiple-choice and open-ended questions (reading comprehension, problem solving and computation)   4sight benchmark assessment. One-hour benchmark test given to gauge student growth |
| **Funding source** | Not reported |
| **Coding** | + significant positive effects of the math intervention  ÷ not significant effects of the reading intervention |
| **Notes** | Limited reporting. No demographics on subjects, control group comprised of students who declined participation, and no analyses of heterogeneity. Unclear reporting of tutoring. |

|  | Giving a second chance: an after-school programme in a shanty town interacted with parent type: lessons from a randomized trial |
| --- | --- |
| **Authors** | **Cid, A** |
| **Year of publication** | 2014 |
| **Publication type** | Journal article |
| **Methods** | **Study design:** Randomized controlled trial  Duration pre-post: 9 months  Duration follow up: -  Intention-to-treat*: yes |
| **Participants** | **Country:** Uruguay  Setting: Los Pinos education center, after school program for boys  **Type of risk:** Indicated risk. Living low ses neighborhood  N in intervention conditions: 28  N in control conditions: 26  **Total N: 54 (**8 attrition)  How many eligible/approached for participation: (% of eligible participated= reach): unclear  **Mean age:** intervention group: 6.32, control group: 6.48  Gender: all boys |
| **Interventions and comparisons** | Condition 1): After-school program for boys (Los Pinos) were children practice sports and receive help with their homework after school hours. No further specification  Duration: unclear  Comparison 1: unclear  Duration: Unclear |
| **Outcomes** | Teacher set school reports of academic performance and behavior (scale of 1 to 10) |
| **Funding source** | Not stated |
| **Coding** | Effects on academic performance (gpa) when controlled for parental involvement. Ineffective with low parental involvement |
| **Notes** | Poor measure of academic performance, after school academic activities not described. No practice elements to code |

|  | The Effects of Parent Training in Behavior Modification and Transactional analysis on the Reading Achievement, School attitudes, and Self-Concept of Remedial Readers in a Remedial Reading Program |
| --- | --- |
| **Authors** | **Chassen, L** |
| **Year of publication** | 1978 |
| **Publication type** | Dissertation |
| **Methods** | Study design: Individually randomized controlled trial  Duration pre-post: Ten weeks  Intention-to treat: unclear  Duration follow up: - |
| **Participants** | Country: USA, Colorado  Setting: Interventions delivered in the home  Type of risk: Identified risk. Two years behind in reading comprehension  N in intervention conditions: Group 1 = 20, Group 2 = 20  N in control conditions: 20  Total N: 60  How many eligible/approached for participation (% of eligible participated= reach): unclear  Mean age: Unclear (seventh – eighth graders)  Gender: Unclear |
| **Interventions and comparisons** | Condition 1: Parents established a 1 hour reading and study period 3 days a week based in the principles and techniques of behavior modification.  Condition 2: Parents established a 1 hour reading and study period 3 days a week based in the principles and techniques of Transactional Analysis.  Duration: 1 hour reading and study period on the three days their child did not attend The Learning House. March 1977 – June 1977.  Comparison 1: No parent training, but children received remedial reading instruction.  Duration: March 1977 – June 1977 |
| **Outcomes** | Reading: Metropolitan Achievement Test, Advanced Level Form F  Other: Self-concept, school attitudes |
| **Funding source** | Not stated |
| **Coding** | +reading |
| **Notes** | Poor reporting |

|  | Studies in tutoring (report with no title name) |
| --- | --- |
| **Authors** | **Cloward, R. D.** |
| **Year of publication** | 1966 |
| **Publication type** | Unpublished report |
| **Methods** | Study design: Individually randomized controlled trial  Duration pre-post: 5 months  Duration follow up: -  Intention-to-treat: no |
| **Participants** | Country: USA, New York  Setting: Intervention is delivered after school in tutorial centers in neighborhood elementary schools  Type of risk: Identified risk. Low-achieving pupils reading below grade level  N in intervention conditions: 356 (attrition: 54)  N in control conditions: 157 (attrition: 28)  Total N: 513  How many eligible/approached for participation (% of eligible participated= reach): unclear  Mean age: 9 years (54% in experimental and 58% of control are fourth graders)  Gender: 47% girls, 53% boys in experimental group. 50% girls and 50% boys in control group |
| **Interventions and comparisons** | Condition 1: High school pupils helping fourth- and fifth graders with their homework after school 1-2 hours a week. The main objective is to improve their pupils’ language skills.  Duration: 1-2 hours a week for 5 months  Comparison 1: No tutoring  Duration: Unclear |
| **Outcomes** | Reading: New York Tests of Growth in Reading  Other: grades, academic behavior, and attitudes and aspirations |
| **Funding source** | Unclear |
| **Coding** | +reading |
| **Notes** | High attrition |

|  | The effects of web-based reading curriculum on children’s reading performance and motivation |
| --- | --- |
| **Authors** | **Cole, J.M & Hilliard, V.R** |
| **Year of publication** | 2006 |
| **Publication type** | Journal article |
| **Methods** | Study design: Randomized controlled trial  Duration pre-post: eight weeks  Intention-to treat: no  Duration follow up: - |
| **Participants** | Country: USA  Setting: Public elementary school in inner city California  Type of risk: Identified risk. Two or more grade levels behind average in reading  N in intervention conditions: 18  N in control conditions: 18  Total N: 36 44 at pre-test, 12 attrition  How many eligible/approached for participation (% of eligible participated= reach): 62 eligible approached. Reach= 58%  Mean age: n.a. range from 8 years and 6 months to 10 years and 4 months  Gender: g=19, b=17. |
| **Interventions and comparisons** | Condition 1: Web-based reading instruction (reading upgrade). An active learning environment aimed to be student-centered, interactive, exploratory, contextualized, intentional, reflective and collaborative. Includes training in phonics, phonemic awareness, common sight words, word decoding, multi syllable words, reading comprehension, and reading practice  Duration: 90 minutes two times a week for eight weeks  Comparison 1: Traditional school practice using direct reading instruction  Duration: 90 minutes two times a week for eight weeks |
| **Outcomes** | Reading: Developmental reading assessment (Beaver, 1998), Wide Range Achievement Test 3 (Wilkinson, 1993)  Other: Motivation measures |
| **Funding source** | National Science Foundation and Ronald E. McNaire Scholars Program |
| **Coding** | +reading |
| **Notes** |  |

|  | From fantasy to action: Mental contrasting with implementation intentions (MCII) improves academic performance in children |
| --- | --- |
| **Authors** | **Duckworth, A. L., Kirby T., Gollwitzer A., & Oettingen G** |
| **Year of publication** | 2013 |
| **Publication type** | Journal article |
| **Methods** | Study design: randomized controlled longitudinal study  Intention to treat: No  Duration pre-post: 1212 weeks  Duration follow up: 8 weeks |
| **Participants** | Country: USA  Setting: At an elementary school  Indicated risk:  Low income/socioeconomically disadvantaged children, indicated for 85% of the sample by eligibility for free/reduced prince lunch.  N in intervention conditions: unclear  N in control conditions: unclear  Total N: 77  How many eligible/approached for participation (% of eligible participated= reach): reach=94%  Mean age: fifth grade, 11 years, not further specified  Gender: unclear |
| **Interventions and comparisons** | Condition 1:  Learn a metacognitive self-regulation strategy: *Mental Contrasting with Implementation Intentions (MCII)*, strategy for imagining a goal with its obstacles and having a plan of how to overcome the obstacles. Asked to apply it to various self-defined academic wishes.  Duration: three one-hour sessions.  Comparison 1: Positive Thinking Control exercise: indulging in positive thoughts on future academic achievement, without instructions to make plans to overcome obstacles.  Duration: three one-hour sessions. |
| **Outcomes** | GPA: Grade point average change from first to third quarter for math, writing, social studies and reading grades  Other:  Attendance  Conduct  Classroom behavior rated by teacher |
| **Funding source** | Unclear |
| **Coding** | + Grade point average (GPA) |
| **Notes** | Limited reporting |

|  | Evaluation of Multiple Outcomes in the Child Parent Center Compensatory Education Program: Year 2. |
| --- | --- |
| **Authors** | **Eash, M. J.** |
| **Year of publication** | 1981 |
| **Publication type** | Conference Journal article |
| **Methods** | Study design: Quasi-experimental, nonequivalent control group design  Duration pre-post: 1, 2 and 3 years after intervention started  Duration follow up: 3 years  Intention-to-treat: n.a. |
| **Participants** | Country: USA, Midwest  Setting: Intervention delivered in child parent centers  I: Children from low-income families with academic achievement below national norms.  N in intervention conditions (5 year olds): 227  N in control conditions (5 year olds): 304  Total N: 531  N in intervention conditions (8 year olds): 54  N in control conditions (8 year olds): 425  Total N (8 year olds): 479  How many eligible/approached for participation (% of eligible participated= reach): Unclear  Mean age: 5 year olds and 8 year olds, not further specified  Gender: Unclear |
| **Interventions and comparisons** | Condition 1: Child Parent center program and Child Parent Expansion Program both with the same content. Both programs emphasize direct parental involvement in the schools and a structured language/basic skills program.  Duration: Two days (four half-days) a month for three years  Comparison 1: Children living in the same sort of disadvantaged community, regular classroom activity.  Duration: Unclear |
| **Outcomes** | Reading: Vocabulary, Reading Comprehension, Spelling, Word Analysis  Math: Math Concepts and Math Problems  Measured with Iowa Test of Basic Skills (ITBS)  Parental engagement:  Modified version of Dolan’s home support interview (1978) |
| **Funding source** | Unclear |
| **Coding** | + significant effects on math achievement  ÷ not significant effects on reading achievement (but positive results) |
| **Notes** | Limited reporting |

|  | Parent-Child Reading Programs: Involving Parents in the Reading Intervention Process |
| --- | --- |
| **Authors** | **Ellis, M.G** |
| **Year of publication** | 1996 |
| **Publication type** | Dissertation |
| **Methods** | Study design: Randomized controlled trial  Intention to treat: no  Duration pre-post:  12 weeks.  Duration follow up: - |
| **Participants** | Country: USA  Setting: Weekly parent training at school for 12 weeks, parent tutoring at home between sessions  Identified risk: Children identified as at least six months below grade level in reading  N in intervention conditions: 20  N in control conditions: 38  Total N: 82, 24 attrition  How many eligible/approached for participation (% of eligible participated= reach): approx. 250, 82 consented (reach=32.8%)  Mean age: 8.03 (range 7-9)  Gender: 50% female |
| **Interventions and comparisons** | Condition 1: Parent training based around four core components: relaxed reading, paired reading, comprehension questions and praise/encouragement. Uncontrolled adaptations to intervention. Dose not reported, so the adaptations will not be coded. |
| **Outcomes** | Numbers of errors on graded oral reading lists, number of errors on graded oral reading passages, number of errors on graded oral comprehension questions, self concept of reading ability |
| **Funding source** | Not mentioned |
| **Coding** | + effects on numbers of graded passages  ÷ no effects on three of four reading measures |
| **Notes** | Positive results, but low powered |

|  | The effect of two parent counselling programs on rural low-achieving children |
| --- | --- |
| **Authors** | **Esters, P., & Levant, R. F.** |
| **Year of publication** | 1983 |
| **Publication type** | Journal article |
| **Methods** | Study design: Individually randomized controlled trial  Intention to treat: no  Duration pre-post:  10 weeks.  Duration follow up:  12 weeks |
| **Participants** | Country: USA  Setting: Unclear  Identified risk: low-achieving students based on California Achievement test and GPA of 75 or below.  Indicated risk: Low SES  N in intervention conditions: 11 in condition 1, 11 in condition 2  N in control conditions: 11  Total N: 33  How many eligible/approached for participation (% of eligible participated= reach): Unclear  Mean age: Third and fourth grade, younger than 11 years and 4 months. Not further specified  Gender: 24 boys and 9 girls |
| **Interventions and comparisons** | Condition 1: Systematic Training of Effective Parenting (STEP), an Adlerian program  Duration: 10 weeks. 1 and ½ -hour sessions held weekly.  Comparison 1: Self-esteem (SEM), and eclectic program  Duration: 10 weeks. 1 and ½ -hour sessions held weekly.  Comparison 2: wait-list control group |
| **Outcomes** | GPA: Grade point average for language arts, arithmetic’s, social studies and science.  Other:  Self-esteem assessed by  1) the Piers-Harris Children’s Self-Concept Scale (PHS), a self-report and  2) the Coopersmith Behavior Rating Form for Teachers (CBRF), a teacher report. |
| **Funding source** | Not mentioned |
| **Coding** | + effects on GPA and language arts |
| **Notes** |  |

|  | Effects of Parent Involvement in Isolation or in Combination With Peer Tutoring on Student Self-Concept and Mathematics Achievement |
| --- | --- |
| **Authors** | **Fantuzzo, J.W., Davis, G.Y., & Ginsberg, M.D** |
| **Year of publication** | 1995 |
| **Publication type** | Journal article |
| **Methods** | Study design: RCT with individual randomization  Intention to treat: no  Duration pre-post: 10 weeks  Duration follow up: - |
| **Participants** | Country: USA  Setting: Regular classrooms, during and after school hours, in a public school in a large urban city,  Identified risk: identified by teachers as evidencing low-performance in basic mathematics  N in intervention conditions: 24 in condition 1, 23 in condition 2  N in control conditions: 25  Total N: 72 (attrition=6)  How many eligible/approached for participation (% of eligible participated= reach): Unclear  Mean age: 10 years and seven months  Gender: 51% female |
| **Interventions and comparisons** | 1. Parent involvement (PI): same as practice control condition + PI training and facilitation by meetings and telephone contact. Core components were regular positive home-school communication and parent-student celebration of student efforts and accomplishments.  Duration: 90 minutes a week, for 10 weeks  2. PI + reciprocal peer tutoring: PI as above + regular classroom procedure carried out in dyads where each dyad solved problems together and set their own goals and rewards.  Duration: 90 minutes a week, for 10 weeks  3.Practice control condition: regular classroom procedure (5 min multiplication table drill, needs based computational problems using flash cards and problem sheets)  Duration: 90 minutes a week, for 10 weeks |
| **Outcomes** | Math:   - Curriculum-based computation test. 48 computational problems taken - Standford Diagnostic Mathematics test, 3^rd^ ed. (Beatty et al., 1986). Measures basic mathematic concepts and computations.   Other:  Self-Perception Profile for Children (Harter, 1985). Measures selv-percieved scholastic competence, social acceptance, behavioral conduct, athletic competence, physical appearance, and global self-worth. |
| **Funding source** | In part by grants from the Drug-Free Schools and Communities Emergency Grant Program of the Commonwealth of Pennsylvania and the University of Pennsylvania Research Foundation |
| **Coding** | + Effects on math abilities |
| **Notes** |  |

|  | Parent and paraprofessional tutoring of academically-delayed and learning disabled children: study #1 |
| --- | --- |
| **Authors** | **Feldman, MA., Bownman, T.G., & Feyen, L.** |
| **Year of publication** | 1983 |
| **Publication type** | Journal article |
| **Methods** | Study design: non-randomized study with matched controls  Intention to treat: unclear  Duration pre-post: 8.4 months, SD 4.03 months |
| **Participants** | Country: Canada  Setting: home during summer  Identified risk:  Indicated risk: Parent/teacher/student felt that the student could benefit from the program. Selected from suspected learning disabled children sees at a neuro-psychology clinic.  N in intervention conditions: 4  N in control conditions: matched controls: 4, “random” controls: 8  Total N: 16  How many eligible/approached for participation (% of eligible participated= reach): Unclear  Mean age: Parent tutoring group:12.2. Matched control group: 12.3. “Random” control group: 8.8  Gender: “Random control group: seven males, one female”. Other groups not reported |
| **Interventions and comparisons** | Condition 1, Home tutoring from parents:  Trained parents provided individualized tutoring with emphasis on positive reinforcement. Practice elements were oral reading and reading comprehension-tasks, frequent use of praise, reward system, and corrective feedback.  Duration: three to five session per week over the summer  Comparison 1: control condition not specified. |
| **Outcomes** | Reading:  Wide Range Achievement Test (Jastak & Jastak, 1964). |
| **Funding source** | Not reported |
| **Coding** | + Effects on reading achievement |
| **Notes** | Poor reporting |

|  | Effects of individual direct-instruction tutoring on foster children’s academic skills: A randomized trial |
| --- | --- |
| **Authors** | **Flynn, R. J., Marquis, R. A., Paquet, M. P., Peeke, L. M. & Aubry, T. D.** |
| **Year of publication** | 2012 |
| **Publication type** | Journal article |
| **Methods** | Study design: Individually randomized controlled trial  Duration pre-post: approx. 28 weeks  Duration follow up: -  Intention-to-treat: no |
| **Participants** | Country: Canada, Ontario  Setting: Intervention delivered in the foster home  Type of risk: Indicated risk. Foster children living in foster families  N in intervention conditions: 42 (attrition=12)  N in control conditions: 35 (attrition=1)  Total N: 77 (attrition=13)  How many eligible/approached for participation (% of eligible participated= reach): Unclear  Mean age: 10.7 (SD = 1.6)  Gender: Experimental = 50% boys, 50% girls  Control = 42.9% boys, 57.1% girls |
| **Interventions and comparisons** | Condition 1: Foster parents trained in the *Teach Your Children Well* program *(*direct instruction and behavior management), tutoring children in reading and math  Duration: Approx. 3 hours a week for 28 weeks  Comparison 1: Wait list business as usual  Duration: Approx. 28 weeks |
| **Outcomes** | Reading: Sentence comprehension, Word reading  Math: Math computation  All measured with WRAT4 (Wide Range Achievement Test – Fourth edition) |
| **Funding source** | Unclear |
| **Coding** | + effects on math  + effects on reading |
| **Notes** |  |

|  | Full title  Preliminary effects of a group-based tutoring program for children in long-term foster care |
| --- | --- |
| **Authors** | **Harper J., & Schmidt F.** |
| **Year of publication** | 2012 |
| **Publication type** | Journal article +manual |
| **Methods** | Study design: Indvidually randomized controlled trial.  Intention to treat: no  Duration pre-post: 25 weeks  Duration follow up: 12 weeks, reported in Harper et al., 2016. |
| **Participants** | Country: Canada  Setting: Long term foster care  Identified risk: behind in academic achievement but not lower than 70 IQ.  Indicated risk: Long-term foster care  N in intervention conditions: 33 (3 children withdrew from the study before post-testing)  N in control conditions: 35  Total N: 68 (attrition=3)  How many eligible/approached for participation (% of eligible participated= reach): unclear  Mean age: 10,1 years. 2^nd^ -8^th^ grade  Gender: 58% males in the intervention group, 57% males in the control group |
| **Interventions and comparisons** | Condition 1: Small groups-based tutoring using the *Teach Your Children Well* curriculum, using direct instruction and behavior management  Duration: 2 hours a week for 25 weeks.  Comparison 1: wait-list business as usual  Duration: 25 weeks |
| **Outcomes** | Reading: Wide Range Achievement Test Fourth Edition (WRAT-4)  Outcome measure not validated for Aboriginals, which were 80% of the sample.  Math: Wide Range Achievement Test Fourth Edition (WRAT-4)  Other: Other academic abilities, grades in single subjects |
| **Funding source** | The Ministry of Children and youth Services, Ministry of Education, and the Ministry of Training, Colleges and Universities |
| **Coding** | + Significant effects on reading  ÷ not significant effects on math (although Hedges G of .26), will code minus for math |
| **Notes** |  |

|  | Enhancing Academic Achievement in a Hispanic Immigrant Community: The Role of Reading in Academic Failure and Mental Health |
| --- | --- |
| **Authors** | **Harpine, E. C. & Reid, T.** |
| **Year of publication** | 2009 |
| **Publication type** | Journal article |
| **Methods** | Study design: Individually randomized controlled trial  Duration pre-post: 1 week  Duration follow up: 1 year  Intention-to-treat: unclear |
| **Participants** | Country: USA, Texas  Setting: Intervention delivered in an after-school center  Type of risk: Indicated risk based on Mexican descent and having English as second language (highest drop-out rate in the study state).  N in intervention conditions: 29  N in control conditions: 25  Total N: 54  How many eligible/approached for participation (% of eligible participated= reach): unclear  Mean age: Unclear (1 – 3 graders)  Gender: 32 boys, 22 girls |
| **Interventions and comparisons** | Condition 1: short-term group centered intervention, *Camp Sharigan*, designed to reduce academic failure and dropout among Hispanic youth in an inner-city neighborhood. Camp Sharigan focus on bolstering self-efficacy to restore children’s self-determination to learn. The program focuses on word recognition skills, and includes spelling, reading, writing and phonics training.  Duration: 10 hours over 1 week (2 hours a day for 5 days).  Comparison 1: Participated in the prevention interventions provided by the after-school program, which offered classroom-style education activities and one-on-one tutoring throughout the week.  Duration: 10 hours over 1 week (2 hours a day for 5 days). |
| **Outcomes** | Reading: Spelling, reading and improvement in sight words were measured with Howard Street assessment tests from The Howard Street Tutoring Manual |
| **Funding source** | Unclear |
| **Coding** | + effects on reading |
| **Notes** |  |

|  | Parent Tutoring as a Supplement to Compensatory Education for First-Grade Children |
| --- | --- |
| **Authors** | **Mehran, M. & White, K. R.** |
| **Year of publication** | 1988 |
| **Publication type** | Journal article |
| **Methods** | Study design: Individually randomized controlled trial  Duration pre-post: 6 months  Duration follow up: Unclear, end of first grade year approx. 3-4 months after posttest  Intention-to-treat: no |
| **Participants** | Country: USA  Setting: Intervention delivered in the home  Type of risk: Identified risk. Below average scores on reading ability  N in intervention conditions: 38  N in control conditions: 38  Total N: 76  How many eligible/approached for participation (% of eligible participated= reach): 80. Reach=95%  Mean age: Unclear (first graders)  Gender: Unclear |
| **Interventions and comparisons** | Condition 1: Structured parent tutoring reading program. The parents were instructed to use general tutoring techniques in teaching their children sounds and letters, basic sight words, blending sounds and decoding words.  Duration: 3 times a week forfor approx. 6 months  Comparison 1: BusinessBusiness as usual  Duration: approx.approx. 9 months |
| **Outcomes** | Reading: Woodstock Johnson Psycho Educational Battery (WJPEB), Comprehensive Test of Basic Skills (CTBS) and Harrison Criterion Referenced Test (HCRT) |
| **Funding source** | Not stated |
| **Coding** | + effects on reading |
| **Notes** |  |

|  | Helping Low Readers in Grades 2 and 3: An After-School Volunteer Tutoring Program |
| --- | --- |
| **Authors** | **Morris, D., Shaw, B., & Perney, J** |
| **Year of publication** | 1991 |
| **Publication type** | Journal article |
| **Methods** | Study design: Randomized controlled trial  Duration pre-post: 8 months  Duration follow up: -  Intention-to-treat: no |
| **Participants** | Country: USA  Setting: After school building close to school  Identified risk: Children identified with lowest reading scores in a group of slow readers  N in intervention conditions: 17  N in control conditions: 17  Total N: 34  How many eligible/approached for participation (% of eligible participated= reach): 50 (68& reach)  Mean age: second and third graders  Gender: Unclear |
| **Interventions and comparisons** | Condition 1: Howard Street Tutoring Program. After school tutoring from trained volunteers in reading and writing. Includes individualized contextual reading (echo reading/dictation, paired reading, reading aloud), word studying (categorization, games), writing with correction and feedback, and being read to  Duration: two hours a week for 8 months  Comparison 1: No tutoring |
| **Outcomes** | Reading:  *Word recognition*: Word lists derived from Basic Reading Vocabularies (Harris & jacbos, 1982) and a unspecified school basal reader  *Spelling:* dictation from the Qualitative Inventory of Word Knowledge (Schlagal, 1989)  *Basal passage reading*: Accuracy in reading aloud passages from the school basal reader (Ginn, 1983) with a self made scoring system. |
| **Funding source** | Unclear |
| **Coding** | + effects on reading |
| **Notes** |  |

|  | Supplemental Educational Services as a Consequence of the NCLB Legislation: Evaluating its Impact on Student Achievement in a Large Urban District |  |
| --- | --- | --- |
| **Authors** | **Munoz, M.A., Potter, P.P., & Ross, S.M** |  |
| **Year of publication** | 2008 |  |
| **Publication type** | Journal article |  |
| **Methods** | Study design: Quasi-experimental pre-post study with matched controls  Duration pre-post: 12 months  Duration follow up:  Intention-to-treat: n.a |  |
| **Participants** | Country: USA  Setting: Varied; at home, at school after hours, at after school program  Identified risk: Students eligible for supplemental educational service due to identified academic underachievement  N in intervention conditions: reading cohort: 677, math cohort: 333  N in control conditions: reading cohort: 408, math cohort: 583  Total N: Reading cohort: 1085, math cohort: 916  How many eligible/approached for participation (% of eligible participated= reach): n.a  Mean age: unclear, fourth-, fifth-, seventh-, eighth- and eleventh graders  Gender: reading cohort: 45.49% female, math cohort: 49.91% female |  |
| **Interventions and comparisons** | Intervention condition: Supplemental Educational Services (SES): Various forms of after school tutoring in math and reading. Several tutoring methods were used with different tutoring techniques (some in group, some one on one, some at home, some at afterschool).  Control condition: No SES |  |
| **Outcomes** | Kentucky Core Content Test (KTTC) in reading and mathematics. |  |
| **Funding source** | Unclear |  |
| **Coding** | ÷ reading and math |  |
| **Notes** |  |  |

|  | No Child Left Behind and Tutoring in Reading and Mathematics: Impact of Supplemental Educational Services on Large Scale Assessment |  |
| --- | --- | --- |
| **Authors** | **Munoz, M.A., Chang, F., & Ross, S.M** |  |
| **Year of publication** | 2011 |  |
| **Publication type** | Journal article |  |
| **Methods** | Study design: Quasi-experimental pre-post study with matched controls  Duration pre-post: 12 months  Duration follow up:  Intention-to-treat: n.a |  |
| **Participants** | Country: USA  Setting: Varied; at home, at school after hours, at after school program  Identified risk: Students eligible for supplemental educational service due to identified academic underachievement  N in intervention conditions: reading cohort: 393, math cohort:362  N in control conditions: reading and math cohort: 294  Total N: 862  How many eligible/approached for participation (% of eligible participated= reach): n.a  Mean age: unclear, fourth- to eighth graders  Gender: intervention cohort: 49.7% female, control cohort: 44.4% female |  |
| **Interventions and comparisons** | Intervention condition: Supplemental Educational Services (SES): Various forms of after school tutoring in math and reading. Several tutoring methods were used with different tutoring techniques (some in group, some one on one, some at home, some at afterschool).  Control condition: No SES |  |
| **Outcomes** | Kentucky Core Content Test (KTTC) in reading and mathematics. |  |
| **Funding source** | Unclear |  |
| **Coding** | ÷ reading and math |  |
| **Notes** |  |  |

|  | A Family Literacy Program Connecting School and Home: Effects on Attitude, Motivation, and Literacy Achievement |
| --- | --- |
| **Authors** | **Morrow, L. M. & Young J.** |
| **Year of publication** | 1997 |
| **Publication type** | Journal article |
| **Methods** | Study design: Cluster (classrooms) randomized controlled trial  Duration pre-post: Unclear, most likely around 1 year  Duration follow up: -  Intention-to-treat: no attrition or missing |
| **Participants** | Country: USA  Setting: Intervention delivered in the home and at school  Indicated risk: Children of minority backgrounds  N in intervention conditions: 28  N in control conditions: 28  Total N: 56  How many eligible/approached for participation (% of eligible participated= reach): unclear, most likely 100% reach  Mean age: 19 first graders, 17 second graders and 18 third graders  Gender: Unclear |
| **Interventions and comparisons** | Condition 1: Training parents in parental involvement in school at home, and involvingi parents in developmentally appropriate and culturally sensitive literacy activities with their children at home, in addition to school-based program to promote interest in reading.  Duration: 11 year, not further specified  Comparison 1: Only school-based program to promote interest in reading  Duration: 1 year, not further specified |
| **Outcomes** | Reading: Growth in achievement: A story retelling and a story rewriting test, A probed comprehension test, The California Test of Basic Skills,  Teacher ratings of children, Interviews with children to determine increased reading at home and with adults |
| **Funding source** | Unclear |
| **Coding** | + effects on reading |
| **Notes** |  |

|  | Parent Tutoring in Reading Using Literature and Curriculum Materials: Impact on Student Reading Achievement. |
| --- | --- |
| **Authors** | **Powell-Smith, K.A., Shinn, M.R., Stoner, G., & Good.R.H** |
| **Year of publication** | 2000 |
| **Publication type** | Journal article |
| **Methods** | Study design: Randomized controlled trial  Duration pre-post: 12 months  Duration follow up:  Intention-to-treat: no |
| **Participants** | Country: USA  Setting: Varied; at home, at school after hours, at after school program  Identified risk: Identified as low readers by their teachers  N in intervention conditions: condition 1: 13, condition 2: 13  N in control conditions: 12  Total N: 38, attrition 2.  How many eligible/approached for participation (% of eligible participated= reach): Unclear, however, 38 out of 40 parents who indicated interest in the project consented  Mean age: 7 years, 11 months (range: 7years,5months - 9years, 2months.)  Gender: 39% female |
| **Interventions and comparisons** | Intervention condition 1: Literature based home tutoring; Five week structured parent tutoring with individualized reading material chosen by children and parents. Main component was children reading 8-12minutes aloud with parents trained in correction and feedback, four times a week.  Intervention condition 2: curriculum based home tutoring: Five week structured parent tutoring with curriculum based reading material. Main component was children reading 8-12minutes aloud with parents trained in correction and feedback, four times a week.  Control condition: No home tutoring |
| **Outcomes** | Curriculum based measures of basal reading passages, Test of reading fluency using reading passages |
| **Funding source** | U.S. Department of Education, American Psychological Association Dissertation Research Award, a National Association of School Psychologists Student Research Grant, and a University of Oregon Graduate Student Research Award |
| **Coding** | ÷ reading |
| **Notes** |  |

|  | The Effects of Fast Start Reading: A Fluency Based Home Involvement Reading Program, On the Reading Achievement of Beginning Readers |
| --- | --- |
| **Authors** | **Rasinski, T. & Stevenson, B.** |
| **Year of publication** | 2005 |
| **Publication type** | Journal article |
| **Methods** | Study design: Individually randomized controlled trial  Duration pre-post: 11 weeks  Duration follow up: -  Intention-to-treat: no attrition or missing |
| **Participants** | Country: USA, Ohio  Setting: Intervention delivered in the home  Identified risk:: Low reading skills  N in intervention conditions: 15  N in control conditions: 15  Total N: 30  How many eligible/approached for participation): unclear  Mean age: 6  Gender: 16 girls, 14 boys |
| **Interventions and comparisons** | Condition 1: Fast Start parent-tutoring program. Incorporates elements of word decoding and especially reading fluency.  Duration: 10-15 min pr day for 11 weeks  Comparison 1: Regular parent involvement, without training.  Duration: Unclear |
| **Outcomes** | Reading: Letter / Word Identification Test and Curriculum-based measurement (CBM) |
| **Funding source** | Unclear |
| **Coding** | + effects on reading |
| **Notes** |  |

|  | Words to go!: Evaluating a first-grade parent involvement program for “making” words at home |
| --- | --- |
| **Authors** | **Reutzel, R. R., Fawson, P. C., & Smith, J. A.** |
| **Year of publication** | 2006 |
| **Publication type** | Journal article |
| **Methods** | Study design:  Quasi-experimental, non-equivalent groups design.  One school selected for intervention, matched with another school of similar poverty and poor performance, which acted as the control group.  Intention to treat:  No  Duration pre-post:  7 months  Duration follow up:  1 month for qualitative measures |
| **Participants** | Country:  USA  Setting:  Home  Indicated risk: recruited from two high poverty, low performing schools, that qualitied for the Reading Excellence Act because of poor performance and high poverty  N in intervention conditions: 67  N in control conditions: 77  Total N: 144  How many eligible/approached for participation (% of eligible participated= reach): unclear  Mean age: first grade  Gender: not mentioned |
| **Interventions and comparisons** | Condition 1:  Parent involvement program, where parents learn words lessons and received material based on the schools systematic phonics program, to be used at home.  Both intervention group and control group had participated in a 2-year family literacy program through the Reading Excellence Act.  Duration: Three parent workshops the first two months. After that the family received new material weekly for 6-7 months.  Comparison 1: control groups from a matched school  Duration: 7 months |
| **Outcomes** | Reading:  The Systematic Sequential Phonic They Use - Word reading  A word writing (spelling) test  The Systematic Sequential Phonic They Use for beginning Readers of All Ages  The State Core Assessment End-Of-Level test in Language Arts was used as Criterion-references end-of-year reading test |
| **Funding source** | Not mentioned |
| **Coding** | + effects on reading |
| **Notes** |  |

|  | Enhancing the Educational Achievement of At-Risk Youth |
| --- | --- |
| **Authors** | **Schinke, S.P., Cole, K.C., & Poulin, S.R** |
| **Year of publication** | 2000 |
| **Publication type** | Journal article |
| **Methods** | Study design: Quasi experimental with matched controls  Duration pre-post: Unclear  Duration follow up: -  Intention-to-treat: unclear |
| **Participants** | Country: USA  Setting: At after school program (boys and girls club)  Type of risk: indicated risk based on economic disadvantage and living in public housing  N in intervention conditions: unclear  N in control conditions: unclear  Total N: 283, 13.78% attrition  How many eligible/approached for participation (% of eligible participated= reach): unclear  Mean age: 12.3 years (*SD*=1.9)  Gender: 40% female |
| **Interventions and comparisons** | Intervention: Educational enhancement program including weekly structured academic activities after school. Local staff engage youths in discussion, creative writing, leisure reading, homework completion, peer tutoring, board games and other recreational pursuits.  Comparison: No after school program |
| **Outcomes** | - Self-report of engagement and enjoyment with reading, conversation, writing, tutoring, geography, educational board games, and life-enhancement activities. - Teacher report on the same scale as above.   Grades in math, English grammar, composition, reading, spelling, history, science, social studies, geography, and grade point averages |
| **Funding source** | Carnegie Corporation of New York |
| **Coding** | ÷ on reading, math and gpa |
| **Notes** | Positive results, but difference between group not statistically significant. Significant differences on attendance. |

|  | Parents encourage pupils (PEP): An innercity parent involvement reading project |
| --- | --- |
| **Authors** | **Shuck, A., Ulsh, F. & Platt, J. S.** |
| **Year of publication** | 1983 |
| **Publication type** | Journal article |
| **Methods** | Study design: Individually randomized controlled trial  Duration pre-post: Unclear  Duration follow up: -  Intention-to-treat: unclear |
| **Participants** | Country: USA, Pennsylvania  Setting: Intervention delivered in the home  Type of risk: Identified risk. All were behind at least two grade levels in reading, were of average intelligence or above and were placed in a reading resource laboratory.  N in intervention conditions: 75  N in control conditions: 75  Total N: 150  How many eligible/approached for participation (% of eligible participated= reach): unclear  Mean age: Unclear (3-5 grade)  Gender: Unclear |
| **Interventions and comparisons** | Condition 1: Parents were encouraged to tutor their children and help them with their homework, read a book, work on word list or play educational games. Parents monitor, and studentss gets points for work done at home.  Duration: Unclear  Comparison 1: Comparable group of children who did not receive parent tutoring  Duration: Unclear |
| **Outcomes** | Reading: Stanford Achievement Test |
| **Funding source** | Unclear |
| **Coding** | + effects on reading |
| **Notes** | Poor reporting |

|  | Supporting Families in a High-Risk Setting: Proximal Effects of the SAFEChildren Preventive Intervention |
| --- | --- |
| **Authors** | **Tolan, P., Gorman-Smith, D. & Henry, D.** |
| **Year of publication** | 2004 |
| **Publication type** | Journal article |
| **Methods** | Study design: Individually randomized controlled trial  Duration pre-post: 1 year  Duration follow up: 1 year  Intention-to-treat: yes |
| **Participants** | Country: USA, Chicago  Setting: Intervention delivered both at school and in the home  Indicated risk. Low income, high crime rate inner-city neighborhood  N in intervention conditions: Unclear  N in control conditions: Unclear  Total N: 424 families (attrition=23)  How many eligible/approached for participation (% of eligible participated= reach): 507. Reach= 84%  Mean age: Unclear (age range 5-6)  Gender: 49% girls |
| **Interventions and comparisons** | Condition 1: SAFEChildren program with two components; 1) a multiple family group intervention focused on parenting skills, family relationships, parental support and parental involvement, and phonetic based reading tutoring program (from Fast Track program).  Duration: 22 weeks  Comparison 1: Unclear  Duration: Unclear |
| **Outcomes** | Reading: Woodcock Diagnostic Reading Battery total reading composite (Woodcock, 1997) |
| **Funding source** | Unclear |
| **Coding** | + effects on reading |
| **Notes** |  |

|  | The Benefits of Booster Interventions: Evidence from a Family-Focused Prevention Program |
| --- | --- |
| **Authors** | **Tolan, P. H., Gorman-Smith, D., Henry, D. & Schoeny, M.** |
| **Year of publication** | 2009 |
| **Publication type** | Journal article |
| **Methods** | Study design: Individually randomized controlled trial  Duration pre-post: 1 year  Duration follow up: 1 year  Intention-to-treat: yes |
| **Participants** | Country: USA, Chicago  Setting: Intervention delivered in the home  Type of risk: Indicated risk. Fourth graders just prior to the age when delinquency and other related social problems are emerging.  N in intervention conditions: 101  N in control conditions: 95  Total N: 348  How many eligible/approached for participation (% of eligible participated= reach): reach=95%  Mean age: Unclear (fourth graders)  Gender: 54.6% female, 45.4% male. 42.6% were African American, 54.8% Latino and 2.4% other ethnicity. |
| **Interventions and comparisons** | Condition 1: Booster intervention of the SAFEChildren program with two components; 1) a multiple family group intervention focused on parenting skills, family relationships, parental support and parental involvement, and phonetic based reading tutoring program (from Fast Track program).  Duration: 20 sessions  Comparison 1: Received initial intervention, but no booster intervention.  Duration: Unclear |
| **Outcomes** | Reading: Iowa Test of Basic Skills, California Achievement Test |
| **Funding source** | Unclear |
| **Coding** | + effects on reading |
| **Notes** |  |

|  | Peer Tutoring with or without Home-based Reinforcement, For Reading Remediation |
| --- | --- |
| **Authors** | Trovato, J. & Bucher, B. |
| **Year of publication** | 1980 |
| **Publication type** | Journal article |
| **Methods** | Study design: Individually randomized controlled trial  Duration pre-post: 15 weeks  Duration follow up: -  Intention-to-treat: no |
| **Participants** | Country: Canada, Ontario  Setting: Intervention in group 1 delivered in school (PT), intervention in group 2 delivered both in the home and at school (PT+HB)  Type of risk: Identified risk. Deficient in reading skills  N in intervention conditions: Group 1 (PT) = 23, Group 2 (PT+HB) = 23  N in control conditions: 23  Total N: 90 (attrition=21)  How many eligible/approached for participation (% of eligible participated= reach): unclear  Mean age: Group 1 (PT) = 9.1, Group 2 (PT+HB) = 9.0 and Control = 9.1  Gender: Unclear |
| **Interventions and comparisons** | Condition 1: Peer Tutoring Only (PT)  Duration: 30 min for 15 weeks  Condition 2: Peer Tutoring (PT) plus Home-based reinforcement (HB). Contingency management; using point earned for accurate oral reading and comprehension could be exchanged for rewards in the home.  Duration: 30 min for 15 weeks  Comparison 1: Some children were enrolled in in-school remedial reading programs. No attempt was made to control the instruction received in this group.  Duration: 15 weeks |
| **Outcomes** | Reading: Spache Diagnostic Reading Scale and the test of reading ability provided by the SRA Starter Stories |
| **Funding source** | Unclear |
| **Coding** | + effects on reading |
| **Notes** |  |

|  | An Evaluation of Preventive Tutoring Programs for Transfer Students |
| --- | --- |
| **Authors** | **Weine, A.M., Kurasaki, K.S., Jason, L.A., Danner, K.E., & Johnson, J.H** |
| **Year of publication** | 1993 |
| **Publication type** | Journal article |
| **Methods** | Study design: Cluster (schools) randomized controlled trial  Intention to treat: no  Duration pre-post: 8-10 months  Duration follow up: 8-10 months (separate article; Jason et al., 1993) |
| **Participants** | Country: USA  Setting: Urban parochial elementary schools. Interventions delivered at home or at school.  Identified risk: Evidencing lag in at least one area of standardized achievement test  Indicated risk: Low SES, having experienced two or more life stress events based on parental reports  N in intervention conditions: Group 1= 48 (+ 11 dropouts), group 2= 48 (+ 10 dropouts)  N in control conditions: 51 (+6 dropouts)  Total N: 147 (with dropouts= 174)  How many eligible/approached for participation (% of eligible participated= reach): 174  Mean age: not specified. Third graders: 40%, fourth graders: 30% and fifth graders: 30%  Gender: 48% girls, 52% boys |
| **Interventions and comparisons** | School-only tutoring: Trained paraprofessionals providing 40-60 min tutoring twice a week. Tutors used direct instruction (e.g. model-lead-test technique, positive reinforcement, corrective feedback) to tutor reading comprehension, phonics, spelling and arithmetic. Weekly monitoring and feedback included.  *Duration:* Twice a week for eight months  Home-plus-School Tutoring: Same as school-tutoring + parent tutoring at home. Parents received a one-hour training session in direct instruction, parental involvement, corrective feedback, and contingency management. Homework schedule and goal setting was used, and a behavioral contract. Parents received ongoing support from authors.  *Duration:* School tutoring twice a week for eight months, dose of parent tutoring not reported  Control condition: Unclear |
| **Outcomes** | Reading:   - Wide Range Achievement Test Revised (Jastak & Wilkinson, 1984). - Reading and spelling grade reports   Math:   - Wide Range Achievement Test Revised (Jastak & Wilkinson, 1984). - Arithmetic grade report |
| **Funding source** | National Institute of Mental Health, grant number MH40851095 |
| **Coding** | + effects on reading (math coded in Jason et al.,, 1993, same study) |
| **Notes** | Limited reporting |

|  | Parent Participation Reading Clinic – A Research-Demonstration Project. Final Report. |
| --- | --- |
| **Authors** | **Wise, J. H.** |
| **Year of publication** | 1972 |
| **Publication type** | Report |
| **Methods** | Study design: Pre-post test with matched controls  Duration pre-post: six to eight months  Duration follow up: -  Intention-to-treat: no attrition or missing |
| **Participants** | Country: USA, Washington  Setting: Intervention delivered in the home  Type of risk: Indicated risk. Children of low income families  N in intervention conditions: 19  N in control conditions: 19  Total N: 38  How many eligible/approached for participation (% of eligible participated= reach): unclear  Mean age: 8.45 (range 7-12)  Gender: Experimental group = 13 boys, 6 girls  Control group = 10 boys, 9 girls |
| **Interventions and comparisons** | Condition 1: Home-instructors (child’s parents or older sibling) assisting in the teaching of reading to elementary school children.  Duration: Eight months  Comparison 1: Unclear  Duration: Eight months |
| **Outcomes** | Reading: California Test of Basic Skills, Dolch Word List, Peabody Picture Vocabulary Test and Wide Range Achievement Test |
| **Funding source** | Unclear |
| **Coding** | + effects on reading |
| **Notes** | Poor reporting |

|  | No Child Left Behind: An Assessment of an Afeter-School Porgram on Academic Performance Among Low-Income, At-Risk Students |
| --- | --- |
| **Authors** | **Zosky, D. L. & Crawford, L. A.** |
| **Year of publication** | 2003 |
| **Publication type** | Journal article |
| **Methods** | Study design: Quasi-experimental design, non-equivalent control group  Duration pre-post: 1 year (grades given each academic quarter)  Duration follow up: 2 years  Intention-to-treat: no |
| **Participants** | Country: USA, Midwestern city  Setting: Intervention delivered after school. Unclear if delivered in after school center or in the school.  Type of risk: Indicated risk. Low-income, at-risk fourth grade students attending a public school in an urban setting  N in intervention conditions: 14  N in control conditions: 63  Total N: 77 (attrition=6)  How many eligible/approached for participation (% of eligible participated= reach): unclear  Mean age: Unclear (fourth graders)  Gender: Unclear |
| **Interventions and comparisons** | Condition 1: After school program delivered by social service workers and volunteers consisting of supervising homework completion and reinforcing academic performance with rewards.  Duration: 6 hours a week for two years  Comparison 1: Not in the program  Duration: Unclear |
| **Outcomes** | Reading: Grades  Math: Grades |
| **Funding source** | Not stated |
| **Coding** | + Intervention significantly effective on math, language arts, and GPA grades  ÷ Not significantly effective on reading grades |
| **Notes** |  |

*reach: percentage of invited subjects agreeing to participate in the study (Glasgow et al., 2006)

Glasgow, R. E., Klesges, L. M., Dzewaltowski, D. A., Estabrooks, P. A., & Vogt, T. M. (2006). Evaluating the impact of health promotion programs: using the RE-AIM framework to form summary measures for decision making involving complex issues. *Health education research*, *21*(5), 688-69

## Risk of bias of included studies

Summarized risk of bias in included studies assessed using Cochrane Risk of Bias Tool


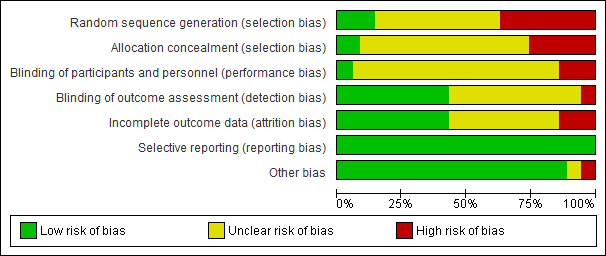


Risk of bias of each included study


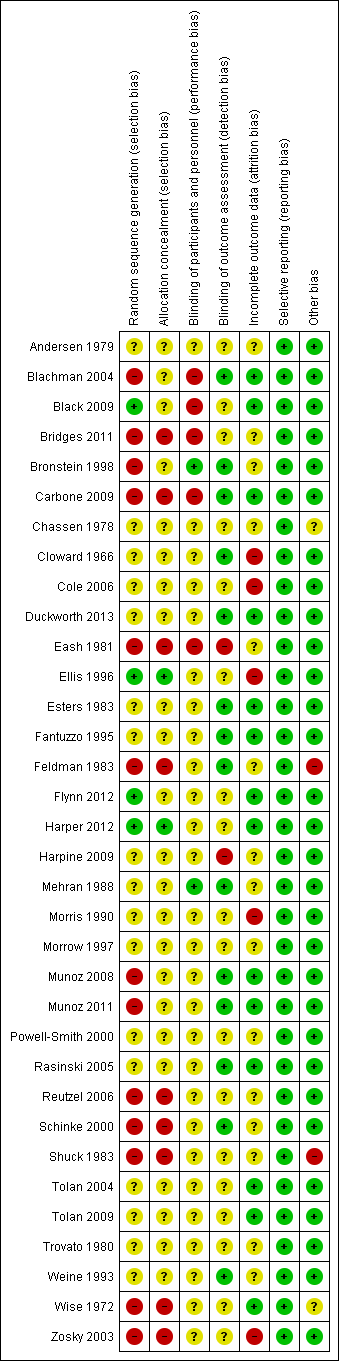


## Reference list included studies

Andersen, B. L., Licht, B. G., Ullmann, R. K., Buck, S. T., & Redd, W. H. (1979). Paraprofessional reading tutors: Assessment of the Edmark Reading Program and flexible teaching. *American journal of community psychology*, *7*(6), 689-699.

Blachman, B. A., Schatschneider, C., Fletcher, J. M., Francis, D. J., Clonan, S. M., Shaywitz, B. A., & Shaywitz, S. E. (2004). Effects of Intensive Reading Remediation for Second and Third Graders and a 1-Year Follow-Up. *Journal of Educational Psychology*, *96*(3), 444.

Black, A. R., Somers, M. A., Doolittle, F., Unterman, R., & Grossman, J. B. (2009). The Evaluation of Enhanced Academic Instruction in After-School Programs: Final Report. NCEE 2009-4077. *National Center for Education Evaluation and Regional Assistance*.

Bridges, C. (2011). *The correlation between after school tutoring and reading scores of at- risk third graders* (Doctoral dissertation, Walden University).

Bronstein, P., Duncan, P., Clauson, J., Abrams, C. L., Yannett, N., Ginsburg, G., et al. (1998). Prevencaspiting middle school adjustment problems for children from lower- income families: A program for Aware Parenting. *Journal of Applied Developmental Psychology*.

Carbone, P. M. (2009). *The Effects of an After-School Tutoring Program on the Pennsylvania System of School Assessment* (Doctoral dissertation, Youngstown State University).

Chassen, L. R. (1978). *The effects of parent training in behavior modification and transactional analysis on the reading achievement, school attitudes, and self-concept of remedial readers in a remedial reading program* (Doctoral dissertation, ProQuest Information and Learning).

Cid, A. (2014). Giving a second chance: an after-school programme in a shanty town interacted with parent type: lessons from a randomized trial. *Educational Research and Evaluation*, *20*(5), 348-365.

Cloward, R. D. (1967). Studies in tutoring. *The Journal of Experimental Edu.*, *36*(1), 14-25.

Cole, J. M., & Hilliard, V. R. (2006). The effects of web-based reading curriculum on children's reading performance and motivation. *Journal of Educational Computing Research*, *34*(4), 353-380.

Duckworth, A. L., Kirby, T. A., Gollwitzer, A., & Oettingen, G. (2013). From fantasy to action: Mental contrasting with implementation intentions (MCII) improves academic performance in children. *Social Psychological and Personality Science*, *4*(6), 745-753.

Eash, M. J. (1981). Evaluation of Multiple Outcomes in the Child Parent Center Compensatory Education Program: Year II.

Ellis, M. G. (1996). Parent-Child Reading Programs: Involving Parents in the Reading Intervention Process: Summary of Dissertation Research.

Esters, P., & Levant, R. F. (1983). The effects of two parent counseling programs on rural low-achieving children. *The School Counselor*, *31*(2), 159-166.

Fantuzzo, J. W., Davis, G. Y., & Ginsburg, M. D. (1995). Effects of parent involvement in isolation or in combination with peer tutoring on student self-concept and mathematics achievement. *Journal of Educational Psychology*, *87*(2), 272.

Feldman. (1983). Parent and paraprofessional tutoring of academically-delayed and learning disabled children. *Canada's Mental Health,* *31*(1), 17-20.

Flynn, R. J., Marquis, R. A., Paquet, M. P., Peeke, L. M., & Aubry, T. D. (2012). Effects of individual direct-instruction tutoring on foster children's academic skills: A randomized trial. *Children and Youth Services Review*, *34*(6), 1183-1189.

Harper, J., & Schmidt, F. (2012). Preliminary effects of a group-based tutoring program for children in long-term foster care. *Children and Youth Services Review*, *34*(6), 1176- 1182.

Mehran, M., & White, K. R. (1988). Parent tutoring as a supplement to compensatory education for first-grade children. *Remedial and Special Education*, *9*(3), 35-41.

Morrow, L. M., & Young, J. (1997). A family literacy program connecting school and home: Effects on attitude, motivation, and literacy achievement. *Journal of Educational Psychology*, *89*(4), 736.

Morris, D., Shaw, B., & Perney, J. (1991). Helping low readers in grades 2 and 3: An after- school volunteer tutoring program. *The Elementary School Journal*, *91*(2), 133-150.

Muñoz, M. A., Chang, F., & Ross, S. M. (2012). No child left behind and tutoring in reading and mathematics: Impact of supplemental educational services on large scale assessment. *Journal of Education for Students Placed at Risk*, *17*(3), 186-200.

Muñoz, M. A., Potter, A. P., & Ross, S. M. (2008). Supplemental educational services as a consequence of the NCLB legislation: Evaluating its impact on student achievement in a large urban district. *Journal of Education for Students Placed at Risk*, *13*(1), 1-25.

Powell-Smith, K. A., Stoner, G., Shinn, M. R., & Good III, R. H. (2000). Parent Tutoring in Reading Using Literature and Curriculum Materials: Impact on Student Reading Achievement. *School Psychology Review*, *29*(1).

Rasinski, T., & Stevenson, B. (2005). The effects of fast start reading: a fluency-based home involvement reading program, on the reading achievement of beginning readers. *Reading Psychology*, *26*(2), 109-125.

Reutzel, D. R., Fawson, P. C., & Smith, J. A. (2005). Words to go!: Evaluating a first‐grade parent involvement program for “making” words at home. *Literacy Research and Instruction*, *45*(2), 119-159.

Schinke, S. P., Cole, K. C., & Poulin, S. R. (2000). Enhancing the educational achievement of at-risk youth. *Prevention Science*, *1*(1), 51-60.

Shuck, A., Ulsh, F., & Platt, J. S. (1983). Parents encourage pupils (PEP): An innercity parent involvement reading project. *The Reading Teacher*, *36*(6), 524-528.

Tolan, P., Gorman-Smith, D., & Henry, D. (2004). Supporting families in a high-risk setting: proximal effects of the SAFEChildren preventive intervention. *Journal of consulting and clinical psychology*, *72*(5), 855.

Tolan, P. H., Gorman-Smith, D., Henry, D., & Schoeny, M. (2009). The benefits of booster interventions: Evidence from a family-focused prevention program. *Prevention Science*, *10*(4), 287-297.

Trovato, J., & Bucher, B. (1980). Peer tutoring with or without home‐based reinforcement, for reading remediation. *Journal of Applied Behavior Analysis*, *13*(1), 129-141.

Wise, J. H. (1972). Parent Participation Reading Clinic--A Research-Demonstration Project. Final Report.

Zosky, D. L., & Crawford, L. A. (2003). No child left behind: An assessment of an after- school program on academic performance among low-income, at-risk students. *School Social Work Journal*, *27*(2), 18-31

## Excluded studies

| Excluded studies | Reasons |
| --- | --- |
| Effectiveness of Counseling and Guidance Techniques for Elementary School Students. Final Report. North Dakota Univ., Grand Forks. | Wrong outcomes |
| *Palama Settlement: Effect of the Academic-Athletic Program on Future School Performance*. (1974). Retrieved from http://ovidsp.ovid.com/ovidweb.cgi?T=JS&CSC=Y&NEWS=N&PAGE=fulltext&D=eric1&AN=ED126207 | Relevant, but insufficient data provided |
| Pupil Reading Achievement in the Homework Helper Program. Preliminary Report. (1964): Mobilization for Youth, Inc., New York, NY. | Couldn’t retrieve report |
| Strengthening Reading Services through Increasing Provisions for Elementary Reading Centers. (1967): Milwaukee Public Schools, WI. Div. of Curriculum and Instruction. | Wrong setting |
| Early Reading Assistance: A Reading Tutorial Program. (1968): Program for Action by Citizens in Education, Cleveland, OH. | Wrong setting |
| After-School Study Centers, New York City. Elementary Program in Compensatory Education 2. (1969): American Institutes for Research in the Behavioral Sciences, Palo Alto, CA. | Wrong setting |
| Homework Helper Program, New York City. It Works. (1969). | Wrong intervention |
| The Impact of Head Start: An Evaluation of the Effects of Head Start on Children's Cognitive and Affective Development. (Executive Summary). (1969): Westinghouse Learning Corp., New York, NY., Ohio Univ., Athens. | Wrong study design |
| Speech and Language Development Program Milwaukee, Wisconsin. Elementary Program in Compensatory Education 2, It Works. (1969). | Couldn’t retrieve report |
| Follow-Up Study for Retention of Skills Improved by Special Remedial Reading Instruction at the Reading Center During 1968-69. (1970): Broward County School Board, Fort Lauderdale, FL. | Wrong setting |
| Evaluation of the Reading Center's Remedial Program for the 1970-71 School Year. (1971): Broward County School Board, Fort Lauderdale, FL. | Wrong study design |
| Evaluation of Project Upswing, Interim Report. (1972): Operations Research, Inc., Silver Spring, MD. | Inadequate study-info |
| Model Programs Compensatory Education: More Effective Schools Program, New York, New York. (1972): American Institutes for Research in the Behavioral Sciences, Palo Alto, CA. | Wrong study design |
| Title III Project Annual Report, 1971-72: Dale Avenue Early Childhood Education Project. Research Bulletin, Volume 2, Number 4, 1971-72. (1972): Paterson Board of Education, NJ. | Couldn’t retrieve report |
| Newark School District, Part One -- Regular School Year. ESEA Title I Program, 1972-1973 School Year. (1973): Communication Technology Corp., Marlton, NJ., Newark Board of Education, NJ. | Wrong setting |
| The Family Education Program at Intermediate School 55. A Report to the Salgo-Noren Foundation on the Operation and Results of the Family Education Program, March, 1974, through June, 1975. (1975): Bedford-Stuyvesant Restoration Corp., Brooklyn, NY. | Wrong population |
| Project S.T.E.P.: Seniors Tutor for Educational Progress. Annual Evaluation Report, July 1974 - June 1975. (1975): Easton-Redding Regional School District 9, CT. | Couldn’t retrieve report |
| Basic Skills Learning Centers Evaluation. Final Report 1 October 1976 - 30 September 1979. (1976): California Univ., Los Angeles. Center for the Study of Evaluation. | Couldn’t retrieve report |
| Higher Horizons 100, 1978-1979; Hartford Moves Ahead: An Evaluative Report. (1979): Hartford Public Schools, CT. | Couldn’t retrieve report |
| Nonpublic School Programs: Clinical and Guidance Services. ESEA Title I. Final Evaluation Report, 1979-1980. (1980): New York City Board of Education, Brooklyn, NY. Office of Educational Evaluation. | Wrong study design |
| Title I, ESEA, Compensatory Mathematics Program, Submitted to Joint Dissemination Review Panel (N.I.E. and U.S.O.E.). (1980): Des Moines Public Schools, IA. | Couldn’t retrieve report |
| Title I Institutional Facilities Program. Final Evaluation Report, 1980-81. (1981): New York City Board of Education, Brooklyn, NY. Office of Educational Evaluation. | Wrong study design |
| Early Childhood Education Program--Grades 1-6, FY '81. Elementary and Secondary Education Act, Title I. Evaluation Report, 1980-81. (1982): Baltimore City Public Schools, MD. | Inadequate study-info |
| Early Childhood Education Program--Grades 5-9, FY '81. Elementary and Secondary Education Act, Title I. Evaluation Report, 1980-81. (1982): Baltimore City Public Schools, MD. | Wrong study design |
| Elementary and Secondary Programs, ESEA Title I. Final Report, 1982. (1982): Vazquez-Nuttall Associates, Inc., Newton, MA. | Couldn’t retrieve report |
| Title I Institutionalized Facilities Program. O.E.E. Evaluation Report, 1981-82. (1982): New York City Board of Education, Brooklyn, NY. Office of Educational Evaluation. | Wrong study design |
| Disadvantaged Pupil Program Fund. Twelve Final Evaluation Reports, 1983-1984. (1984): Cleveland Public Schools, OH. Dept. of Research and Analysis. | Couldn’t retrieve report |
| School Community Education Program in New York City, 1984-85. Volume IV: OEA Final Evaluation Report. (1986): New York City Board of Education, Brooklyn. Office of Educational Assessment. | Couldn’t retrieve report |
| Responsive Early Childhood Education Program (RECEP). (1987): Goldsboro City Schools, NC. | Wrong setting |
| Chapter 1 in North Carolina, 1986-1987. (1988): North Carolina State Dept. of Public Instruction, Raleigh. Div. of Support Programs. | Wrong study design |
| Paired Learning: Tutoring by Non-Teachers. Incorporating "The Paired Reading Bulletin" No. 5. (1989). | Couldn’t retrieve article |
| Chapter 1 Corrective Reading Program, 1988-89. Evaluation Section Report. (1990): New York City Board of Education, Brooklyn, NY. Office of Research, Evaluation, and Assessment. | Wrong study design |
| The Des Moines Plan for Student Success. Focus on Program Evaluation. (1992): Des Moines Public Schools, IA. | Wrong setting |
| Reading Recovery Program 1991-92. Compensatory Education Product Evaluation. (1992): Saginaw Public Schools, MI. Dept. of Evaluation Services. | Wrong setting |
| WCPSS Reading Recovery 1990-94. Evaluation Report. E&R Report No. 95.09A. (1995): Wake County Public Schools System, Raleigh, NC. Dept. of Evaluation and Research. | Wrong setting |
| Reading Recovery Program 1996-97 Evaluation Report. Pamphlet 98-C-002. (1998): Department of Defense Education Activity, Arlington, VA. | Wrong setting |
| GEAR UP Austin: Impacting Lives Project, 2000-2001 Evaluation. (2001): Austin Independent School District, TX. Office of Program Evaluation. | Wrong setting |
| Caring School Community[TM] (Formerly, the Child Development Project). What Works Clearinghouse Intervention Report. (2006). | Wrong setting |
| National Assessment of Title I. Final Report. Summary of Key Findings. NCEE 2008-4014. (2007). | Wrong study design |
| Poverty and Early Childhood Intervention. FPG Snapshot #42. (2007). | Wrong population |
| Start Making a Reader Today[R] (SMART[R]). What Works Clearinghouse Intervention Report. (2007). | Wrong setting |
| Afterschool Programs: Making a Difference in America's Communities by Improving Academic Achievement, Keeping Kids Safe and Helping Working Families. (2008). | Inadequate study-info |
| Effective Programs for Struggling Readers: A Best-Evidence Synthesis. [Educator's Summary]. (2009). | Review/synthesis |
| STEM Learning in Afterschool: An Analysis of Impact and Outcomes. (2011). | Review/synthesis |
| WWC Review of the Report "Improving At-Risk Learners' Understanding of Fractions." What Works Clearinghouse Single Study Review. (2013). | Wrong setting |
| Fast Track: Elementary School. What Works Clearinghouse Intervention Report. (2014). | Wrong setting |
| Abbott, S. P. (1998). The effect of phonological decoding and structural analysis training on the reading achievement of delayed readers in the intermediate grades. *59*(6-A), 1899. | Couldn’t retrieve article |
| AbuSeileek, A. (2013). Using track changes and word processor to provide corrective feedback to learners in writing. *29*(4), 319-333. doi: <http://dx.doi.org/10.1111/jcal.12004> | Wrong setting |
| Adachi, P. J. C., & Willoughby, T. (2013). More Than Just Fun and Games: The Longitudinal Relationships Between Strategic Video Games, Self-Reported Problem Solving Skills, and Academic Grades. *42*(7), 1041-1052. doi: 10.1007/s10964-013-9913-9 | Wrong population |
| Adams, C. L. (2012). The effects of a remedial math intervention on standardized test scores in Georgia middle schools. *72*(10-A), 3723. | Wrong setting |
| Aeby, V. G., Thyer, B. A., & Carpenter-Aeby, T. (1999). Comparing Outcomes of an Alternative School Program Offered with and without Intensive Family Involvement. | Wrong population |
| Aghababaei, S., Malekpour, M., & Abedi, A. (2012). Effectiveness of executive functions training on academic performance of children with spelling learning disability. *14*(2[54]; 54), 63-72. | Unable to translate |
| Ajwani, S. (2008). The success of educational interventions in grades three and five in improving academic progress. *68*(10-A), 4145. | Couldn’t retrieve full article |
| Akers, C. W. (1976). Effects of a Supplemental Program in Structural Analysis upon Reading Scores of Children Deficient in Grammatic Closure. | Couldn’t retrieve full dissertation |
| Al Otaiba, S., & Lake, V. E. (2007). Preparing special educators to teach reading and use curriculum-based assessments. *20*(6), 591-617. doi: http://dx.doi.org/10.1007/s11145-007-9056-z | Wrong setting |
| Alexander, A. S. (2001). Three-year follow-up and parent perceptions of preventive school- and home-based behavioral family therapy for high-risk adolescents. *62*(1-B), 534. | Couldn’t retrieve full dissertation |
| Alexander, P. L. (1992). Intense Early Intervention for Young Readers at Risk: Edmonton Public Schools (Alberta). | Wrong setting |
| Allen, A., & Chavkin, N. F. (2004). New Evidence that Tutoring with Community Volunteers Can Help Middle School Students Improve Their Academic Achievement. *14*(2), 7-18. | Wrong setting |
| Allen, C. S. (2010). A prospective longitudinal investigation of effects of nonparental social support on early adolescents' academic achievement and academic outcomes. *70*(12-B), 7878. | Wrong study design |
| Alloway, T. (2012). Can interactive working memory training improve learning? *, 23*(3), 197-207. | Wrong population |
| Alloway, T. P., Bibile, V., & Lau, G. (2013). Computerized working memory training: Can it lead to gains in cognitive skills in students? *29*(3), 632-638.  doi: <http://dx.doi.org/10.1016/j.chb.2012.10.023> | Wrong intervention |
| Almeida, E. (1979). A program of collaboration of parents and teachers to stimulate the motivation and learning of sixth-grade children. *5*(1), 437-457. | Unable to translate |
| Amble, B. R., & Muehl, S. (1966). Perceptual span training and reading achievement of school children. *57*(4), 192-206. doi: <http://dx.doi.org/10.1037/h0023606>. | Wrong setting |
| Anderson, B. (2002). Colorado Even Start Progress Report, 2001-2002: Colorado State Dept. of Education, Denver. | Wrong study design |
| Anderson, B. (2006). Colorado Even Start 2005-2006 Progress Report. | Inadequate study-info |
| Andersson, K. E. (1982). The effect of cognitive-behavioral group intervention on academic task performance. *42*(9-B), 3814. | Couldn’t retrieve article |
| Anesko, K. M., & O'Leary, S. G. (1982). The effectiveness of brief parent training for the management of children's homework problems. *4*(2-3), 113-126. doi: <http://dx.doi.org/10.1300/J019v04n02_13> | Wrong intervention |
| Ang, S. Y., Lee, K., Cheam, F., Poon, K., & Koh, J. (2015). Updating and working memory training: Immediate improvement, long-term maintenance, and generalisability to non-trained tasks. *4*(2), 121-128. doi: <http://dx.doi.org/10.1016/j.jarmac.2015.03.001>. | Wrong setting |
| Arbreton, A., Sheldon, J., Bradshaw, M., & Goldsmith, J. (2008). Advancing Achievement: Findings from an Independent Evaluation of a Major After-School Initiative. | Wrong study design |
| Arnold, J. M. (2013). The Parents as Teachers Program in Missouri and the Resulting Difference in Academic Effects for Fifth- and Sixth-Grade Students. | Wrong population |
| Arnold, L., Barnebey, N., McManus, J., Smeltzer, D. J., Conrad, A., Winer, G., & Desgranges, L. (1977). Prevention by specific perceptual remediation for vulnerable first-graders: Controlled study and follow-up of lasting effects. *34*(11), 1279-1294. doi: <http://dx.doi.org/10.1001/archpsyc.1977.01770230021001>. | Wrong setting |
| Arriaga, X. B., & Longoria, Z. N. (2011). Implementation Intentions Increase Parent-Teacher Communication Among Latinos. *33*(4), 365-373. doi: 10.1080/01973533.2011.614142. | Wrong population |
| Ashbaugh, J. A. (2009). A Study of the Effects of Parental Involvement on the Success of Students on a High-Stakes State Examination. | Wront study design |
| Ashby, K. (2014). Initial effects of wilson reading system on student reading and spelling achievement. *74*(12-A(E)), No Pagination Specified. | Wrong setting |
| Atkins, M. S., Shernoff, E. S., Frazier, S. L., Schoenwald, S. K., Cappella, E., Marinez-Lora, A., Bhaumik, D. (2015). Redesigning community mental health services for urban children: Supporting schooling to promote mental health. *Journal of Consulting and Clinical Psychology, 83*(5), 839-852. doi: <http://dx.doi.org/10.1037/a0039661>. | Wrong setting |
| Ayasse, R. H. (1995). Addressing the Needs of Foster Children: The Foster Youth Services Program. *17*(4), 207-216. | Review/synthesis |
| Bacon, L., Chovelak, C., & Wanic, A. (1998). Instructional Techniques To Improve Homework Completion with Sixth Grade and Spanish I Students. | Unable to retrieve study information |
| Baenen, N., Bernholc, A., Dulaney, C., & Banks, K. (1997). Reading recovery: Long-term progress after three cohorts. *2*(2), 161-181. doi: <http://dx.doi.org/10.1207/s15327671espr0202_6>. | Wrong setting |
| Bakall, L., & et al. (1991). Evaluation of the 1989 and 1990 Reading Improvement Program. Illinois Initiatives for Educational Reform. Research, Evaluation, & Planning Report: Chicago Board of Education, IL. Dept. of Research and Evaluation. | Wrong study design |
| Baker, L. (2003). The role of parents in motivating struggling readers. *19*(1), 87-106. doi: <http://dx.doi.org/10.1080/10573560308207>. | Wrong study design |
| Baker, S., Gersten, R., & Keating, T. (2000). When Less May Be More: A 2-Year Longitudinal Evaluation of a Volunteer Tutoring Program Requiring Minimal Training. *35*(4), 494-519. | Wrong setting |
| Banerjee, A., Cole, S., Duflo, E., & Linden, L. (2005). Remedying Education: Evidence from Two Randomized Experiments in India. NBER Working Paper No. 11904. | Wrong setting |
| Barnett, D. (1980). *Attitudinal Change through a Parent Education Program*. Retrieved from http://ovidsp.ovid.com/ovidweb.cgi?T=JS&CSC=Y&NEWS=N&PAGE=fulltext&D=eric1&AN=ED188156 | Relevant, but insufficient data provided |
| Bass, C. K. (2001). Effects of a culturally relevant intervention on the academic achievement of African American adolescent males. *61*(8-A), 3064. | Unable to retrieve full dissertation |
| Bass, G., Ries, R., & Sharpe, W. (1986). Teaching basic skills through microcomputer assisted instruction. *2*(2), 207-219. doi: <http://dx.doi.org/10.2190/KEAN-RWUX-7BL2-FP3V>. | Wrong setting |
| Becher, R. M. (1984). Parent Involvement: A Review of Research and Principles of Successful Practice: ERIC Clearinghouse on Elementary and Early Childhood Education, Urbana, IL. | Review/synthesis |
| Beck, R. J. (2012). Effects of Group Parent-Training with Online Parent-Teacher Communication on the Homework Performance of Elementary School Students. | Unable to retrieve full dissertation |
| Beck, R. J. (2013). *Effects of group parent-training with online parent-teacher communication on the homework performance of elementary school students.* (74), ProQuest Information & Learning, US. Retrieved from <http://ovidsp.ovid.com/ovidweb.cgi?T=JS&CSC=Y&NEWS=N&PAGE=fulltext&D=psyc10&AN=2013-99210-566> | Unable to retrieve article |
| Beckett, M. K. (2008). Current-Generation Youth Programs: What Works, What Doesn't, and at What Cost? Occasional Paper. | Review/synthesis |
| Beckum, L. C. (1973). The Effect of Counseling and Reinforcement on Behaviors Important to the Improvement of Academic Self-Concept. Technical Report Number 38: Stanford Univ., CA. Stanford Center for Research and Development in Teaching. | Wrong setting |
| Bellert, A. (2009). LDA Student Award Winner, 2008: Narrowing the Gap--A Report on the "Quicksmart" Mathematics Intervention. *14*(2), 171-183. | Wrong population |
| Berger, C., Milicic, N., Alcalay, L., & Torretti, A. (2014). Learning and wellbeing program for third and fourth grade students: Impact description and evaluation. *46*(3), 169-177. | Language |
| Bergin, D. A., & et al. (1992). An Afterschool Intervention Program for Educationally Disadvantaged Young Children. 24(3), 203-217. | Relevant, but insufficient data provided |
| Berkowitz, T., Schaeffer, M. W., Maloney, E. A., Peterson, L., Gregor, C., Levine, S. C., & Beilock, S. L. (2015). Math at home adds up to achievement in school. *350*(6257), 196-198. doi: <http://dx.doi.org/10.1126/science.aac7427>. | Wrong population |
| Berninger, V. W., Abbott, R. D., Zook, D., Ogier, S., Lemos-Britton, Z., & Brooksher, R. (1999). Early intervention for reading disabilities: teaching the alphabet principle in a connectionist framework. *32*(6), 491-503. | Wrong setting |
| Bilski-Cohen, R., & Melnik, N. (1974). The Use of Creative Movement for Promoting the Development of Concept Formation and Intellectual Ability in Young Culturally Disadvantaged Children. Final Report: Hebrew Univ. of Jerusalem (Israel). | Unable to retrieve report |
| Birmingham, J., Pechman, E. M., Russell, C. A., & Mielke, M. (2005). Shared Features of High-Performing After-School Programs: A Follow-Up to the TASC Evaluation. | Review/synthesis |
| Blachman, B. A., Schatschneider, C., Fletcher, J. M., Murray, M. S., Munger, K. A., & Vaughn, M. G. (2014). Intensive Reading Remediation in Grade 2 or 3: Are There Effects a Decade Later? *Journal of Educational Psychology, 106*(1), 46-57. | Dual publication |
| Blackmer, M. Z. (1987). Increasing the Awareness of Critical Thinking Skills through a Program of Parent Involvement. | Wrong outcomes |
| Boncana, M. (2011). Partnership, student achievement, and parental involvement in a Utah elementary: Multilevel growth curve and critical interpretive analyses. *71*(9-A), 3112. | Wrong population |
| Bos, J. M., & Fellerath, V. (1997). LEAP: Ohio's Welfare Initiative To Improve School Attendance among Teenage Parents. Ohio's Learning, Earning, and Parenting Program. Final Report: Manpower Demonstration Research Corp., New York, NY. | Wrong population |
| Bradley, K. L. (2002). The effects of the Help One Student to Succeed (HOSTS) program on the reading achievement of at-risk 4th and 5th grade elementary students. *63*(2-A), 501. | Unable to retrive full dissertation |
| Brenner, D., Jayroe, T., & Boutwell, A. (2003). Building on the Strengths of Families: The Promising Readers Program. *80*(4), 275-283. | Wrong study design |
| Brodsky, S., & et al. (1994). *An Urban Family Math Collaborative*. Retrieved from http://ovidsp.ovid.com/ovidweb.cgi?T=JS&CSC=Y&NEWS=N&PAGE=fulltext&D=eric3&AN=ED379154 | Relevant, but lacks sufficient intervention information |
| Brown, D. M., Fuqua, J. W., & Otts, D. A. (1986). Helping reluctant readers "stick" to it. *21*(5), 599-604. | Wrong study design |
| Bryan, T., & Burstein, K. (2004). Improving homework completion and academic performance: Lessons from special education. *43*(3), 213-219. doi: 10.1207/s15430421tip4303_7. | Review/synthesis |
| Burns, G. L., & Kondrick, P. A. (1998). Psychological Behaviorism's Reading Therapy Program: Parents as Reading Therapists for Their Children's Reading Disability. *31*(3), 278-285. | Wrong population |
| Burns, M. K., Senesac, B. J., & Silberglitt, B. (2008). Longitudinal Effect of a Volunteer Tutoring Program on Reading Skills of Students Identified as At-Risk for Reading Failure: A Two-Year Follow-Up Study. *47*(1), 27-37. | Wrong setting |
| Burns, M. K., Senesac, B. V., & Symington, T. (2004). The Effectiveness of the HOSTS Program in Improving the Reading Achievement of Children At-Risk for Reading Failure. *43*(2), 87-103. | Wrong setting |
| Butler, S. R. (1991). Reading program-remedial, integrated, and innovative. *41*, 119-127. doi: <http://dx.doi.org/10.1007/BF02648081>. | Wrong study design |
| Cahill, J. (1981). Towards model refinement in compensatory education: Comparison of intervention programs and paraprofessional screening measures. *9*(6), 731-749. doi: <http://dx.doi.org/10.1007/BF00896252>. | Unable to retrieve full dissertation |
| Cairney, T. H., & Munsie, L. (1992). Breaking Down the Barriers: Parents as Community Tutors in Literacy. | Inadequate study-info |
| Callahan, K., Rademacher, J. A., & Hildreth, B. L. (1998). The Effect of Parent Participation in Strategies To Improve the Homework Performance of Students Who Are At Risk. *19*(3), 131-141. | Wrong setting |
| Campbell, F. A., Pungello, E. P., Burchinal, M., Kainz, K., Pan, Y., Wasik, B. H., . . . Ramey, C. T. (1033). Adult Outcomes as a Function of an Early Childhood Educational Program: An Abecedarian Project Follow-Up. *48*(4), 1033-1043. | Wrong population |
| Cavanaugh, C., Gillan, K. J., Bosnick, J., Hess, M., & Scott, H. (2008). Effectiveness of interactive online algebra learning tools. *38*(1), 67-95. doi: <http://dx.doi.org/10.2190/EC.38.1.d>. | Wrong setting |
| Center, Y., & et al. (1995). An Evaluation of Reading Recovery. *30*(2), 240-263. | Wrong setting |
| Center, Y., Wheldall, K., Freeman, L., Outhred, L., & McNaught, M. (1995). An evaluation of reading recovery. *30*(2), 240-263. doi: <http://dx.doi.org/10.2307/748034>. | Wrong setting |
| Chamberlin, M. A. (2010). Multi-year participation in supplemental educational services: Effects and implications. *70*(12-A), 4570. | Unable to retrieve full text |
| Chaplin, D., & Puma, M. J. (2003). What "Extras" Do We Get with Extracurriculars? Technical Research Considerations. | Wrong study design |
| Christenson, S. L., Thurlow, M. L., Sinclair, M. F., Lehr, C. A., Kaibel, C. M., Reschly, A. L., . . . Pohl, A. (2008). Check & Connect: A Comprehensive Student Engagement Intervention Manual. | not a study |
| Cia, F., Barham, E. J., & Victorine Fontaine, A. M. G. (2010). Impacts of a parent intervention program: Their children's academic achievement and classroom behavior. *23*(3), 533-543. doi: <http://dx.doi.org/10.1590/S0102-79722010000300014>. | Wrong population |
| Clark, A. K., & Whetstone, P. (2014). The Impact of an Online Tutoring Program on Mathematics Achievement. *107*(6), 462-466. | Wrong setting |
| Clark, K., Jamison, T., & Sprague, D. (2005). Digital Study Groups: Online Learning Environments in Middle School. *3*(4). | Wrong study design |
| Clarke, J. S. (1993). Strategies Addressing Discrepancies in Educational and Behavioral Priorities and Expectations between Staff and Middle-Class K-5 Parents. | Wrong study design |
| Coenen, M. E. (2002). Using Gifted Students as Peer Tutors: An Effective and Beneficial Approach. *25*(1), 48-55. | Wrong study design |
| Coie, J. D., & Krehbiel, G. (1984). Effects of academic tutoring on the social status of low-achieving, socially rejected children. *55*(4), 1465-1478. doi: <http://dx.doi.org/10.2307/1130016>. | Wrong setting |
| Cole, C. A. (2009). Ten weeks of academic intervention designed to improve math word problem solving among middle school students: Effects of a randomized pilot study. *69*(7-A), 2600. | Unable to retrieve dissertation |
| Conrad, K. J., & Eash, M. J. (1983). Measuring implementation and multiple outcomes in a child parent center compensatory education program. *20*(2), 221-236. doi: <http://dx.doi.org/10.2307/1162595>. | Wrong setting |
| Conte, R., & Humphreys, R. (1989). Repeated readings using audiotaped material enhances oral reading in children with reading difficulties. *22*(1), 65-79. | Wrong setting |
| Cooledge, N. J., & Wurster, S. R. (1985). Intergenerational tutoring and student achievement. *39*(3), 343-346. | Wrong setting |
| Cox, D. D. (2005). Evidence-based interventions using home-school collaboration. *School Psychology Quarterly, 20*(4), 473-497. | Review/synthesis |
| Crosby, S. A., Rasinski, T., Padak, N., & Yildirim, K. (2015). A 3-Year Study of a School-Based Parental Involvement Program in Early Literacy. *108*(2), 165-172. | Wrong study design |
| Crozier, M., Rokutani, L., Russett, J. L., Godwin, E., & Banks, G. E. (2010). A Multisite Program Evaluation of Families and Schools Together (FAST): Continued Evidence of a Successful Multifamily Community-Based Prevention Program. *School Community Journal, 20*(1), 187-207. | Wrong study design |
| Daki, J., & Savage, R. S. (2010). Solution-Focused Brief Therapy: Impacts on Academic and Emotional Difficulties. *103*(5), 309-326. | Relevant, but lacks sufficient data |
| Daly, & Kupzyk, S. (2012). An Investigation of Student-Selected and Parent-Delivered Reading Interventions. *21*(4), 295-314. | Wrong study design |
| Davis, M., & Lyons, S. (2001). Improving Reading by...Reading: Ideas from Two Teachers. *8*(4), 51-57. | Wrong study design |
| Davis-Kennedy, P. (1996). The Effectiveness of Parental Involvement on Reading Achievement. | Wrong setting |
| Deaton, F. K. (1975). A comparison of the effects of reinforcing accuracy and on-task responses in a programmed remedial program with fourth-grade reading problem children. *35*(12-B, Pt 1), 6067-6068. | Unable to retrieve article |
| Deering, A. R. (1968). Homework Helper Program, Fact Sheet: New York City Board of Education, Brooklyn, NY. Office of State and Federally-Assisted Programs. | Wrong study design |
| Dietrich, C. (1972). Changes in Reading Achievement, Perceptual Motor Ability, and Behavior Adjustment as a Function of Perceptual Motor Training and Individualized Remedial Reading Instruction. Final Report: Wisconsin Univ., Stevens Point. Dept. of Psychology. | Wrong setting |
| Diken, I. H., Cavkaytar, A., Batu, S. E., Bozkurt, F., & Kurtyilmaz, Y. (2010). First Step to Success--A School/Home Intervention Program for Preventing Problem Behaviors in Young Children: Examining the Effectiveness and Social Validity in Turkey. *Emotional & Behavioural Difficulties, 15*(3), 207-221. | Wrong setting |
| Donah, S. (2013). The efficacy of an after-school based tutoring program in Orton-Gillingham approach. *73*(7-A(E)), No Pagination Specified. | Unable to retrieve full dissertation |
| dos Santos Elias, L. C., & Marturano, E. M. (2005). Language workshops: A psycho-educational intervention for children presenting academic complaints. *10*(1), 53-61. doi: <http://dx.doi.org/10.1590/S1413-294X2005000100007>. | Wrong study design |
| DuBois, M. R., Volpe, R. J., & Hemphill, E. M. (2014). A randomized trial of a computer-assisted tutoring program targeting letter-sound expression. *43*(2), 210-221. | Wrong setting |
| Eash, M. J., & et al. (1980). Assessment of Multiple Outcomes: An Evaluation Research Study of a Compensatory Early Childhood Program (Child Parent Centers and Child Parent Expansion Program). | Copy of report unreadable |
| Eisenberg, T., & et al. (1981). An Assessment of Cognitive Changes in Socially Disadvantaged Children as a Result of a One-to-One Tutoring Program. *74*(5), 311-314. | Wrong population |
| Ejlali, C. (1990). LIFE (Learning Is For Everyone) Program Evaluation, October 1, 1989-September 30, 1990): Washington County Board of Education, Jonesborough, TN. | Wrong study design |
| Elbaum, B., Vaughn, S., Tejero Hughes, M., & Watson Moody, S. (2000). How effective are one-to-one tutoring programs in reading for elementary students at risk for reading failure? A meta-analysis of the intervention research. *92*(4), 605-619. doi: <http://dx.doi.org/10.1037/0022-0663.92.4.605>. | Review/synthesis |
| Ellis, B. H., & et al. (1983). Implementation Assessment of a Parent Involvement Program and the Relationship between Level of Implementation and Child Achievement. | Unable to retrieve article |
| Ellis, M. G. (1996). *Parent-Child Reading Programs: Involving Parents in the Reading Intervention Process: Summary of Dissertation Research*. Retrieved from http://ovidsp.ovid.com/ovidweb.cgi?T=JS&CSC=Y&NEWS=N&PAGE=fulltext&D=eric3&AN=ED397377 | Duplicate |
| Evans, I. M., & et al. (1991). *Experimental Evaluation of a Preventative Home-School Partnership Program for At-Risk Elementary-Aged Children*. Retrieved from http://ovidsp.ovid.com/ovidweb.cgi?T=JS&CSC=Y&NEWS=N&PAGE=fulltext&D=eric2&AN=ED340493 | Relevant, but lacks sufficient data |
| Faires, J., Nichols, W. D., & Rickelman, R. J. (2000). Effects of Parental Involvement in Developing Competent Readers in First Grade. *21*(3), 195-215. | Relevant, but high risk of bias |
| Fantuzzo, J. W., & et al. (1995). Effects of Parent Involvement in Isolation or in Combination with Peer Tutoring on Student Self-Concept and Mathematics Achievement. *87*(2), 272-281. | Duplicate |
| Fashola, O. S. (1998). Review of Extended-Day and After-School Programs and Their Effectiveness: Center for Research on the Education of Students Placed At Risk, Baltimore, MD. | Review/synthesis |
| Feitler, F. C., & Hellekson, L. E. (1993). Active verbalization plus metacognitive awareness yields positive achievement gains in at-risk first graders. *33*(1), 1-11. doi: <http://dx.doi.org/10.1080/19388079309558139>. | Wrong setting |
| Fitzpatrick, J. W. (2013). Effects of a Technology-Based Parental-Involvement Program on Student Achievement in Mathematics. | Unable to retrieve dissertation |
| Fitzpatrick, J. W. (2014). Effects of a technology-based parental-involvement program on student achievement in mathematics. *74*(10-A(E)), No Pagination Specified. | Unable to retrieve article |
| Froiland, J. M. (2011). Parental Autonomy Support and Student Learning Goals: A Preliminary Examination of an Intrinsic Motivation Intervention. *40*(2), 135-149. | Wrong population |
| Fuchs, L. S., Powell, S. R., Seethaler, P. M., Cirino, P. T., Fletcher, J. M., Fuchs, D., . . . Zumeta, R. O. (2009). Remediating number combination and word problem deficits among students with mathematics difficulties: A randomized control trial. *101*(3), 561-576. doi: <http://dx.doi.org/10.1037/a0014701>. | Wrong setting |
| Fuerst, J. S., & Fuerst, D. (1993). Chicago Experience with an Early Childhood Programme: The Special Case of the Child Parent Center Program. *35*(3), 237-253. | Review/synthesis |
| Gabriel, K. (2013). The effect of intervention programs through the response to intervention framework on first grade reading achievement. *74*(4-A(E)), No Pagination Specified. | Unable to retrieve |
| Gao, J., Hallar, B., & Hartmann, T. A. (2014). A Snapshot of OST Programs in Philadelphia: An Evaluation of Eleven 21st Century Community Learning Center Grantees. | Inadequate study-info |
| Gerzel-Short, L. (2014). Response to intervention, family involvement, and student achievement at tier 2: A mixed methods study of K--1 students and their families. *74*(11-A(E)), No Pagination Specified. | Relevant, but not sufficient intervention information |
| Gilbert, J. K., Compton, D. L., Fuchs, D., Fuchs, L. S., Bouton, B., Barquero, L. A., & Cho, E. (2013). Efficacy of a First-Grade Responsiveness-to-Intervention Prevention Model for Struggling Readers. *48*(2), 135-154. | Wrong setting |
| Gillum, F. (2013). The impact of a counseling/tutorial program on at-risk students. *74*(2-A(E)), No Pagination Specified. | Wrong setting |
| Goodson, B. D., & Hess, R. D. (1976). The Effects of Parent Training Programs on Child Performance and Parent Behavior. | Review/synthesis |
| Goudey, J. (2010). A parent involvement intervention with elementary school students: The effectiveness of parent tutoring on reading achievement. *70*(11-A), 4225. | Wrong population |
| Green, S. K., Alderman, G., & Liechty, A. (2004). Peer Tutoring, Individualized Intervention, and Progress Monitoring with At-Risk Second- Grade Readers. *49*(1), 11-17. | Wrong setting |
| Haag, L. (2001). Is private tutoring effective?: An evaluation study. *15*(1), 38-44. doi: <http://dx.doi.org/10.1024//1010-0652.15.1.38>. | Language |
| Hailey, L. A. (2015). The effects of an after-school program: Changing academic performance and promoting success. *75*(8-A(E)), No Pagination Specified. | Unable to retrieve |
| Hara, S. R., & Burke, D. J. (1998). Parent Involvement: The Key to Improved Student Achievement. *8*(2), 9-19. | Inadequate study-info |
| Heikkila, R., Aro, M., Narhi, V., Westerholm, J., & Ahonen, T. (2013). Does training in syllable recognition improve reading speed? A computer-based trial with poor readers from second and third grade. *17*(6), 398-414. doi: <http://dx.doi.org/10.1080/10888438.2012.753452>. | Wrong setting |
| Heinrich, C. J., Burch, P., Good, A., Acosta, R., Cheng, H., Dillender, M., . . . Stewart, M. (2014). Improving the Implementation and Effectiveness of Out-of-School-Time Tutoring. *33*(2), 471-494. | Relevant, but insufficient data |
| Heinrich, C. J., Burch, P., Good, A., Acosta, R., Cheng, H. P., Dillender, M., . . . Stewart, M. (2014). IMPROVING THE IMPLEMENTATION AND EFFECTIVENESS OF OUT-OF-SCHOOL-TIME TUTORING. *33*(2), 471-+. doi:10.1002/pam.21745 | Duplicate |
| Heller, L. R., & Fantuzzo, J. W. (1993). RECIPROCAL PEER TUTORING AND PARENT PARTNERSHIP - DOES PARENT INVOLVEMENT MAKE A DIFFERENCE. *22*(3), 517-534. | Wrong setting |
| Herry, Y. (1987). Social behavior problems and reading remediation. *1*(2-3), 93-100. | Language |
| Hibpshman, T. L. (1989). An Explanatory Model for Family Literacy Programs. | Wrong study design |
| Hilger, L. H. (2001). Cross-age tutoring in reading: Academic and attitudinal effects from high-school tutors and third-grade tutees. *61*(10-A), 3941. | Wrong setting |
| Hindin, A., & Paratore, J. R. (2007). Supporting Young Children's Literacy Learning Through Home-School Partnerships: The Effectiveness of a Home Repeated-Reading Intervention. *39*(3), 307-333. | Wrong study design |
| Hoover, S. (1992). Coping with Multiple At-Risk Behaviors among Middle School Students through School and Systemic Interventions. | Wrong study design |
| Howe, N., Chambers, B., & Abrami, P. C. (1998). The effects of an academic restructuring program on parental attitudes and behaviors. *44*(1), 106-110. | Wrong outcomes |
| Invernizzi, M., & et al. (1996). A Community Volunteer Tutorial That Works. *50*(4), 304-311. | Wrong study design |
| Israel, B. L. (1968). Responsive Environment Program: Brooklyn, N.Y.; Report of the First Full Year of Operation. The Talking Typewriter: New York City Board of Education, Brooklyn, NY. Responsive Environment Program Center. | Unable to retrieve |
| Jacob, R. T., Smith, T. J., Willard, J. A., & Rifkin, R. E. (2014). Reading Partners: The Implementation and Effectiveness of a One-on-One Tutoring Program Delivered by Community Volunteers. Policy Brief: Mdrc. | Wrong setting |
| Jacobowitz, S. E. (1979). Tutoring by Parents of Their Children in First Grade. | Unable to retrieve article |
| Janowitz, G. (1968). *After-School Study Centers: Experimental Materials and Clinical Research. Final Report*. Retrieved from http://ovidsp.ovid.com/ovidweb.cgi?T=JS&CSC=Y&NEWS=N&PAGE=fulltext&D=eric1&AN=ED051342 | Relevant, but insufficient data |
| Jenkins-Stamper, R. L. (2009). The relationship between parental involvement and student achievement among students in special education programs in Dougherty County, State of Georgia. *70*(4-A), 1118. | Unable to retrieve article |
| Johnson, K. F., Gupta, A., Rosen, H., & Rosen, H. (2013). Improving Reading Comprehension through Holistic Intervening and Tutoring During After-School with High Risk Minority Elementary School Students. *21*(4), 431-443. | Wrong study design |
| Jones, L. (2013). Effective Strategies Used to Increase Early Reading Skills for Students in Third Grade. | Unable to retrieve |
| Jordan, E. W., & Tempest, P. (1998). Early Identification and Intervention of Navajo Students At Risk for Underachievement. | Wrong population |
| Kahle, A. L., & Kelley, M. L. (1994). Children's homework problems: A comparison of goal setting and parent training. *25*(2), 275-290. doi: <http://dx.doi.org/10.1016/S0005-7894%2805%2980288-6>. | Wrong outcomes |
| Kaplewicz, N. L. (2000). Effects of group play therapy on reading achievement and emotional symptoms among remedial readers. *61*(1-B), 535. | Wrong setting |
| Keller, G. (1988). The effectiveness of learning therapy for at-risk students. *35*(3), 230-233. | Unable to translate |
| Kirk, C., & Gillon, G. T. (2009). Integrated morphological awareness intervention as a tool for improving literacy. *40*(3), 341-351. doi: 10.1044/0161-1461(2008/08-0009). | Wrong population |
| Kolic-Vehovec, S. (2002). Self-monitoring and attribution training with poor readers. *44*(1), 57-68. | Wrong setting |
| Kratochwill, T. R., McDonald, L., Levin, J. R., Young Bear-Tibbetts, H., & Demaray, M. K. (2004). Families and Schools Together: An Experimental Analysis of a Parent-Mediated Multi-Family Group Program for American Indian Children. *42*(5), 359-383. | Wrong population |
| Kroeger, K. R. (1989). A Management System for Parental Reinforcement of Reading Skills for First Grade Chapter 1 Students. | Unable to retrieve information |
| Kupzyk, S. S. (2012). Teachers Engaging Parents as Tutors to Improve Oral Reading Fluency. Nebraska-Lincoln. | Wrong study design |
| Kyle, F., Kujala, J., Richardson, U., Lyytinen, H., & Goswami, U. (2013). Assessing the effectiveness of two theoretically motivated computer-assisted reading interventions in the United Kingdom: GG Rime and GG Phoneme. *48*(1), 61-76. | Wrong setting |
| Laffey, J. L., & et al. (1979). The Effect of Repeated Reading of Taped Literature on Reading Achievement. | Wrong study design |
| LaGoy, J. H. (1987). Improving Student Attendance and Achievement through Intervention of a Student Support/Home-School Liaison Committee. | Unable to retrieve information |
| Lai, F., Luo, R. F., Zhang, L. X., Huang, X. Z., & Rozelle, S. (2015). Does computer-assisted learning improve learning outcomes? Evidence from a randomized experiment in migrant schools in Beijing. *47*, 34-48. doi: 10.1016/j.econedurev.2015.03.005. | Wrong setting |
| Lane, H. B., Pullen, P. C., Hudson, R. F., & Konold, T. R. (2009). Identifying Essential Instructional Components of Literacy Tutoring for Struggling Beginning Readers. *48*(4), 277-297. | Wrong setting |
| Lauren, L., & Allen, L. (1999). Factors that predict success in an early literacy intervention project. *34*(4), 404-424. doi: <http://dx.doi.org/10.1598/RRQ.34.4.2>. | Wrong study design |
| Lavelle-Lore, M. D. (2014). Parent-Child Home Numeracy Intervention and the Mathematics Scores of First Grade Students in Urban Catholic Schools. | Unable to retrieve article |
| Leos-Urbel, J. (2015). What Works After School? The Relationship Between After-School Program Quality, Program Attendance, and Academic Outcomes. *47*(5), 684-706. doi: 10.1177/0044118x13513478. | Review/synthesis |
| MacKay, T., & Watson, K. (1999). Literacy, social disadvantage and early intervention: Enhancing reading achievement in primary school. *16*(1), 30-36. | Wrong setting |
| Madden, N. A., & et al. (1989). Success for All: First-Year Effects of a Comprehensive Plan for Reforming Urban Education: Center for Research on Elementary and Middle Schools, Baltimore, MD. | Wrong setting |
| Madden, N. A., Slavin, R. E., Wasik, B. A., & Dolan, L. J. (1997). Reading, writing, and language arts in Success for All. 109-130. | Wrong setting |
| Mantzicopoulos, P., Morrison, D., Stone, E., & Setrakian, W. (1992). Use of the SEARCH/TEACH tutoring approach with middle-class students at risk for reading failure. *92*(5), 573-586. doi: <http://dx.doi.org/10.1086/461707>. | Wrong setting |
| Markle, A., Rinn, R. C., & Goodwin, B. (1980). Effects of achievement motivation training on academic performance of underachievers. *47*(2), 567-574. doi: <http://dx.doi.org/10.2466/pr0.1980.47.2.567>. | Wrong setting |
| Martin, C. (2011). Providing Support to Families with Specific Regard to the Removal of Barriers that Exist for Families Trying to Provide Academic Support at Home. | Wrong population |
| Matefy, R. E. (1978). Evaluation of a remediation program using senior citizens as psychoeducational agents. *14*(4), 327-336. | Wrong population |
| McClain, D. B., Wolchik, S. A., Winslow, E., Tein, J. Y., Sandler, I. N., & Millsap, R. E. (2010). Developmental cascade effects of the New Beginnings Program on adolescent adaptation outcomes. *Development & Psychopathology, 22*(4), 771-784. doi: 10.1017/S0954579410000453. | Wrong intervention |
| McKinney, A. D. (1995). The effects of an after school tutorial and enrichment program on the academic achievement and self-concept of below grade level first and second-grade students. *56*(6-A), 2176. | Unable to retrieve full dissertation |
| McKinney, J. A. (1975). The Development and Implementation of a Tutorial Program for Parents to Improve the Reading and Mathematics Achievement of Their Children. | Wrong population |
| Mears, P. R. (2008). The effects of the fast start program on the reading achievement of emergent and beginning readers: A replication and extension. *68*(11-A), 4650. | Wrong population |
| Meier, J. D., & Invernizzi, M. (1999). Book Buddies in the Bronx: A Model for America Reads: Center for the Improvement of Early Reading Achievement, Ann Arbor, MI. | Wrong setting |
| Meier, J. D., & Invernizzi, M. (2001). Book Buddies in the Bronx: Testing a Model for America Reads. *6*(4), 319-333. | Wrong setting |
| Menesses, K. F., & Gresham, F. M. (2009). Relative Efficacy of Reciprocal and Nonreciprocal Peer Tutoring for Students At-Risk for Academic Failure. *24*(4), 266-275. | Wrong setting |
| Merriman, D. E. (2011). The effects of group coaching on the homework completion of secondary students with homework problems. *72*(5-A), 1541. | Unable to retrieve full dissertation |
| Meteyer, B. V. (1998). The effects of a manual-based parent training program on parent perceptions of homework problems, on mathematics homework, and on mathematics achievement. *59*(3-A), 0725. | Unable to retrieve full dissertation |
| Miller, A. L., & Narrett, C. M. (1995). Does Parent Involvement and Parent Feedback about Reading Progress Influence Students' Reading Progress? | Wrong population |
| Miller, B. V. (1995). An evaluation of the paired reading program using competency-based training. *55*(7-A), 1888. | Unable to retrieve article |
| Miller, S., & Connolly, P. (2013). A Randomized Controlled Trial Evaluation of Time to Read, a Volunteer Tutoring Program for 8-to 9-Year-Olds. *35*(1), 23-37. doi: 10.3102/0162373712452628. | Wrong setting |
| Mischo, C., & Haag, L. (2002). Expansion and Effectiveness of Private Tutoring. *17*(3), 263-273. | Wrong intervention |
| Moon, T. R., & Callahan, C. M. (2001). Curricular Modifications, Family Outreach, and a Mentoring Program: Impacts on Achievement and Gifted Identification in High-Risk Primary Students. *24*(4), 305-321. | Wrong setting. |
| Morris, D., Tyner, B., & Perney, J. (2000). Early Steps: Replicating the effects of a first-grade reading intervention program. *92*(4), 681-693. doi: <http://dx.doi.org/10.1037/0022-0663.92.4.681>. | Wrong setting |
| Morrison, T. A. (2010). The impact of a family home-learning program on levels of parental/caregiver efficacy. *71*(2-A), 413. | Wrong population |
| Munoz, M. A. (2002). Outcome-Based Community-Schools Partnerships: The Impact of the After-School Programs on Non-Academic and Academic Indicators. | Wrong outcomes. |
| Munoz, M. A., Chang, F., & Ross, S. M. (2012). No Child Left Behind and Tutoring in Reading and Mathematics: Impact of Supplemental Educational Services on Large Scale Assessment. *17*(3), 186-200. | Duplicate |
| Murad, C. R., & Topping, K. J. (2000). Parents as Reading Tutors for First Graders in Brazil. *21*(2), 152-171. | Wrong population |
| Musher, K. K., Musher, D. M., Graviss, & Strudler, R. M. (2005). Can an Intense Educational Experience Improve Performance on Objective Tests? Results from One Charter School. *69*(4), 352-366. | Wrong intervention |
| Naylor, J., & Pumfrey, P. D. (1983). The alleviation of psycholinguistic deficits and some effects on the reading attainments of poor readers: A sequel. *6*(2), 129-153. doi: <http://dx.doi.org/10.1111/j.1467-9817.1983.tb00247.x> | Wrong setting |
| Nazzal, A. (2002). Peer Tutoring and At-Risk Students: An Exploratory Study. *24*(1), 68-80. | Wrong population |
| Nutter, N., & Safran, S. P. (1983). Sentence Combining and the Learning Disabled Student. | Wrong setting |
| O'Donnell, C. R., Chambers, E., & Ling, K. (1973). Athletics as reinforcement in a community program for academic achievement. *1*(2), 212-214. doi: <http://dx.doi.org/10.1002/1520-6629%28197304%291:2%3C212::AID-JCOP2290010217%3E3.0.CO;2-J>. | Wrong study design. |
| Olympia, D. E., Sheridan, S. M., Jenson, W. R., & Andrews, D. (1994). USING STUDENT-MANAGED INTERVENTIONS TO INCREASE HOMEWORK COMPLETION AND ACCURACY. *27*(1), 85-99. doi: 10.1901/jaba.1994.27-85. | Wrong population |
| Otto, B., & Muller, C. (2015). Effects of Institutional Tutoring on Self-Regulated Learning and Math Performance. *62*(4), 285-302. doi: 10.2378/peu2015.art21d. | Language |
| Outland, B. A. (1975). The Effects of a Teacher-Involved Parent Education Program on Verbal Achievement of Disadvantaged Black Pupils in the First Grade in Selected Macon County, Alabama Schools. | Unable to retrieve dissertation |
| Paramore, B., & et al. (1973). Project Upswing After Two Years: An Evaluation: Operations Research, Inc., Silver Spring, MD. | Wrong setting |
| Philliber, W. W., & et al. (1996). Consequences of Family Literacy for Adults and Children: Some Preliminary Findings. *39*(7), 558-565. | Wrong population. |
| Plantec, P., & et al. (1972). Final Report on the Evaluation of Project Upswing's First Year: Operations Research, Inc., Silver Spring, MD. | Wrong setting. |
| Portwood, S. G., Brooks-Nelson, E., & Schoeneberger, J. (2015). Data and Evaluation Strategies to Support Parent Engagement Programs: Learnings from an Evaluation of Parent University. *37*(3), 145-153. | Wrong population. |
| Powell, S. R., & Driver, M. K. (2015). The Influence of Mathematics Vocabulary Instruction Embedded Within Addition Tutoring for First-Grade Students With Mathematics Difficulty. *38*(4), 221-233. doi: 10.1177/0731948714564574. | Wrong setting. |
| Quigley, D. D. (2000). Parents and Teachers Working Together To Support Third Grade Achievement: Parents as Learning Partners (PLP) Findings. | Wrong population. |
| Raim, J. (1980). Who Learns when Parents Teach Children? *, 34*(2), 152-155. | Wrong study design |
| Reglin, G. L. (1988). Effects of a Computer Assisted Remediation Program on Basic Skills Mathematics Achievement, Academic Self-Concept, and Locus of Control of Students in a Selected Dropout Retrieval Program in an Urban Setting. | Wrong setting |
| Reynolds, A. J. (2000). Success in Early Intervention: The Chicago Child-Parent Centers. | Unable to retrieve book |
| Reynolds, D., & Nicolson, R. I. (2007). Follow-up of an exercise-based treatment for children with reading difficulties. *Dyslexia, 13*(2), 78-96. doi: <http://dx.doi.org/10.1002/dys.331>. | Wrong population |
| Riconscente, M. M. (2013). Results from a controlled study of the iPad fractions game Motion Math. *8*(4), 186-214. | Wrong setting |
| Rimm-Kaufman, S. E., Kagan, J., & Byers, H. (1999). The Effectiveness of Adult Volunteer Tutoring on Reading among "At Risk" First Grade Children. *38*(2), 143-152. | Wrong setting |
| Ritchey, K. D., Silverman, R. D., Montanaro, E. A., Speece, D. L., & Schatschneider, C. (2012). Effects of a Tier 2 Supplemental Reading Intervention for At-Risk Fourth-Grade Students. *78*(3), 318-334. | Wrong setting |
| Roberts, G. J. (1985). Springfield Public Schools Chapter 636 Programs 1984-85: Springfield Public Schools, MA. | Unable to retrieve report |
| Robledo, M. d. R. (1986). Texas School Dropout Survey Project: A Summary of Findings: Intercultural Development Research Association, San Antonio, TX. | Unable to retrieve report |
| Root, R. W., & Levant, R. F. (1984). An evaluation of parent effectiveness training for rural parents. *5*(2), 45-54. | Wrong population |
| Ross, S. M., Smith, L. J., & Casey, J. P. (1999). "Bridging the gap": The effects of the success for all program on elementary school reading achievement as a function of student ethnicity and ability level. *10*(2), 129-150. doi: 10.1076/sesi.10.2.129.3504. | Wrong setting |
| Russell, C. A., Mielke, M. B., & Reisner, E. R. (2009). Evidence of Program Quality and Youth Outcomes in the DYCD Out-of-School Time Initiative: Report on the Initiative's First Three Years. Executive Summary. | Relevant, but no outcome data |
| Rymanowski, J. L. (2012). School-home performance feedback with home-based writing activities: The effects on elementary students' writing fluency. *73*(1-A), 74. | Wrong population |
| Sanford, E. E. (2010). Examining the Impact of Complementary Assistance Learning on Student CRCT Scores. | Unable to retrieve full dissertation |
| Sapp, M. (1996). Irrational beliefs that can lead to academic failure for African American middle school students who are academically at-risk. *14*(2), 123-134. doi: <http://dx.doi.org/10.1007/BF02238186>. | Wrong study design |
| Scheiner, L. (1969). An Evaluation of the Sulzberger Reading Laboratory Clinic, 1968-1969: Philadelphia School District, PA. Office of Research and Evaluation. | Wrong setting |
| Schnelker, D., & Tompkins, J. (1988). The Des Moines Plan: A Plan for Student Success, 1987-88. Report of Evaluation: Des Moines Public Schools, IA. Dept. of Evaluation, Research, and Testing. | Wrong setting |
| Schroeder, R. H. (1982). Improvement in academic achievement through enhancement of perceptual and sensory integrative functioning. *3*(2), 97-103. | Wrong setting |
| Searls, E. F., Lewis, M. B., & Morrow, Y. B. (2006). Parents as Tutors‐It Works! *Reading Psychology, 3*(2), 117-129. doi: 10.1080/0270271820030204. | Wrong study design |
| Sebastian, J. (2014). The impact of an after-school intervention program on academic achievement among middle school students. *74*(11-A(E)), No Pagination Specified. | Unable to retrieve full dissertation |
| Shah, K. G. (2007). Further study of the treatment utility of the Woodcock Johnson III using matched and evidence-based interventions. *68*(4-A), 1330. | Wrong design |
| Shaver, J. P. (1969). Tutorial Students Two Years Later: A Report on the Logan-Cache Tutorial Center for Underachieving Readers and Writers: Utah State Dept. of Public Instruction, Salt Lake City. | Inadequate study-info |
| Shuttleworth, D. (1986). Parents-as-Partners. *26*(2), 41-43. | Inadequate study-info |
| Siders, M. B., & Sledjeski, S. (1978). How to Grow a Happy Reader: Report on a Study of Parental Involvement As It Relates to a Child's Attitudes and Achievement in the Acquisition of Reading Skills. Research Monograph No. 27: Florida Univ., Gainesville. P. K. Yonge Lab. School. | Wrong population |
| Silverstein, L. (1976). Project Turnabout, School Year 1975-1976: New York City Board of Education, Brooklyn, NY. Office of Educational Evaluation. | Wrong study design |
| Singh, B. R. (1997). Using Urban Resources for Raising Educational Standards in Urban Areas. *20*, 23-37. | Review/synthesis |
| Skuy, M., & Solomon, W. (1980). Effectiveness of students and parents in a psycho-educational intervention programme for children with learning problems. *8*(1), 76-86. doi: <http://dx.doi.org/10.1080/03069888000760081>. | Wrong population |
| Slade, K. (1988). Parents as Active Members in the School's Reading Program. | not a study |
| Slater, J., Strait, D. L., Skoe, E., O'Connell, S., Thompson, E., & Kraus, N. (2014). Longitudinal Effects of Group Music Instruction on Literacy Skills in Low-Income Children. *9*(11), 9. doi: 10.1371/journal.pone.0113383. | Wrong setting |
| Slavin, R. E., & Madden, N. A. (1999). Roots & Wings: Effects of Whole-School Reform on Student Achievement: Center for Research on the Education of Students Placed At Risk, Baltimore, MD. | Wrong study design |
| Spaulding, R. L. (1972). Effects of a Five-Year Compensatory Education Program on Social, Intellectual, Linguistic, and Academic Development: Duke Univ., Durham, NC. | Wrong setting |
| Strang, H. R. (1971). An Automated Audio-Visual Approach to Remediate Reading Problems. Final Report: Virginia Univ., Charlottesville. | Wrong setting |
| Studer-Luethi, B., Bauer, C., & Perrig, W. J. (2015). Working memory training in children: Effectiveness depends on temperament. doi: 10.3758/s13421-015-0548-9. | Wrong setting |
| Taylor, J., & Cox, B. D. (1997). Microgenic analysis of group-based solution of complex two-step mathematical word problems by fourth graders. *6*(2), 183-226. doi: <http://dx.doi.org/10.1207/s15327809jls0602_2>. | Wrong setting |
| Taylor, J. L. (1973). Remedial education of children in foster care. *52*(2), 123-128. | Wrong study design |
| Theodore, L. A., DioGuardi, R. J., Hughes, T. L., Aloiso, D., Carlo, M., & Eccles, D. (2009). A Class-Wide Intervention for Improving Homework Performance. *Journal of Educational & Psychological Consultation, 19*(4), 275-299. doi: 10.1080/10474410902888657. | Wrong intervention |
| Thurston, L. P., & Dasta, K. (1990). An Analysis of In-Home Parent Tutoring Procedures: Effects on Children's Academic Behavior at Home and in School and on Parents' Tutoring Behaviors. *11*(4), 41-52. | Wrong population |
| Tizard, J., & et al. (1982). Collaboration between Teachers and Parents in Assisting Children's Reading. *52*, 1-15. | Wrong population |
| Tomesen, M., & Aarnoutse, C. (1997). Effects of a Training Program in Deriving Word Meanings. | Wrong setting |
| Topping, K. J. (1987). Peer tutored paired reading: Outcome data from ten projects. *7*(2), 133-145. doi: <http://dx.doi.org/10.1080/0144341870070206>. | Review/synthesis |
| Topping, K. J., & Lindsay, G. A. (1991). Parental involvement in reading: The influence of socio-economic status and supportive home visiting. *5*(4), 306-316. doi:http://dx.doi.org/10.1111/j.1099-0860.1991.tb00496.x | Inaccessible full text |
| Tsuei, M. (1171). Using Synchronous Peer Tutoring System to Promote Elementary Students' Learning in Mathematics. *58*(4), 1171-1182. | Wrong setting |
| Tyler, E. J., Hughes, J. C., Beverley, M., & Hastings, R. P. (2015). Improving early reading skills for beginning readers using an online programme as supplementary instruction. *30*(3), 281-294. doi: <http://dx.doi.org/10.1007/s10212-014-0240-7>. | Wrong setting |
| Vadasy, P. E., Sanders, E. A., & Tudor, S. (2007). Effectiveness of paraeducator-supplemented individual instruction: Beyond basic decoding skills. *40*(6), 508-525. doi: 10.1177/00222194070400060301. | Wrong setting |
| Vadasy, P. F., Jenkins, J. R., & Pool, K. (2000). Effects of tutoring in phonological and early reading skills on students at risk for reading disabilities. *33*(6), 579-590. | Wrong setting |
| Vadasy, P. F., Jenkins, J. R., Antil, L. R., & Wayne, S. K. (1997). Community-based early reading intervention for at-risk first graders. *12*(1), 29-39. | Relevant, but high risk of bias |
| Vadasy, P. F., Jenkins, J. R., Antil, L. R., Wayne, S. K., & O'Connor, R. E. (1997). The effectiveness of one-to-one tutoring by community tutors for at-risk beginning readers. *20*(2), 126-139. doi:http://dx.doi.org/10.2307/1511219 | Dual publication |
| Vaden-Kiernan, M., Jones, D. H., Rudo, Z., Fitzgerald, R., Hartry, A., Chambers, B., . . . Moss, M. A. (2008). The National Partnership for Quality Afterschool Learning Randomized Controlled Trial Studies of Promising Afterschool Programs: Summary of Findings. Afterschool Research Brief. Issue No. 3. | Review/synthesis |
| van den Heuvel-Panhuizen, M., Kolovou, A., & Robitzsch, A. (2013). Primary School Students' Strategies in Early Algebra Problem Solving Supported by an Online Game. *84*(3), 281-307. | Wrong population |
| van der Werf, G., Creemers, B., & Guldemond, H. (2001). Improving parental involvement in primary education in Indonesia: Implementation, effects and costs. *12*(4), 447-466. | Wrong population |
| Van Voorhis, F. L. (2011). Adding Families to the Homework Equation: A Longitudinal Study of Mathematics Achievement. *43*(3), 313-338. | Wrong population |
| Van Voorhis, F. L. (2011). Costs and Benefits of Family Involvement in Homework. *22*(2), 220-249. | Wrong population |
| Venzen, M. A. (2011). Impact of the Twenty-First Century Afterschool Program on Student Achievement in Mathematics and Language Arts. | Unable to retrieve dissertation |
| Villiger, C., Niggli, A., Wandeler, C., & Kutzelmann, S. (2012). Does family make a difference? Mid-term effects of a school/home-based intervention program to enhance reading motivation. *Learning and Instruction, 22*(2), 79-91. doi: 10.1016/j.learninstruc.2011.07.001. | Wrong population |
| Voges, W. (1977). Changes of educational attitudes following parent training. *24*(5), 310-313. | Wrong outcomes |
| Wade, J., & Kass, C. E. (1987). Component deficit and academic remediation of learning disabilities. *20*(7), 441-447. doi: <http://dx.doi.org/10.1177/002221948702000714>. | Wrong intervention |
| Wandeler, C., Niggli, A., Trautwein, U., & Hugo, C. V. (2013). Homework assistance as accompanying measure to school-based reading promotion: Preventive effects of a parental training. *60*(4), 253-266. | Language |
| Watson, T., & Hempenstall, K. (2008). Effects of a Computer Based Beginning Reading Program on Young Children. *24*(3), 258-274. | Wrong population |
| Weinstein, J. (2012). Program evaluation of an after-school tutoring service. *72*(9-B), 5584. | Unable to retrieve |
| Weitzman, D. L. (1964). Effect of Tutoring on Performance and Motivation Ratings in Secondary School Students: Oakland Unified School District, CA. | Unable to retrieve |
| Welsh, D. J., & et al. (1981). Title I Parents as Compensatory Reading Instructors: Is There No Place Like Home? Publication No. 80.58. | Unable to retrieve |
| Wheldall, K. (2000). Does Rainbow Repeated Reading add value to an intensive literacy intervention program for low-progress readers? An experimental evaluation. *52*(1), 29-36. doi: <http://dx.doi.org/10.1080/00131910097388>. | Wrong population |
| Wijekumar, K. K., Meyer, B. J. F., & Lei, P. (1013). Large-Scale Randomized Controlled Trial with 4th Graders Using Intelligent Tutoring of the Structure Strategy to Improve Nonfiction Reading Comprehension. *60*(6), 987-1013. | Wrong setting |
| Williams, M. G. L. (2011). Parental intervention: Effects on reading comprehension skills in Black children in kindergarten through fourth grade. *72*(4-A), 1228. | Unable to retrieve full dissertation |
| Wittenberg, D. (2009). Effectiveness of Individual versus Group Tutoring Programs on Reading Skills for Children in First through Third Grade Who Are At-Risk for Reading Difficulties. | Wrong setting |
| Yancsurak, L. S. (2014). Effectiveness of a computer-based afterschool intervention to increase reading comprehension. *74*(8-A(E)), No Pagination Specified. Mangler pdf | Wrong population |
| Yap, K. O. (1987). Improving Chapter 1 through Parents: A Family Goal Program. | Wrong population |
| Zimmer, R., Hamilton, L., & Christina, R. (2010). After-school tutoring in the context of no Child Left Behind: Effectiveness of two programs in the Pittsburgh Public Schools. *Economics of Education Review, 29*(1), 18-28. doi: 10.1016/j.econedurev.2009.02.005 | Wrong study design |
| Zimmermann, M. J., & Sassenrath, J. M. (1978). Improvement in Arithmetic and Reading and Discovery Learning in Mathematics (SEED). *3*(1), 27-33. | Wrong setting |
| Zinn, A., & Courtney, M. E. (2014). Context matters: Experimental evaluation of home-based tutoring for youth in foster care. *Children and Youth Services Review, 47*(Part 3), 198-204. doi: 10.1016/j.childyouth.2014.08.017 | Wrong population |

## Updated search from 2016 to November 2019

Upon publication of the review, the initial search was somewhat dated. Fully updating the review and analyses of common elements was not feasible due to resource constraints. However, a pragmatic update was completed to provide an estimation of new eligible studies and whether they would be likely to influence results. The first author completed a review and selection process, including risk of bias assessments of articles included for full text reviews. Details are provided below.

**Search and selection**

Searches in the databases accounting for the vast majority of records found eligible in the original search was re-run for records published between 2016 and November 4^th^ 2019. We searched PsycINFO, ERIC, Cochrane Library, and MEDLINE and identified 2091 records.

PsycINFO: 769 records

ERIC: 556 records

Cochrane Library: 369 records

MEDLINE: 397 records

Thirty-three full text articles were retrieved after screening abstracts. The following four studies were found eligible after full text screening and risk of bias assessment:

Harper, J., & Schmidt, F. (2016). Effectiveness of a group-based academic tutoring program for children in foster care: A randomized controlled trial. *Children and Youth Services Review*, *67*, 238-246.

Huang, X., Craig, S. D., Xie, J., Graesser, A., & Hu, X. (2016). Intelligent tutoring systems work as a math gap reducer in 6th grade after-school program. *Learning and Individual Differences*, *47*, 258-265.

Baker, S. K., Kamata, A., Wright, A., Farmer, D., & Nippert, R. (2019). Using propensity score matching to estimate treatment effects of afterschool programs on third‐grade reading outcomes. *Journal of community psychology*, *47*(1), 117-134.

Bayless, S. D., Jenson, J. M., Richmond, M. K., Pampel, F. C., Cook, M., & Calhoun, M. (2018, August). Effects of an Afterschool Early Literacy Intervention on the Reading Skills of Children in Public Housing Communities. In *Child & Youth Care Forum* (Vol. 47, No. 4, pp. 537-561). Springer US.

**Harper & Schmidt (2016)**

This article reports on the final results from a study already included in the review (Harper & Schmidt, 2012) which evaluates small group tutoring following the Teach Your Children Well program. Similar to the preliminary results reported in 2012, the 2016 article reports significant effects on reading, with effect sizes ranging from small to medium (reading *g = .40,* spelling *g = .25,* sentence comprehension *g = .15).* In their 2012 preliminary evaluation, the intervention did not have significant effects on math. In the final 2016 evaluation, however, there were significant effects on math achievement as well, with medium effect size (*g= .34).* Thus, including this article would not have influenced common elements results for reading, however, the practice elements coded negative for math (structured tutoring and positive reinforcement), and subsequent process- and elements combined with these practice elements, would have been changed to positive coding.

**Huang et al. (2018)**

This study was a quasi-experimental factorial trial with six factors. One factor was a web-based tutoring system in math, another was teacher led math instruction and the remaining were different demographic variables. The tutoring system, ALEKS, used artificial intelligence based on Knowledge Space Theory to provide 6^th^ grade students with skill level-appropriate mathematical problems which continuously adapted as students progressed. ALEKS also provided instant feedback about skill level and what a student was ready to learn next. The results did not fulfill the effectiveness criteria of this review due to a lack of significant differences between conditions. Low-SES students experienced more benefits from ALEKS compared to the teacher led math instruction, however, the result was not statistically significant. This study would have had some minor influence on results of common elements in the review, mainly in reducing the frequency count value of structured tutoring in math, and in the process- and implementation elements used in combination with it. However, structured tutoring would still be a highly common practice element. The following elements would have been coded as minus:

- *Practice elements*: structured tutoring in math, digital math computation,
- *Process elements:* web-based delivery, individualized, flexible/adaptive, progressive difficulty, feedback on performance, repeated training, intensive dosage (4h a week) over longer period of time (up to six months), use of artificial intelligence (new process element)
- *Other characteristics:* Use of Knowledge Space Theory (new characteristic), 6^th^ graders indicated risk (low SES),
- *Implementation elements:* educational meeting, educational material, local technical assistance

**Bayless et al. (2018)**

This is a quasi-experimental evaluation of a community based after school program (ASP) for children and youth residing in public housing communities. The ASP included a small group reading intervention (Read Well), one-on-one tutoring by trained volunteers, and a home literacy program (GR8 Readers) providing children with a home reading library. The study had substantial attrition, and even though several appropriate steps were taken to address this, the extensiveness of the attrition raises questions about the validity of the results. As discussed in the manuscript, certain biases or intensity of bias should perhaps be weighted differently in Risk of Bias assessments in future reviews, and this study could be an example of such due its substantial attrition.

Compared to a passive control sample and to a match comparison sample, the ASP sample scored significant better on reading skills with small effect sizes. This study is one of few that reports some detailed fidelity data, and the Read Well intervention was the most delivered intervention. When more studies report fidelity-details (and preferably on the level of elements), we will be able to weight coding based on fidelity in our future common elements-reviews. The following elements would have received a plus coding in this review and would have had a minor influence on common elements results:

- *Practice elements*: structured tutoring in reading, literacy training, reading alone, give books
- *Process elements for structured tutoring and literacy training:* Delivered by professional, delivered by trained volunteer, group delivery, one-on-one delivery, repeated training, flexible/adaptive, monitoring performance, feedback on performance, limited dosage over longer period of time,
- *Process elements for giving books and reading alone:* educational material, individualized, user-involvement children, culturally sensitive
- *Other characteristics:* K to 3. graders indicated risk (low SES and public housing),
- *Implementation elements:* Quality monitoring, educational meetings, local technical assistance, provide incentives (give books to keep after 4-5 tutoring sessions), purposefully reexamine implementation, audit and feedback

**Baker et al. 2018**

This study evaluated community-based afterschool programs (ASP) for at-risk students’ third grade reading achievement. Seven ASPs provided unspecified reading support to directly or indirectly improve reading achievement. The support varied from intense one-on-one tutoring interventions to programs focusing more on homework support and reading practices. ASPs were found effective compared to a passive comparison sample with medium effect sizes. No further details about interventions are provided, limiting coding of elements. The practice elements unspecified tutoring, unspecified literacy training, and unspecified homework support would have received a plus coding. This study would not have affected results concerning common elements.

| Excluded after full text review in updated search | Reasons |
| --- | --- |
| Jenson, J. M., Veeh, C., Anyon, Y., Mary, J. S., Calhoun, M., Tejada, J., & Lechuga-Peña, S. (2018). Effects of an afterschool program on the academic outcomes of children and youth residing in public housing neighborhoods: A quasi-experimental study. *Children and Youth Services Review*, *88*, 211-217. | Same study as Bayless et al. 2018 |
| Peña, S. L. (2016). Improving Children's Academic Performance through Parent Engagement: Development and Initial Findings from the Your Family, Your Neighborhood Intervention. | High risk of bias |
| Acosta, M. M., & Duggins, S. (2018). Community-Based Literacy Learning Spaces as Counterhegemonic Figured Worlds for African American Readers. *Reading Horizons: A Journal of Literacy and Language Arts*, *57*(3), 4. | No statistical analyses reported |
| Anderson, P. J., Lee, K. J., Roberts, G., Spencer-Smith, M. M., Thompson, D. K., Seal, M. L., ... & Doyle, L. W. (2018). Long-term academic functioning following Cogmed working memory training for children born extremely preterm: a randomized controlled trial. *The Journal of pediatrics*, *202*, 92-97. | Wrong population |
| Arrimada, M., Torrance, M., & Fidalgo, R. (2018). Supporting first-grade writers who fail to learn: multiple single-case evaluation of a Response to Intervention approach. *Reading and Writing*, *31*(4), 865-891. | Wrong setting |
| Aunio, P., & Mononen, R. (2018). The effects of educational computer game on low-performing children’s early numeracy skills–an intervention study in a preschool setting. *European Journal of Special Needs Education*, *33*(5), 677-691. | Wrong population |
| Barton, J. M. (2016). Mathematics Intervention Utilizing Carnegie Learning's Cognitive Tutor® and Compass Learning's Odyssey Math®, ProQuest LLC. | Wrong setting |
| Bench, B. D. (2018). The effects of academic parent teacher teams on Latino student achievement. Walden Dissertations and Doctoral Studies. | High risk of bias |
| Bellon, E. O., et al. (2017). "The Role of Parental Leadership in Academic Performance: A Case of Pupils in the Free Primary Education Program in Kenya." Education and Urban Society **49**(1): 110-130. | Wrong intervention |
| Bryant, B. R., et al. (2016). "The Effects of a Tier 3 Intervention on the Mathematics Performance of Second Grade Students with Severe Mathematics Difficulties." Journal of Learning Disabilities **49**(2): 176-188. | Wrong setting |
| D'Costa, S. (2018). "Examining the differential effects of an out-of-school time intervention on English language learners with varying levels of opportunity-to-learn." Dissertation Abstracts International Section A: Humanities and Social Sciences **79**(1-A(E)): No Pagination Specified. | Wrong design |
| Fives, A. (2016). "Modeling the Interaction of Academic Self-Beliefs, Frequency of Reading at Home, Emotional Support, and Reading Achievement: An RCT Study of At-Risk Early Readers in First Grade and Second Grade." Reading Psychology **37**(3): 339-370. | Wrong setting and intervention |
| Goncalves, L. L., et al. (2017). "Massage and Storytelling Reduce Aggression and Improve Academic Performance in Children Attending Elementary School." Occupational Therapy International **2017**: 5087145. | Wrong setting |
| Hassler Hallstedt, M., et al. (2018). "Short and long-term effects of a mathematics tablet intervention for low performing second graders." Journal of Educational Psychology **110**(8): 1127-1148. | Wrong setting |
| Hathaway, D. A. (2019). "Afterschool program effectiveness on literacy growth for children attending a high poverty school." Dissertation Abstracts International Section A: Humanities and Social Sciences **80**(4-A(E)): No Pagination Specified. | Wrong population |
| Hotulainen, R., et al. (2016). "Thinking Skills Intervention for Low-Achieving First Graders." European Journal of Special Needs Education **31**(3): 360-375. | Wrong setting |
| Houri, A. K., et al. (2019). "Targeting Parent Trust to Enhance Engagement in a School-Home Communication System: A Double-Blind Experiment of a Parental Wise Feedback Intervention." School Psychology **34**(4): 421-432. | Wrong outcomes |
| Kershner, E. E. S. (2019). "A program evaluation of an after-school reading intervention program in a small urban elementary school." Dissertation Abstracts International Section A: Humanities and Social Sciences **80**(6-A(E)) | Wrong design |
| Kupzyk, S. S. and E. J. Daly, III (2017). "Teachers Engaging Parents as Reading Tutors." Contemporary School Psychology **21**(2): 140-151. | Wrong design |
| Lahti, M., et al. (2019). "Parents as Teachers (PAT) home-visiting intervention: A path to improved academic outcomes, school behavior, and parenting skills." Children and Youth Services Review **99**: 451-460. | Wrong population |
| Mononen, R. and P. Aunio (2016). "Counting Skills Intervention for Low-Performing First Graders." South African Journal of Childhood Education **6**(1). | Wrong setting |
| Oseji, C. U. (2016). "The effectiveness of reading recovery in a Metro Atlanta, Georgia Elementary School." Dissertation Abstracts International Section A: Humanities and Social Sciences **76**(11-A(E)): No Pagination Specified. | Wrong setting |
| Proscovia, N., et al. (2019). "Assessing the impact of an asset-based intervention on educational outcomes of orphaned children and adolescents: findings from a randomised experiment in Uganda." Asia Pacific Journal of Social Work **29**(1): 59-69. | Wrong intervention |
| Roschelle, J., et al. (2016). "Online Mathematics Homework Increases Student Achievement." AERA Open **2**(4). | Wrong intervention |
| Schaeffer, M. W., et al. (2018). "Disassociating the relation between parents' math anxiety and children's math achievement: Long-term effects of a math app intervention." Journal of Experimental Psychology: General **147**(12): 1782-1790. | Wrong population |
| Solís, M., et al. (2017). "Text-Based Vocabulary Intervention Training Study: Supporting Fourth Graders with Low Reading Comprehension and Learning Disabilities." Learning Disabilities: A Contemporary Journal **15**(1): 103-115. | Wrong setting |
| Spencer, R., et al. (2018). Impact Evaluation of Partners Advancing Childhood Education ("PACE"), Grantee Submission. | Not a study |
| Taylor, L. (2018). "The effects of leveled literacy intervention for students in the RtI process." Dissertation Abstracts International Section A: Humanities and Social Sciences **79**(1-A(E)): No Pagination Specified. | Wrong setting and intervention |
| Watson-Huggins, J. (2018). An Experimental Study on the Effects of a Gamified Software Intervention in Mathematics Achievement among Sixth Grade Students, ProQuest LLC. | Wrong setting |
