# Supplementary material for: Common Elements of Practice, Process and Implementation in Out-of-School-Time Academic Interventions for At-risk Children: a Systematic Review
Source: Prev Sci. 2020 Feb 4;21(4):545–56. doi: 10.1007/s11121-020-01091-w (PMC7162823; doi:10.1007/s11121-020-01091-w)
Supplement: Supplementary file 2 — (DOCX 58.1 kb) [file 11121_2020_1091_MOESM2_ESM.docx]

Identifying Common Elements of interventions

Manual for coding, extraction and analyses

Thomas Engell and Hege Kornør

*The Regional Centre for Child and Adolescent Mental Health, Eastern and Southern Norway*

*© Thomas Engell, 2019*

*Contact:* [*Thomas.engell@r-bup.no*](mailto:Thomas.engell@r-bup.no)

Supplementary file 2 of the publication:

Common Elements of Practice, Process and Implementation in Out-of-School-Time Academic Interventions for At-risk Children: a Systematic Review

Engell, T., Kirkøen, B., Hammerstrøm, K.T., Kornør, H., Ludvigsen, K.H., Hagen, K.A

*Prevention Science*

Content

[Introduction 2](#_Toc17193803)

[What are Common Elements? 3](#_Toc17193804)

[Review process 3](#_Toc17193805)

[Selecting studies for coding 3](#_Toc17193806)

[Criteria for study quality and effectiveness used in the common elements review of effective OSTA interventions 4](#_Toc17193807)

[Limitations due to effectiveness criteria 4](#_Toc17193808)

[Coding of ineffective interventions in the common elements review of effective OSTA interventions 5](#_Toc17193809)

[Preparing a coding book 5](#_Toc17193810)

[Coding sheet and description sheet 5](#_Toc17193811)

[Study sheet 8](#_Toc17193812)

[Coding procedure 8](#_Toc17193813)

[Coding studies 8](#_Toc17193814)

[Extracting the common elements 10](#_Toc17193815)

[Making common element profiles 11](#_Toc17193816)

# Introduction

The following manual describes methods and procedures for identifying Common Elements (CE) among studies selected based on predetermined selection criteria. The manual presumes familiarity with systematically reviewing literature. We recommend conducting a systematic review (or using an existing updated review) to identify interventions for CE analyses. You can tailor this manual based on your project’s needs; a CE analysis can be done comprehensively to acquire fine grained details about common content of interventions, but it can also be conducted more pragmatically and rapid. Please contact the corresponding author if you intend to use or adapt this manual. It is continuously in development and the author would be happy to share updates and would also like to keep track of adaptations.

## What are Common Elements?

Common elements are discrete and distinct content that are shared by a selection of interventions. An intervention can be broken down into several distinct actions or practices - or in other words; *practice elements*. A practice element is a specific practice or action performed during an intervention (such as tutoring, positive reinforcement, or timeout). A *common practice element* is a practice element that is frequently involved in a selection of several interventions aimed to produce an outcome. In a selection of effective interventions, a common practice element is theorized to have particularly potent ability to produce specific outcomes, based on its frequent involvement in interventions that has produced positive effects on specific outcomes. Meaning, one assumes that the common practice element may have an inherent potential to induce change alone or under certain conditions. However, these are hypotheses needed to be tested experimentally.

Common elements can also be specific element characteristics, and combinations of characteristics, that are frequently associated with the common practice element. Inspired by the Parenting and Family-Based Interventions project (PRC), we divide these characteristics into *process elements* and *implementation elements*. We do, however, define them somewhat differently: We define a process element as what characterizes how and under what circumstances the practice element is delivered (such as in a group, at home visit, or using role play). We define implementation elements as discrete strategies used to facilitate or ensure the delivery of practice- and process elements (such as reminders, audit and feedback of performance data, and dynamic training).

Common process- and implementation elements are also based on frequency, by frequently being used together with the specific common practice element in a selection of interventions to address a specific outcome. To identify common practice- process- and implementation elements for specific outcomes, we have developed a methodology that uses coding of interventions in matrices and simple frequency-based detection algorithms. If sufficient information is available from studies, one can also apply statistical techniques (such as meta-regressions) to statistically test how the effect of specific elements are related to outcomes.

## Review process

The review process and reporting procedures should follow the standards of high-quality systematic reviews (e.g. in accordance with the Cochrane Handbook for Systematic Reviews of Interventions or the Center for Reviews and Dissemination Guidance for Undertaking Systematic Reviews). Preferably, a systematic review protocol is registered online and carried out prior or simultaneously with identifying common elements. If you choose a selection of studies based on prior reviews, you can use the AMSTAR checklist to assess the quality of the reviews

# Selecting studies for coding

Different criteria can be applied to determine study selection. During the review process, your PICO’s (populations, interventions, comparisons, outcomes) and design criteria should be clear. If you are mapping the literature to investigate which elements are most commonly used to address specific outcomes, no further criteria is needed. You code all studies included in your review. However, if you e.g. aim to develop a new intervention (or re-design an existing one) or inform educational purposes, you might consider using a valid representation of the best available evidence about the effects of interventions on outcomes you are interested in. Selecting studies that has produced positive effects and/or are classified as evidence-based interventions might then be appropriate, or you can apply specific criteria such as effect sizes. You might also consider giving studies different “weights” based on criteria, such as risk of bias. Arguably, certain types of risks in a risk of bias assessment should be given more weight than others (e.g. blinding of participants in social interventions might be less important compared to random allocation). The same applies to weighting based on use of active or passive comparison conditions. Chances of significant differences between two active conditions are lower than comparing an intervention to nothing. Weighting based on risk of bias and comparison criteria can influence both study inclusion and interpretation of CE results, and should be considered a priori.

## Criteria for study quality and effectiveness used in the common elements review of effective OSTA interventions

As an example, criteria applied in the common elements review of effective out-of-school-time (OSTA) interventions for at-risk children is described below. This review was part of the KOBA-study (development of a new academic intervention).

*Each study included for common elements analyses had to fulfill pre-determined risk of bias criteria. Studies with a majority of low or medium risk of bias scores on the Cochrane Risk of Bias assessment were included. Studies with a majority of high risk of bias scores were excluded. Two review authors (TE and KTH) independently assessed risk of bias in the included studies using the Cochrane Risk of Bias tool (Higgins & Green, 2011). All disagreements were resolved by TE and KTH.*

*Interventions in the included studies could either be labeled as effective or ineffective interventions. For randomized controlled trials; the intervention needed to have a significant effect on a primary or secondary outcome to be labeled effective. For non-randomized controlled and interrupted time series studies the intervention group additionally needed to significantly differ from the comparison group at post intervention on a primary or secondary outcome to be labeled effective. The effects could be either positive or negative (i.e. adverse or harmful). Interventions in included studies that failed to fulfill the abovementioned criteria were labeled ineffective. An outcome needed to be significantly improved in a minimum of three studies to be included in reporting.*

## Limitations due to effectiveness criteria

Using effectiveness criteria has its limitations. Excluding or ignoring non-effective studies can cause bias in the elements. Hence, after you have identified common elements of effective interventions, you should explore if these common elements are also common in ineffective or negatively effective studies and interpret accordingly. A more rigorous approach would include additional coding of non-effective studies; meaning studies where there was no significant difference in effects between experimental and control/comparison conditions on outcomes, and negatively effective studies; meaning studies where the experimental condition has a significant negative/adverse effect on outcomes compared to control/comparison conditions. These coding procedures should be done in separate coding-sheets. A less time-consuming alternative can be to retrospectively review (without coding) the frequency of which the most common elements of effective studies are also used in ineffective or negatively effective studies to nuance interpretation of results. Regardless, all studies should fulfill risk of bias criteria.

## Coding of ineffective interventions in the common elements review of effective OSTA interventions

*As an example, procedures applied for ineffective and negatively effective studies in the OSTA-review is described below.*

*Practice elements in ineffective interventions were also coded. A traditional vote-counting procedure (Bushman & Wang, 2009) was used to determine a frequency count value (FV) accounting for positive, non-significant and negative study results for each common practice element, process- and implementation elements used in combination with the practice element, and common combinations of practice elements. If a common practice element was included in an intervention that did not fulfill effectiveness criteria, a frequency count value of one was deducted from the frequency value of that practice element and process- and implementation elements used in combination with that practice element. If a common practice element was included in an intervention that had a negative effect on outcome, a frequency count value of two would have been deducted from the frequency values. That means that Frequency Count Values (FVs) were calculated by counting the frequency of inclusions in interventions with significant positive effects on target outcomes (Fpos), and subtracting from that number the frequency of inclusion in interventions with non-significant (-1) or negative (-2) effects on target outcomes (Fnon, Fneg, respectively). That gives the following formula: FV = Fpos – (Fnon + 2Fneg). Fneg was given double value because the authors view the chance of an intervention causing harm as outweighing the chance of an intervention causing positive effects. However, no interventions with negative effects were identified in the review.*

*The vote counting procedure was performed to reduce popularity bias, which can be defined as the tendency to include elements that are frequently used in interventions based on the element being perceived as important, regardless of the elements’ effectiveness, appropriateness or appearance in ineffective interventions. Frequency count values are, however, likely to be skewed because of publication bias (Easterbrook et al, 1991).*

# Preparing a coding book

## Coding sheet and description sheet

Before coding you need to create coding sheets tailored to your PICOs. That is, the populations, interventions, (comparisons), and outcomes of interest. The following is a pragmatic procedure to develop coding sheets:

After reviewing a substantial amount of studies during the review process, you should have sufficient insight into content and characteristics of interventions of interest to be able to create an interface coding sheet in Microsoft excel (or similar software). Coding can also be done in SPSS or other statistical packages. To prepare your interface, you can work together with your fellow coder(s) following Consensus Mapping procedures. Together, you can start listing all practice elements you expect to be a part of the interventions included for coding down the Y-axis. That is, all practices or actions performed during an intervention. Then you can split into small groups/dyads, and let each group work on defining each element or divide clusters of elements among the groups/dyads. Then you can review all elements together, discuss, revise and reach consensus on definitions. As you reach consensus, fill out a *description sheet* in your excel book. Some elements and characteristics are ambiguous and require detailed description to prevent misinterpretations from coders and promote reliable coding. Include a description sheet in Excel where all elements and characteristics are defined and described in detail.

It can be beneficial for later interpretation to agree on a categorization of the content. An example could be:

Category: *Parent behavior during tasks*

Practice elements/actions:

- *Observe child performance*
- *Give praise for correct performance*
- *Give praise regardless of performance*
- *Use incentives for correct performance*
- *Give feedback on performance*
- *Correct wrong performance*
- *Repeat performance until correct*
- *Perform task together with child*
- *Perform task together, then let child perform alone*

When you start preparing your interface coding sheet you should be aware that you are making some decisions regarding *level of elements* or *discreteness.* The example above displays a highly discrete level of elements, meaning highly divided or distilled elements. In some cases, it might be more appropriate to define elements on a less refined level. That will make coding less time consuming and promote reliability in coding, however, your results will have less details and be less nuanced. The same example could then be:

Category: *parent behavior during tasks*

Practice elements/actions:

- *Observation with correction*
- *Cooperative performance with correction*
- *Positive reinforcement*
- *Modelling*

When listing expected elements down the y-axis, make sure to leave open rows under each category for any additional elements surfacing during coding (or you could insert new rows each time you need space).

When your Y-axis is done, you start listing relevant characteristics across the X-axis (target outcomes, process- and implementation elements), and carry out consensus mapping with those as well. The characteristics should also be tailored to your PICOs. We recommend using taxonomies and definitions available in the literature, however, we advise training coders to not be constrained by the prepared coding sheet or exciting taxonomies. For instance, implementation elements can be listed and categorized in accordance with the Expert Recommendations for Implementing Change project (ERIC, Powell et al., 2015), however, you can add more implementation elements if you find elements that are not covered by the strategies listed, and you might find that you want to further disentangle the ERIC strategies into more discrete strategies. For instance, in the KOBA-study, the implementation strategy make training dynamic was further disentangled as below.

*Disentanglement of the implementation strategy/element “make training dynamic”*

| **Discrete implementation element** | **Definition** |
| --- | --- |
| Training using role play | Actively engaging trainees in changing their behavior to act out a role for the purpose of learning |
| Interactive training | Learning occurs through mutual actions between trainers and trainees. In interactive training, teaching and learning are interrelated, and trainees learn through a process of reflection through personal experience. |
| Training with feedback | Any form of training with real time feedback on performance or behavior from a trainer or peer |
| Training with multiple training techniques | Any form of training where two or more defined training techniques are combined |
| Other dynamic training techniques | Any form of dynamic training not applicable to the training strategies above |

The first characteristics listed on the X-axis should always be your outcome measures, because the common elements will later be revealed under these rows. Next you list your characteristics and process elements under appropriate categories. Studies are generally poor at reporting implementation strategies, thus, implementation elements are listed at the end of the X-axis. Here are some examples of characteristics, process elements and implementation elements:

- Different outcome measures (e.g. internalizing symptoms, externalizing symptoms, quality of life ect)
- Characteristics of target population (e.g. age, gender, etc)

*Process elements*

- Content deliverer (e.g. parent, volunteer, paraprofessional, professional, computer, application etc.), Delivery method (e.g. 1on1 instruction, group instruction, 1on1 interaction, group interaction), Context of delivery (e.g. In home, at school, at clinic etc.)
- Delivery duration (e.g. intensive and short, intensive over time, limited and short, limited over time). Theoretical basis/discipline (e.g. cognitive behavioral, transactional, attachment theory etc.), Materials (e.g. manuals, educational books, worksheets etc.)

*Implementation elements*

- Audit and feedback (e.g. use of performance data such as fidelity audit, fidelity monitoring, fidelity feedback)
- Assess readiness (e.g. assess implementation climate, barriers and facilitators, implementation leadership)
- Tailor to context (e.g. promote local adaptations, use local stakeholders in intervention development, translate material to local languages)

A Microsoft Excel template that includes some characteristics and elements for illustration and re-use can be obtained by request to corresponding author. This template is continuously being updated with new features, so make sure you have the latest. You are encouraged to use these for comparison purposes. Nevertheless, you should tailor characteristics and elements to your study. Make sure to remove excess rows and columns to promote user-friendliness.

With time and resources available, you should consider developing the coding- and description sheets in collaboration with relevant experts and stakeholders. Doing so can be time consuming but will likely improve the common element’s relevance and applicability.

## Study sheet

The template also includes a study sheet where all studies included for coding should be listed and given a unique number. This is also where you provide information about outcome measures of interest in the study, and where coding data is imputed to gather data on rater reliability. Default listing of outcomes are under the “A” column. If a study addresses two or more outcomes of interest, the second outcome is listed under “B”, the third under “C” and so on. If you want to code elements for two or more outcomes in one study simultaneously, this outcome letter will be attached to the unique study number (e.g. 13ab,).

# Coding procedure

## Coding studies

After you have prepared the coding interface you should do some iterations of pilot coding for each coder, and then you can start coding. There should be at least two coders who code all studies independently in their own coding sheet. When they complete the coding, they resolve any differences in added practice-, process-, and implementation elements and conflicts in coding through discussions. It might be practical to allow one coder to complete coding before the other one starts, seeing as the list of elements on the coding sheet will increase during coding. By coding sequentially, you will limit the differences in added elements between the sheets at the end. Or, you can discuss new elements with fellow coders as they surface, and review completed studies again to look for the new element.

Each study provides varying amounts of information about the content of the experimental interventions. When available, you should use the intervention manual to get more detailed information (or other available information about the intervention). Contact study authors if the intervention manual is not available.

1. Before starting the coding, pilot the coding sheet together with your co-coder(s). Make sure you interpret and understand the elements and characteristics similar. Thorough piloting will likely promote inter-rater reliability.
2. When coding a study, you should have all information describing the content and characteristics of the intervention available in front of you. You start by reading through the content of the intervention.
3. You then look at the list of practice elements on your coding sheet and find a practice element included in the intervention.
4. When you find an element, you check the cell that corresponds with every characteristic, process element, and implementation element present in the intervention (you will have to search your study information for each characteristics and elements). You check the cell by writing in the study’s unique number followed by a comma (e.g. “14,”). Put in another way: You first find the practice element on the Y-axis, and you follow the row of the practice element all the way through to the end of the X-axis and check every characteristic and element that describes an aspect related to the practice element in the study.

**Exception:** Some studies give fine details on interventions and requires you to code different per element in the intervention. For instance, some modular or tailoring interventions might have different elements of an intervention used under certain circumstances (e.g. exposure only used with older age groups and not children under age of 12, or tablet game only used for math tutoring, and not tutoring in reading). In such circumstances, make sure you do not “auto-check” characteristics and process elements on all practice elements in an intervention. Rather, code characteristics and process elements per practice element in the intervention.

1. If the study has intervention elements targeting different outcomes, you attach the outcome letter next to the unique study number when you check a cell (e.g. “14b,”)
2. You repeat this with as many practice elements you can find on your list.
3. If a practice element identified in the article or manual is not on your Y-axis list, you simply add it to the most appropriate category or a “other” category. You can also add characteristics to the X-axis if a significant characteristic, process element or implementation element is missing.
4. When there are no more practice elements to code, you count the number of codings you made for the study (you can use the search function in excel), and fill in the number in the study sheet under the row “number of codings”. Then you move on to the next study and repeat on the same coding sheet until you have coded all studies, which means that certain cells will get increasing amount of numbers (e.g. “14b,17,19,24a,29c,32,”)
5. After each coder has completed coding, their independent coding sheets are to be combined into one sheet. Any differences in coding is regarded as a conflict and should be resolved be reaching consensus before coding sheets are combined. Unresolved conflicts should be resolved by a third coder.
6. Make sure to register amount of initial conflicts and third party resolved conflicts to gather reliability data (in the study sheet).
7. (If you have only coded studies meeting effectiveness criteria, it is encouraged to repeat step 2 through 10 on ineffective interventions, and then studies with negative effects)

## Extracting the common elements

After coding is completed and all conflicts resolved, the result is one sheet with practice elements on the Y-axis, characteristics, process elements, and implementation elements on the X-axis, and a lot of numbers in a matrix (and one sheet on studies without effect, and one on studies with negative effects if you have completed step 11). This is when the most common elements are possible to extract using a simple frequency-based algorithm for detecting common elements and common combinations of elements. Follow these rules carefully:

1. Locate the cell under the “effect on outcome” characteristics that has the highest amount of numbers in it. The practice element this cell corresponds with is a common practice element for that particular outcome. Make a duplicate of the coding sheet and name it the common practice element.
2. Next, note all the numbers in the common practice element (next to practice element, under outcome).
3. In the new sheet, remove all numbers coded that are not included in the common practice element cell (use the search function to save time, but be precise when you delete numbers).
4. What you have now is a matrix showing a common practice element, and how frequently this element has been used in combination with specific characteristics, process elements, implementation elements, and other practice elements.
5. For illustrative purposes, consider color-coding the cells with most frequent practice elements and combinations with process- and implementation elements (the cells with the most numbers) using different color-nuances. E.g. if you have reviewed ineffective studies and calculated frequency count values (Fv), color Fv of 15+= deep red, Fv of 13-14= lighter red, Fv of 11-12= orange, Fv of 9-10= light orange, Fv of 7-8= yellow, Fv of 5-6=green and so forth. The color nuances will make it easier to detect patterns of common combinations of elements, and elements that are rarely used in common combinations.
6. Repeat step 1 to 5 with all the most common practice elements in your coding sheet. What degree of “commonness” that qualifies as common enough to be considered a common element is a matter of interpretation an should be discussed with co-authors. Some factors that should be considered are the total amount of included studies, study quality, and purpose of the common elements review.
7. If you applied effectiveness criteria; you should cross check to what degree your common elements and combinations of elements are present in studies without effects and with negative effects and interpret results according, for instance calculating frequency count values (see page 3 for example).
8. There are several ways to extract and report the data. One reporting example can be found in the article referenced at the title page. You can also make copy of each color-coded common element sheet, remove all rows and columns without a color (e.g. frequency value of less than five), replace study numbers with frequency value, and export the coding sheet into an image showing color coded frequency values of common practice elements combined with common characteristics, process- and implementation elements.
9. You can also trace common elements and characteristics back to the original studies and investigate if there is sufficient data available to test some of these common elements statistically.

Example of extraction procedure in the KOBA-study:

*Common practice elements were identified in the master matrix based on frequency counts. The 25% most frequent practice elements of effective interventions was classified as common practice elements. A separate matrix was created for each common practice element, where all unique IDs from studies where the practice element was not included was removed from every cell in the matrix. Thus, the remaining matrix included elements and characteristics associated with the specific common practice element in effective studies only. The same frequency counts denoted the most common process- and implementation elements used in combination with the common practice element, and also the most frequent combinations of common practice elements. The common elements and combinations of elements were given a frequency count corresponding with the number of times the elements and combinations were included in studies with significant positive effects on target outcomes (reading, math, grade point average). Next, ineffective studies were reviewed and if ineffective interventions included a common practice element, a frequency count was deducted from this common practice element and all common process- and implementation elements and characteristics used together with the common practice element.*

We have also developed a coding system for SPSS with syntaxes for extracting results. This system eases the coding procedure and likely reduce coding errors, however, extracting results from very fine-grained combinations of common elements and characteristics are more difficult in SPSS compared to a MS Excel matrix.

# Making common element profiles

In the KOBA-study, the common elements analyses were a first step in the development of a new lean intervention for providing academic support to children in child welfare. The purpose was to combine the most frequent common practice-, process-, and implementation elements to co-design a flexible and locally tailored intervention together with stakeholders and users in child welfare services. In order to appropriately disseminate the results of the analyses to both researchers, practitioners, parents and youths, the results were described in plain language common elements profiles as a tool informing co-design of the intervention. These profiles were created in the following steps:

1. A common language exercise was conducted to create a glossary of potentially difficult and ambiguous terms that were likely to be used in the co-design process. Terms were brainstormed and put on a list (e.g. “common elements”, “implementation”, “client/user/children/parents”), and co-design participants worked through each word, reached consensus on what term they would use precedingly, and agreed on a definition of the term. The glossary was displayed in a screen in every co-design workshop, and any new terms in need of clarification identified during a workshop was processed in the same way and added to the glossary. This was done to promote a common understanding of frequently used terms, equal participation in co-design, and to avoid potentially offensive terms. The exercise also served as interactive training and knowledge exchange between co-design participants.
2. Using wording from the glossary, common elements profiles was created to disseminate results from common elements analyses. Frequencies of practice-, process- and implementation elements were displayed graphically, as well as frequencies of study characteristics associated with common elements, and also common combinations of elements. The most common elements and combinations were also explained in plain language text, accompanied by an explanation of the interventions the elements had been used in.
3. The profiles were handed out to co-design participants, and they were given an interactive presentation of the results. This provided the participants with an understanding what elements were most frequently used in effective interventions, how they were most frequently delivered, under what circumstances, how they were implemented, and what combinations that were most frequent.
4. The co-design procedure that followed will be thoroughly described in a future publication (Engell et al., in prep)

Engell, T., Follestad, I. B., Andersen, A., & Hagen, K. A. (2018). Knowledge translation in child welfare—improving educational outcomes for children at risk: study protocol for a hybrid randomized controlled pragmatic trial. *Trials*, *19*(1), 714.
