# Supplementary material for: Common Elements of Practice, Process and Implementation in Out-of-School-Time Academic Interventions for At-risk Children: a Systematic Review
Source: Prev Sci. 2020 Feb 4;21(4):545–56. doi: 10.1007/s11121-020-01091-w (PMC7162823; doi:10.1007/s11121-020-01091-w)
Supplement: Supplementary file 4 — (PDF 165 kb) [file 11121_2020_1091_MOESM4_ESM.pdf]

Table 1 *Brief summary of study characteristics*

Study reference	Sample	Setting	Intervention	Study design	Effects on/ineffective[a]
Andersen et al, 1979	Total N: 36 children<br>Mean age: 6.9 years old (range 6.3 – 8.1 years old)<br>Gender: 19 boys, 17 girls	Country: USA<br>Setting: unclear location with tutors after school	After school tutoring in reading	RCT	Reading
Black and Somers, 2009	Total N: Math cohorts: 1144 reading cohorts: 905<br>Mean age: 8.6 (grade 2-5)<br>Gender: Math cohort 1: intervention; 47% male, control; 43% male. Math cohort 2: intervention; 42% male, control; 46% male. Reading: intervention; 53% male, Control; 47% male	Country: USA<br>Setting: at a primary school after school hours	Mathletics (math tutoring) and Adventure Island (reading tutoring)	RCT	Math, ineffective on reading
Blachman et al, 2004	Total N: 69<br>Age: second and third graders<br>Gender: 42 boys, 27 girls	Country: USA<br>Setting: Unclear	Intensive tutoring in reading	RCT	Reading
Bridges, 2011	Total N: 232<br>Age: third graders, not further specified<br>Gender: Unclear	Country: USA<br>Setting: After school at a primary school	After school group tutoring	Quasi exp.	Reading
Bronstein et al., 1998	Total N: 23<br>Age: 9-12, with mean of 10.7 years<br>Gender: Majority of girls, not further specified	Country: USA<br>Setting: Unclear	Group parent training	RCT	Grade point average
Carbone, 2009	Total N: 316<br>Age: Fifth, sixth, seventh, and eight graders<br>Gender: Unclear	Country: USA<br>Setting: Public middle school after school hours	Reading Labs, Specific Skill Series Labs and/or Skills tutor (reading). America's Choice Mathematical Navigator	Quasi exp.	Math, ineffective on reading
Cid, 2014	Total N: 54<br>Age: intervention group: 6.32, control group: 6.48<br>Gender: All boys	Country: Uruguay<br>Setting: After school program	Los Pinos, after school program	RCT	Ineffective on GPA
Chassen, 1978	Total N: 60<br>Age: seventh – eighth graders<br>Gender: Unclear	Country: USA,<br>Setting: at home	Parent training and homework routines	RCT	Reading
Cloward, 1966	Total N: 513<br>Age: 9 years (54% in experimental and 58% of control are fourth graders)<br>Gender: 47% girls, 53% boys in experimental group. 50% girls and 50% boys in control group	Country: USA<br>Setting: after school in tutorial centers in elementary schools	Peer tutoring	RCT	Reading
Cole, 2006	Total N: 36	Country: USA	Web-based tutoring	RCT	Reading

	Age: range from 8 years and 6 months to 10 years and 4 months<br>Gender: g=19, b=17.	Setting: Public elementary school before school hours			
Duckworth, 2013	Total N: 77<br>Age: fifth graders<br>Gender: unclear	Country: USA<br>Setting: Elementary school	Mental Contrasting with implementation Intentions	RCT	GPA
Eash, 1981	Total N: 531<br>Age: 5 and 8-year old's<br>Gender: unclear	Country: USA<br>Setting: Child-parent centers	Child-Parent Expansion Program	Quasi exp.	Math, ineffective on reading
Ellis, 1996	Total N: 82<br>Mean age: 8.03<br>Gender: 50% female	Country: USA<br>Setting: Parent training at school, tutoring at home	Parent training (reading instructions, paired reading, positive reinforcement)	RCT	Ineffective on reading
Esters, 1983a	Total N: 33<br>Age: Third and fourth graders<br>Gender: 24 boys, 9 girls	Country: USA<br>Setting: Unclear	Systematic Training of Effective Parenting	RCT	GPA
Esters, 1983b	Total N: 33<br>Age: Third and fourth graders<br>Gender: 24 boys, 9 girls	Country: USA<br>Setting: Unclear	Self-Esteem Program	RCT	GPA
Fantuzzo, 1995	Total N: 72<br>Mean age: 10 years and seven months<br>Gender: unclear	Country: USA<br>Setting: Public school classrooms	Parent training in parental involvement	RCT	Math
Feldman, 1983	Total N: 16<br>Mean age: 11.1<br>Gender: Only reported for control group: 7 boys, 1 girl	Country: Canada<br>Setting: At home	Parent training and parent tutoring	Non-RCT	Reading
Flynn, 2012	Total N: 177<br>Mean age: 10.7<br>Gender: Intervention group: 50% girls, Control: 57.1% girls	Country: Canada<br>Setting: Foster homes	Teach Your Children Well program	RCT	Reading and math
Harper, 2012	Total N: 68<br>Mean age: 10.1<br>Gender: Intervention group: 42% girls, control: 43% girls	Country: Canada<br>Setting: Foster homes	Group based version of Teach your Children Well program	RCT	Reading
Harpine, 2009	Total N: 54<br>Age: first to third graders<br>Gender: 32 boys, 22 girls	Country: USA<br>Setting: After school center	Camp Sharigan, tutoring and self-efficacy training	RCT	Reading
Mehran, 1988	Total N: 76<br>Age: First graders<br>Gender: Unclear	Country: USA<br>Setting: At home	Parent training and parent tutoring	RCT	Reading

Morris, 1990	Total N: 34<br>Age: Second and third graders<br>Gender: Unclear	Country: USA<br>Setting: After school building close to school	Howard Street Tutoring Program	RCT	Reading
Morrow, 1997	Total N: 56<br>Age: 19 first graders, 17 second graders, 18 third graders<br>Gender: Unclear	Country: USA<br>Setting: At home and at school	Parent training (school involvement and literacy activities)	RCT	Reading
Munoz, 2008	Total N: Reading cohort: 1085, math cohort: 916<br>Age: fourth-, fifth-, seventh-, eighth- and eleventh graders<br>Gender: reading cohort: 45.49% female, math cohort: 49.91% female	Country: USA<br>Setting: at home, at school after hours, at after school program	Various Supplemental Educational Services	Quasi exp.	Ineffective on reading and math
Munoz, 2011	Total N: 862<br>Age: Second and third graders<br>Gender: Unclear, fourth to eight graders	Country: USA<br>Setting: at home, at school after hours, at after school program	Various Supplemental Educational Services	Quasi exp.	Ineffective on reading and math
Powell-smith, 2000	Total N: 38<br>Mean age: Seven years, 11 months<br>Gender: 39% girls	Country: USA<br>Setting: at home, at school after hours, at after school program	Home based parent tutoring	RCT	Ineffective on reading
Rasinski, 2005	Total N: 30<br>Mean age: 6<br>Gender: 16 girls, 14 boys	Country: USA<br>Setting: at home	Fast Start Parent tutoring	RCT	Reading
Reutzel, 2006	Total N: 144<br>Age: first graders<br>Gender: unclear	Country: USA<br>Setting: at home	Parent training and parent tutoring	Quasi exp.	Reading
Schinke, 2000	Total N: 283<br>Mean age: 12.3<br>Gender: 40% girls	Country: USA<br>Setting: After school program (boys and girls club of America)	After school boys and girls club	Quasi exp.	Ineffective on GPA, math and reading
Shuck, 1983	Total N: 150<br>Age: Third to fifth graders<br>Gender: Unclear	Country: USA<br>Setting: at home	Parental school involvement at home	RCT	Reading
Tolan, 2004	Total N: 424<br>Age: 5-6 years<br>Gender: 49% girls	Country: USA<br>Setting: at school and home	SAFEchildren; parent group training and Fast Track tutoring	RCT	Reading

Tolan, 2009	Total N: 348<br>Age: Fourth graders<br>Gender: 54.6% girls	Country: USA<br>Setting: at home	Booster intervention of SAFEchildren	RCT	Reading
Trovato, 1980	Total N: 90<br>Mean age: 9<br>Gender: Unclear	Country: Canada<br>Setting: at school and home	Peet tutoring and home-based positive reinforcement	RCT	Reading
Weine, 1993	Total N: 147<br>Age: Third to fifth graders<br>Gender: 48% girls	Country: USA<br>Setting: at school and home	Parent training and parent tutoring at home	RCT	Reading
Wise, 1972	Total N: 38<br>Mean age: 8.45<br>Gender: 23 boys, 15 girls	Country: USA<br>Setting: at home	Home-Instructors; parent or sibling tutoring at home	Non-RCT	Reading
Zosky, 2003	Total N: 77<br>Age: Fourth graders<br>Gender: Unclear	Country: USA<br>Setting: after school center or at school	After school program; homework supervision and positive reinforcement	Quasi exp.	Math and GPA, ineffective on reading

[a] Being labeled as ineffective refers to lack of statistically significant effects. Results could be positive, but not statistically significant.
