# Supplementary material for: Common Elements of Practice, Process and Implementation in Out-of-School-Time Academic Interventions for At-risk Children: a Systematic Review
Source: Prev Sci. 2020 Feb 4;21(4):545–56. doi: 10.1007/s11121-020-01091-w (PMC7162823; doi:10.1007/s11121-020-01091-w)
Supplement: Supplementary file 5 — (PDF 102 kb) [file 11121_2020_1091_MOESM5_ESM.pdf]

Table 2. *Frequencies of practice elements in OSTA interventions not meeting criteria as common elements*

Practice element	Frequency	Practice element	Frequency	Practice element	frequency	Practice element	Frequency
Discussion[a]	8	Use of homework contracts	3	Letter training	2	Playing math game	1
Playing reading game	8	Use of homework schedules	3	Math computation: addition and subtraction	2	Training in geometry	1
Modification of motivation	8	Digital math computation	3	Use dialogs as learning technique[a]	2	Training in probability	1
Modification of attitudes	8	Modeling[a]	3	Training in parental involvement at school	2	Math computation on paper	1
Monitor performance[a]	8	Homework support: only alter homework environment (not structure or routines)	2	Role playing[a]	1	Math computation in probability	1
Check or review homework	6	Use of behavioral contracts		Use of consequences/punishment	1	Math computation in geometry	1
Facilitate communication/collaboration between home and school	6	Training in math measurement	2	Increase access to books	1	Web-based/computerized reading program	1
Writing texts	5	Observation of learning[a]	2	Reading alone	1	Parent self-esteem training	1
Unspecified parent training	5	Math computation: multiplication and division	2	Storytelling	1	Parent attentiveness training	1
Unstructured tutoring	5	Math computation: fractions and equations	2	Reading club	1	Adlerian parent training	1
Spelling	5	Use of homework diaries	2	Communication training	1		
Math computation aggregated	4	Social skills training	2	Transactional analysis	1		

1. [a] Elements coded as both practice- and process elements because they have been used interchangeably as a discrete practice and as a technique to deliver another practice element
