# Supplementary material for: Common Elements of Practice, Process and Implementation in Out-of-School-Time Academic Interventions for At-risk Children: a Systematic Review
Source: Prev Sci. 2020 Feb 4;21(4):545–56. doi: 10.1007/s11121-020-01091-w (PMC7162823; doi:10.1007/s11121-020-01091-w)
Supplement: Supplementary file 6 — (PDF 190 kb) [file 11121_2020_1091_MOESM6_ESM.pdf]

Table 3 *Overlap of practice elements in OSTA and psychosocial interventions*

**Reference**	**Interventions**	***positive reinforcement (praise, incentives)***	***psychoeducation***	***goal setting***	***correction and feedback***
Engell et al., 2020	Out-of-school-time academic interventions for primary school children at risk	14[a] of 36[b]	To children: 1 of 36<br>To caregivers: 8 of 30	9 of 36	7 of 36
Sutherland et al., 2018	Social, emotional, and behavioral interventions for young elementary school students	Reinforcement: 11 of 103<br>Praise: 9 of 103<br>Rewards: 8 of 103	-[c]	-	8 of 103
Lawson et al., 2018	Social emotional learning programs for elementary school children	-	-	9 of 14	-
Brown et al., 2017	Psychosocial interventions for children and youth affected by armed conflict	8 of 25	To children: 21 of 25<br>To caregiver 13 of 25	6 of 25	-
Van der Put et al., 2017	Child maltreatment interventions	-	8 of 121	-	-
Mcleod et al., 2016	Social, emotional, and behavioral interventions in early childhood classrooms	Praise: 15 of 49<br>Rewards: 14 of 49	-	-	2 of 49
Becker et al., 2015	Engagement interventions in children's mental health services	-	Reported without frequency	-	-
Lindsey et al., 2014	Treatment engagement interventions in children's mental health services	11 of 40	33 of 40	9 of 40	-
Boustani et al., 2014	Adolescent prevention programs for health-related outcomes	-	To children: 36 of 58	14 of 58	-
Chorpita et al., 2009	Evidence based psychotherapy treatments for children and adolescents	Praise: 62 of 232<br>incentives: 66 of 232	To child: 60 of 232<br>To parent: 65 of 232	46 of 232	-
Garland et al., 2008	Evidence-based psychosocial treatment for children's disruptive behavior	Reported without frequency	Reported without frequency	Reported without frequency	-

[a] Frequency count [b] total studies included in common elements review [c] practice element not reported in the study indicated by "-"
